# Mapping functional non-coding variation in individual human genomes through haplotyping, multiomics, and deep learning

**DOI:** 10.1038/s41467-026-72392-x

**Published:** 2026-04-29

**Authors:** Mikhail D. Magnitov, Robin H. van der Weide, Aster F. Witvliet, Miguel Hernández-Quiles, Moreno Martinović, Hans Teunissen, Luca Braccioli, Michiel Vermeulen, Elzo de Wit

**Affiliations:** 1 Division of Gene Regulation, The Netherlands Cancer Institute, Amsterdam, The Netherlands; 2 Division of Molecular Genetics, Oncode Institute, The Netherlands Cancer Institute, Amsterdam, The Netherlands; 3 Department of Molecular Biology, Radboud Institute for Molecular Life Sciences, Oncode Institute, Radboud University Nijmegen, Nijmegen, The Netherlands

**Keywords:** Gene expression profiling, Haplotypes

## Abstract

Most genetic variants in the human genome reside in non-coding regions, where they can perturb regulatory element activity to influence gene expression, thereby contributing to various phenotypes and diseases. However, identifying functionally relevant non-coding genetic variation remains challenging. Here we integrate personal genomics, allele-specific gene regulation, and deep learning predictions to map the impact of non-coding variation in its native allelic and regulatory context. Leveraging whole-chromosome haplotypes and allele-specific analyses, we establish regulatory links within individual human genomes, enabling us to evaluate functional consequences of both common and rare variants. We identify and validate hundreds of cell-type-specific transcription factor binding events disrupted by genetic variants, revealing known and novel mechanisms that underlie allele-specific chromatin accessibility and gene expression. Using this framework, we discovered a rare variant that disrupted an OCT2 binding site within a distal enhancer, thereby modulating the expression of *PIK3R5* gene. Our study establishes a generalisable strategy for interpreting non-coding regulatory variation, enabling systematic dissection of variant effects across diverse biological systems and offering a framework to investigate disease mechanisms.

## Abstract (web summary)

How non-coding mutations in DNA contribute to phenotypes is a largely unresolved question. Here the authors integrate personal genomics and machine learning to identify hundreds of genetic variants that affect gene expression.

## Introduction

The vast majority of human genetic variation, such as single-nucleotide polymorphisms (SNPs), insertions and deletions (indels), resides in non-coding regions of the genome^1,2^. These regions contain regulatory elements, such as enhancers and promoters, which consist of DNA motifs that are recognised by transcription factors (TFs), which regulate gene expression^3^. Genetic variants that overlap with TF binding motifs can disrupt the function of regulatory elements, affecting the expression of target genes^4,5^. These perturbations underlie many variant associations implicated in phenotypic traits and disease susceptibility^4–6^.

In human genetics, the links between genotype and phenotype are primarily identified through genome-wide association studies (GWAS) and expression quantitative trait locus (eQTL) mapping. These studies play a pivotal role in connecting non-coding variants to gene expression and phenotypic traits across individuals^7,8^. Both GWAS and eQTL studies require large cohorts to establish associations, resulting in large regions of the genome being correlated with a feature of interest due to linkage disequilibrium^9^. Due to the nature of association studies, the identified genetic links remain correlative, which precludes the establishment of direct causal relationships^10,11^.

To disentangle correlated variants in linkage disequilibrium and prioritise candidate loci identified by GWAS and eQTL studies, a range of statistical fine-mapping methods can be employed^9,12–14^. Integrating additional large-scale data on genome structure and regulatory elements locations can help to further narrow down the pool of candidate associations^15–20^. While these approaches provide a set of credible variants, further functional validation remains essential to understand their cell-type specificity, elucidate the regulatory mechanisms through which these variants act, and establish causality for downstream regulated genes.

More recently, machine learning models have been trained on genomics data to predict the effect of variants on regulatory elements activity and gene expression from DNA sequence^21–31^. These models offer a promising way to understand the functional outcomes of nucleotide base substitutions in a cell-type-specific manner. This enables another variant prioritisation strategy and reduces the number of genetic variants that need to be assessed experimentally. However, substantial data volumes and computational resources are often necessary for training these models, and their performance on predicting perturbations is not fully established, primarily due to the scarcity of available benchmarking datasets^32–37^.

To validate the effects of candidate variants on gene expression, massively parallel reporter assays (MPRAs) that experimentally assess the activity of regulatory elements are widely used^38–41^. However, due to their episomal nature, MPRAs do not represent the endogenous chromatin context. Therefore, introducing the desired variants through genome editing remains the ultimate validation experiment that allows variant causality to be established^42–44^. Nevertheless, with only a few exceptions^45–47^, genome editing remains extremely low-throughput. Moreover, current validation strategies are often constrained by the limited availability of physiologically relevant transfectable cell types and are usually not feasible to implement in vivo.

Alternative strategies are therefore required to help overcome the limitations of population-based methods, establish benchmarks for predictive models and minimise the need for extensive experimental validation. In this regard, analyses that exploit naturally occurring genetic variation in diploid human genomes provide a robust means of evaluating variant function within its native allelic and regulatory context. Comparing the differences in chromatin features and gene expression between alleles provides an unbiased view of the impact of functional variants, including de novo and rare non-coding variants^48–52^. This makes such an approach highly relevant from both fundamental and clinical perspectives^53–58^.

In this study, we combined whole-chromosome haplotyping, multiomics and deep learning to map functional non-coding variants and their regulatory mechanisms in individual human genomes. Using linked-read sequencing and Hi-C, we reconstructed whole-chromosome haplotypes for five individuals and integrated them with chromatin accessibility and transcription data to identify hundreds of allele-specific regulatory elements and genes. To understand the impact of variants on regulatory element activity, we trained a deep learning model and estimated variant effects at the base-pair resolution, revealing hundreds of perturbed transcription factor binding motifs, which we validated using publicly available datasets. Finally, we associated the perturbed regulatory elements with genes, reconstructed multiple known variant-gene associations and discovered a novel rare variant that modulates the expression of the *PIK3R5* gene by disrupting an OCT2 binding motif. Our framework enables functional annotation of non-coding variants in individual human genomes, with potential applications in the elucidation of genetic disorders and personalised medicine.

## Results

### Linked-reads and Hi-C reconstruct whole-chromosome haplotypes with high accuracy

Understanding how genetic variants influence gene expression requires separating *cis*- from *trans*-acting gene regulatory effects. Combining haplotyping, which allocates variants to their respective alleles, with allele-specific analyses reduces the effects of *trans*-acting confounders, such as regulatory proteins or RNA, and provides a clearer view of the impact of *cis*-linked genetic variation. Therefore, at the level of single individuals, it becomes feasible to attribute changes in gene expression to regulatory elements and the genetic variants within them^59^.

**Fig. 1:**
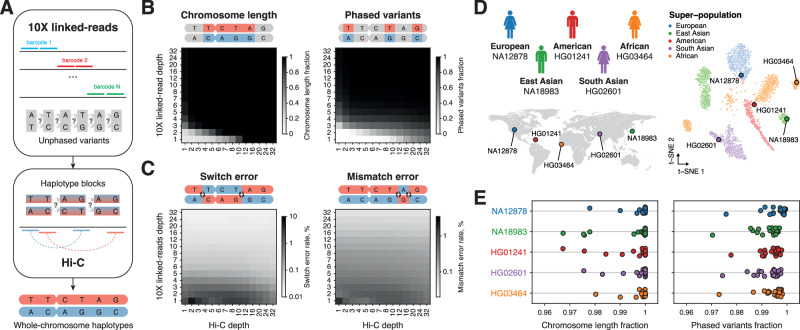
Whole-chromosome haplotypes of genetically diverse individuals phased using linked-reads and Hi-C. **A** Schematic of combining 10X linked-reads and Hi-C data to obtain whole-chromosome haplotypes. First, during the 10X linked-reads sequencing, the genome is partitioned into large fragments with assigned barcodes, which allows local phasing of the variants originating from the same fragment into haplotype blocks. Second, the chromatin confirmation data measured by Hi-C is used to assemble the haplotype blocks into whole-chromosome haplotypes by utilising within-haplotype interactions, which are much more prevalent than between-haplotype interactions. **B** Evaluation of the fraction of chromosome length and the fraction of phased heterozygous variants obtained during the haplotype phasing procedure using 10X linked-reads and Hi-C data subsampled to different genome coverages for the NA12878 individual. **C** Evaluation of switch and mismatch error rates obtained during the haplotype phasing procedure using 10X linked-reads and Hi-C data subsampled to different genome coverages for the NA12878 individual. The obtained haplotypes were compared to the high-confidence phased variants of NA12878 from Platinum Genomes. **D** The overview of the individuals whose lymphoblastoid cell lines were selected for this study from the 1000 Genomes Project (left panel). The sampled locations are indicated on the geographical map. The human figures and geographical map were created using Adobe Illustrator and the Matplotlib Basemap library, respectively. The t-distributed stochastic neighbour embedding (t-SNE) of the 1000 Genomes Project samples based on the genotype matrix (right panel). The samples are coloured according to their super-population of origin, and the selected individuals are indicated on the t-SNE embedding. **E** Phasing metrics for whole-chromosome haplotypes reconstructed for the five selected individuals. The fraction of chromosome length and fraction of heterozygous variants phased per individual are shown. Each data point represents values for a single chromosome.

To link distal regulatory variants to gene expression in an allele-specific manner across large genomic distances, we set out to reconstruct whole-chromosome haplotypes for individual human genomes. Previous studies have shown that a combination of dense local and sparse chromosome-scale phasing methods produces the highest-quality haplotypes^60^. Of the methods assessed, 10X linked-reads and Hi-C exhibited the lowest error rates^61^, prompting us to employ a combination of these technologies (Fig. 1A). However, in a benchmark study that assessed different sequencing methods for haplotyping, a combination of linked-reads and Hi-C was unable to produce whole-chromosome haplotypes, leaving gaps at the centromere^61^.

To ascertain whether this limitation could be overcome using more recent variant phasing methods, we conducted a subsampling analysis using publicly available 10X linked-read and Hi-C data for the NA12878 individual^62–64^. Using the HapCUT2 algorithm^65^, we obtained whole-chromosome haplotypes with > 90% phased variants and a mismatch rate of <0.5%, even at low ~ 5X genome coverage (Fig. 1B; see “Methods”). Increasing the sequencing depth to ~ 30X improves accuracy, reducing both the mismatch and switch error rates to below 0.05% (Fig. 1C). Similar phasing results, albeit with slightly higher error rates, were produced using the TELL-seq^66^ and stLFR^67^ linked-reads technologies (Supplementary Fig. 1). Finally, we applied HapCUT2 to publicly available data from three unrelated individuals and three parent-child trios, whose diploid genomes had previously been assembled^68^. This yielded high-quality haplotype phasing with low error rates per chromosome (Supplementary Fig. 2; Supplementary Data 1 and 2; see “Methods”).

Having confirmed the feasibility of using linked-reads and Hi-C to accurately reconstruct whole-chromosome haplotypes, we proceeded to select lymphoblastoid cell lines (LCLs) from the 1000 Genomes Project for our study^1^. To maximise genetic diversity, we selected select five individuals representing different super-populations: NA12878 (European), NA18983 (East Asian), HG01241 (American), HG02601 (South Asian), and HG03464 (African) (Fig. 1D; see “Methods”). We generated 10X linked-reads and Hi-C data for the LCLs of these individuals and reconstructed their whole-chromosome personal genomes, phasing ~ 99.5% of SNPs and ~ 99% of indels (Fig. 1E and Supplementary Data 2). Together, these phased personal genomes provide a robust foundation for the subsequent allele-specific analyses of regulatory variation and its functional consequences.

### Whole-chromosome haplotypes enable allele-specific analysis of gene regulation

**Fig. 2:**
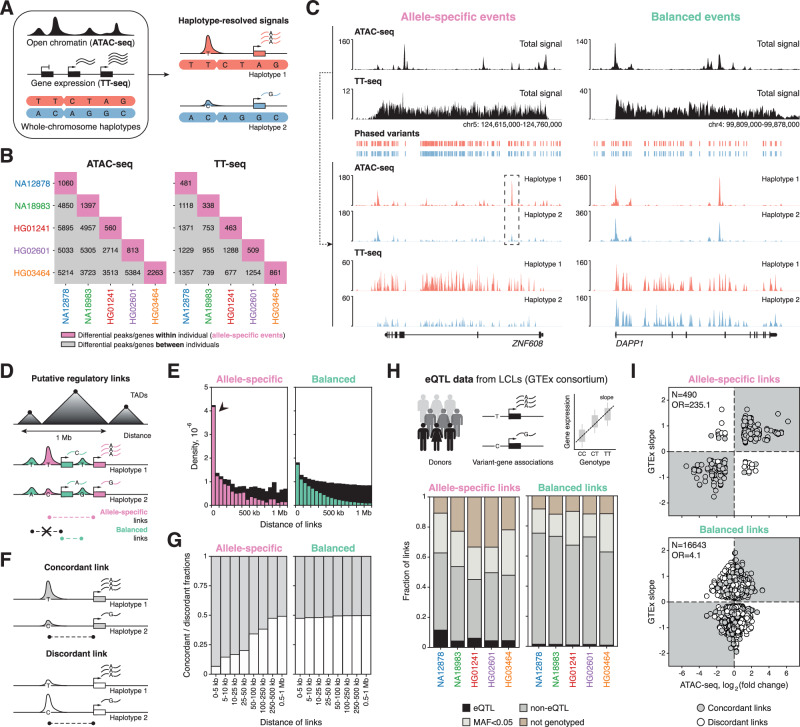
Quantifying allele-specific chromatin accessibility and transcription to infer regulatory links. **A** Schematic of ATAC-seq, TT-seq, and whole-chromosome haplotypes integration to obtain haplotype-resolved chromatin accessibility and gene expression signals. **B** Heatmap showing the number of differential ATAC-seq peaks (left panel) and TT-seq genes (right panel) identified within (in pink) and between (in grey) individuals. **C** ATAC-seq and TT-seq at allele-specific (left panel, data from HG03464 individual) and balanced (right panel, data from HG01241 individual) events at the *ZNF608* and *DAPP1* genes, respectively. The dashed rectangle indicates the position of the allele-specific open chromatin peak. **D** Schematic showing the construction of putative allele-specific (in pink) and balanced (in turquoise) regulatory links using genomic distance and TAD boundaries as constraints. Note that both genes and peaks may be involved in multiple links. **E** Histograms showing the distances between open chromatin peaks and genes comprising allele-specific (right panel) and balanced (left panel) regulatory links. Links that are not located within the same TAD are highlighted in black. The arrow indicates links with a distance below 50 kb. The plot shows the combined data from all five individuals. **F** Schematic showing the categorisation of putative regulatory links as either concordant or discordant. **G** Quantification of concordant (grey) and discordant (white) links within allele-specific and balanced regulatory links at various distances (right panel). Balanced links were classified as concordant or discordant according to the sign of the non-significant log2-fold-change. The plot shows the combined data from all five individuals. **H** Schematic of the population-derived GTEx eQTL data (top panel). Quantification of variants within putative regulatory links overlapping with eQTL data from LCLs (bottom panel). **I** Relationship between the effects from personal genomics and GTEx slopes for allele-specific (top panel) and balanced (bottom panel) regulatory links. Each data point represents a variant-gene regulatory link that is also an eQTL in the GTEx dataset for LCLs. The odds ratios are calculated using Fisher’s exact test. Concordant and discordant links are shown in grey and white, respectively. The plot shows the combined data from all five individuals.

Genetic variants driving allele-specific imbalance of regulatory elements activity and gene expression have been previously identified at the population level in a number of previous studies^48–52,69–72^. To study allele-specific regulatory elements and genes at the single individual level, we generated chromatin accessibility and gene expression profiles with an assay for transposase-accessible chromatin using sequencing (ATAC-seq) and transient transcriptome sequencing (TT-seq), respectively. Using the obtained phased variants, we developed a custom, personal genome-based computational pipeline that enabled us to map, distinguish and quantify signals from each haplotype (Fig. 2A and Supplementary Fig. 3A,B; see “Methods”). Unlike standard, reference genome-based approaches, this method reduces mapping biases by accounting for individual-specific SNPs and indels, while also enabling the aggregation of data across multiple variants for a more accurate evaluation of allele-specific changes^56,73^.

To assess and compare inter-individual and inter-allele variation, we performed principal component analysis on the chromatin accessibility and gene expression data. Our results show that variability between individuals far exceeded variability within individuals (Supplementary Fig. 3C). The inter-individual differences most likely result from genetic differences but may also, in part, be a consequence of clonal variability between cell lines^74,75^. Next, we focused on quantifying chromatin accessibility and gene expression. Between 34.4% and 55.7% of the annotated peaks and between 78.4% and 89.4% of the expressed genes contained at least one phased heterozygous variant (Supplementary Fig. 3D). The median allelic read coverage for these peaks and genes ranged between 7-15 and 31-68 reads per replicate, respectively, making the majority of them informative for allele-specific analysis^76,77^ (Supplementary Fig. 3E,F). Statistical analysis revealed hundreds of allele-specific open chromatin peaks and genes per individual, as opposed to the thousands observed between samples (log2-fold-change>1, FDR < 0.05 and 0.1 for ATAC-seq and TT-seq, respectively, Wald test; Figs. 2B, C, Supplementary Fig. 4A and Supplementary Data 3). The majority of allele-specific events were unique to one individual, with only a minority shared across all samples (Supplementary Fig. 4B), consistent with previous findings^78^.

Next, we sought to validate the annotated allele-specific open chromatin peaks and genes. As expected, a large proportion of allele-specific events were observed exclusively on the X chromosome in female samples due to the epigenetic silencing of the inactive X chromosome (Supplementary Fig. 4C). The analysis of publicly available ChIP-seq data^79^ showed that allele-specific open chromatin peaks were accompanied by a similar imbalance of histone modifications, providing complementary evidence of differential activity (Supplementary Fig. 4D). In addition, these regions exhibited enrichment of previously identified chromatin accessibility QTL^80^ (Supplementary Fig. 4E). The identified allele-specific genes showed enrichment for imprinted genes, which have parent-of-origin-specific expression^81,82^ (Supplementary Fig. 4F). Furthermore, since LCLs are derived from B cells, we found that immune-related genes exhibited skewed allelic expression through the allelic exclusion mechanism^83,84^ (Supplementary Fig. 4G). Genes previously identified as having random allelic expression^85^ were also enriched in our set of allele-specific genes (Supplementary Fig. 4H). Together, these findings highlight the robustness of our method in identifying genes that display allele-specific expression.

### Allele-specific regulatory links show proximity and concordance between accessibility and transcription

Since both alleles of the diploid genome are exposed to the same concentrations of transcription factors and chromatin regulators, we reasoned that apart from epigenetic mechanisms discussed above, in most cases, differentially active regulatory elements are a consequence of underlying genetic variation. We hypothesised that, by linking allele-specific accessibility to gene expression in single individuals, we can predict functional non-coding genetic variants with increased precision. To this end, we constructed putative regulatory links by connecting allele-specific open chromatin peaks and genes over a genomic distance of one megabase (Fig. 2C, D, Supplementary Fig. 5A,B and Supplementary Data 4). As a control set, we created balanced links by connecting balanced open chromatin and genes. Since allele-specific expression of the X chromosome, imprinted, and immune genes is mediated by epigenetic silencing and allelic exclusion mechanisms, unrelated to the impact of genetic variation on regulatory element activity, we removed them from our analyses (Supplementary Fig. 5A). Genes from the most polymorphic region of the human genome, the major histocompatibility complex, were also excluded to avoid previously reported biases^86,87^ (Supplementary Fig. 5A).

To compare our putative regulatory links with interactions derived from high-resolution chromosome conformation capture assays, we overlapped them with chromatin loops identified in Hi-C^63^, promoter capture Hi-C^88^, H3K27ac HiChIP^89^, and Micro-C^90^ from the NA12878 individual. Of the allele-specific and balanced regulatory links, 16.4% and 22.2% respectively overlapped with chromatin loops from at least one assay (Supplementary Fig. 5C). While overlap with a chromatin loop can be informative, it is not a prerequisite for a regulatory element to be functionally relevant to a gene^91–94^. Conversely, topologically associating domains (TADs) offer a more flexible approach to identifying relevant enhancers and promoters^95,96^. Consistent with this, the majority of regulatory links within chromatin loops were found to be within the same TAD (Supplementary Fig. 5D). Therefore, we decided to use TAD boundaries as a proxy for the limits of functional interactions, requiring the putative regulatory links to additionally be located within the same TAD (Fig. 2D and Supplementary Fig. 5E).

Accessible chromatin regions are associated with gene activity^97^. Using our set of putative regulatory links constrained by both genomic distance and TAD boundaries, we were able to establish the distances over which regulatory elements can exert their activity. Compared to a set of balanced links, allele-specific links showed a shift towards short-range distances between open chromatin and expression, particularly at distances below 50 kb (Fig. 2E and Supplementary Fig. 5F). The use of TAD boundaries as an additional constraint primarily reduced the number of long-range links, many of which are composed of peaks and genes involved in multiple links (Fig. 2E, Supplementary Fig. 5A, B, F). At distances below 50 kb, nearly all allele-specific regulatory links demonstrated concordance between accessibility and transcription, indicating that they are active on the same haplotype (Fig. 2F, G and Supplementary Fig. 5G). As the distance increases, the proportion of discordant regulatory links, which connect allele-specific accessibility and expression on opposite alleles, becomes more abundant. While some of these discordant links may represent repressive mechanisms, their increased prevalence at longer distances suggests that they are more likely to reflect spurious associations of regulatory elements with non-target genes. Our results therefore indicate that a substantial proportion of active regulatory elements is located on the same allele in proximity to active genes, consistent with earlier population-based studies^91,98–101^.

### Links from personal genomes are consistent with population-level eQTL associations

To compare our personal genomics approach with population-derived associations, we integrated regulatory links with eQTL data from the Genotype-Tissue Expression (GTEx) project^7^. For this comparison, we used the variants underlying the peaks involved in allele-specific and balanced links (Supplementary Fig. 6A and Supplementary Data 4), overlapping them with the GTEx variant-gene associations. Between 4.2% and 11.4% of allele-specific links were identified as significant eQTLs from LCLs, which was considerably higher than 0.7% to 1.4% of balanced links (Fig. 2H). Similar results were obtained when our regulatory links were compared with eQTLs from other tissues (Supplementary Fig. 6B). Notably, the NA12878 individual consistently demonstrated the largest proportion of regulatory links found in GTEx eQTLs for any tissue, while the other four individuals demonstrated lower proportions with similar distribution. This difference in the degree of overlap can be attributed to the population representation within the GTEx database, in which 85.3% of donors are of European ancestry^7^.

To further investigate the agreement between personal genomics and population-based links, we examined the concordance of overlapping associations. We compared the eQTL slope from GTEx with the measured effects on chromatin accessibility and gene expression. A high degree of agreement for these associations in LCLs was observed for allele-specific (OR = 235.1), but not balanced links (OR = 4.1) and was reproducible across all five individuals (Fig. 2I and Supplementary Fig. 6C). The eQTL slopes from all other tissues also showed concordance with our data, suggesting that our approach aligns well with large-scale population-derived data, particularly from the LCLs (Supplementary Fig. 6D).

In addition to the eQTLs identified by GTEx, the regulatory links derived from personal genomes contain many low-frequency variants with minor allele frequency (MAF) < 0.05, as well as variants that were not genotyped in the GTEx cohort (Fig. 2H). The effects of these variants are difficult to detect at the population level due to their rareness, yet our analyses suggest they can influence gene expression, underscoring the utility of a personal genomics approach. Overall, these results demonstrate that allele-specific regulatory links from personal genomes are enriched for and consistent with associations identified in large-scale population studies.

### Deep learning identifies LCL-specific regulatory grammar from chromatin accessibility

Deep learning models are becoming an increasingly common strategy for identifying cell-type-specific regulatory grammar de novo from DNA sequence^21–31,102–107^. By interpreting features learned by the models, TF binding motifs underlying regulatory elements can be deduced^108^. These motifs can then be used to pinpoint the impact of genetic variants and explain allele-specific activity of regulatory elements^23,25,47,73,105,109,110^.

**Fig. 3:**
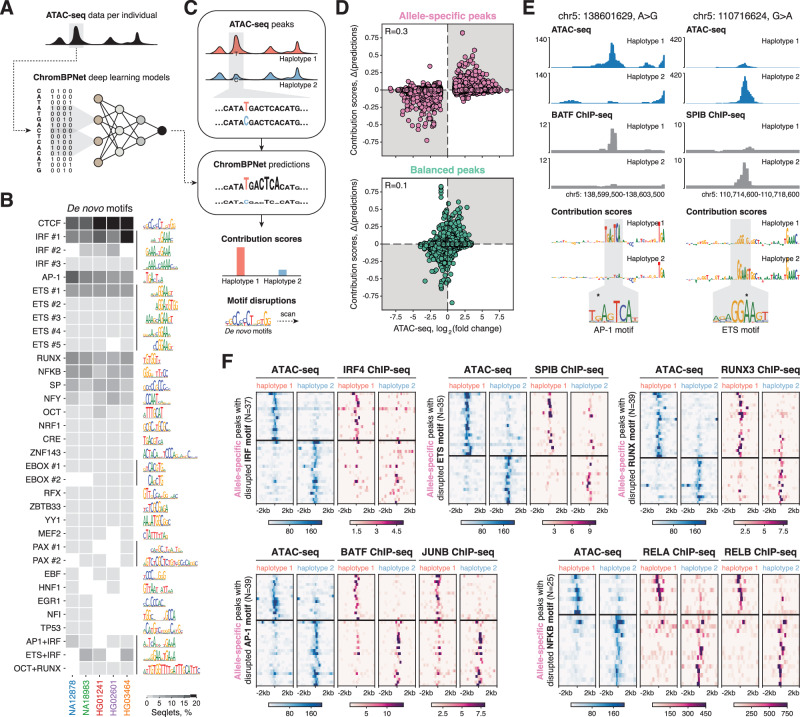
ChromBPNet reveals variants that disrupt transcription factor binding within allele-specific open chromatin regions. **A** Schematic of the individual-specific ChromBPNet deep learning models training using the ATAC-seq data in the open chromatin peaks and the underlying DNA sequence. **B** Clusters of de novo transcription factor binding motifs derived from the trained ChromBPNet models using TF-MoDISco. Each tile represents the relative fraction of motif cluster seqlets extracted by TF-MoDISco for that individual. Empty tiles indicate that the motif cluster was not detected in that particular individual. The motifs are grouped by TF and sorted by abundance. **C** Schematic of the workflow to gauge the impact of genetic variants on chromatin accessibility. DNA sequences from both alleles underlying ATAC-seq peaks were used to predict contribution scores with individual-specific ChromBPNet models. The difference in the contribution scores within a 50 bp window surrounding the variants was then calculated, after which the adjacent DNA sequences were scanned for motif matches using the de novo motif clusters derived by TF-MoDISco. **D** Scatterplot showing the relationship between measured chromatin accessibility differences and predicted contribution score differences between alleles for variants located in allele-specific (top panel) and balanced (bottom panel) open chromatin peaks. The Pearson correlation coefficient is indicated in the top left corner. The plot shows the combined data from all five individuals. **E** Examples of predicted AP-1 (left panel) and ETS (right panel) transcription factor motif disruption within allele-specific chromatin accessibility peaks. Motif disruption was annotated using predicted ChromBPNet contribution scores and validated using publicly available ChIP-seq data from the NA12878 individual. The position of the variants within the motifs are indicated by the asterisks. **F** Tornado plots showing the ATAC-seq and ChIP-seq signals at the allele-specific peaks with disrupted IRF, AP1, ETS, NFKB or RUNX motifs. The regions are sorted by predicted contribution score differences.

To decipher the TF binding motifs that drive chromatin accessibility in LCLs, we trained the ChromBPNet models^23^ on the generated ATAC-seq data (Fig. 3A). To be able to interpret the allele-specific peaks in an unbiased manner later on, we eliminated any peaks demonstrating allelic imbalance prior to the training (FDR < 0.1). We then trained and evaluated the performance of the ChromBPNet models for each individual using the reference human genome, employing a five-fold chromosome-holdout cross-validation scheme^23,109,110^. We obtained a stable Pearson correlation between total observed and predicted counts and a stable Jensen-Shannon distance between observed and predicted profile shapes across folds (Supplementary Fig. 7A). Visual inspection of the base-pair profile contribution scores for each nucleotide within representative peaks indicated that each model had consistently learned predictive TF motifs for both promoter and enhancer regions (Supplementary Fig. 7B).

Using the TF-MODISCO motif discovery tool, we extracted sequences with high contribution scores (seqlets) corresponding to TF motifs for each individual model. To reduce redundancy and retain motifs consistently identified across multiple individuals, we adapted the published framework to perform motif clustering^111^ (see “Methods”), and then matched the resulting clusters to known TF motif instances from the JASPAR database^112,113^ (Fig. 3B). This systematic annotation revealed that IRF, AP1 and ETS motifs of the key B-cell-related transcription factors were highly abundant among the extracted seqlets, indicating that they frequently occur within open chromatin regions^114–116^. In addition, less prevalent motifs, such as RUNX, NFKB, SP, NFY, and OCT, were also found to be predictive of chromatin accessibility in LCLs^117^. Finally, motifs of general TFs such as CTCF, ZNF143, and YY1 were also identified^118,119^. Taken together, these results show that ChromBPNet successfully identified the key motif syntax of transcription factors that drive the LCL gene regulatory network de novo.

### Differences in chromatin accessibility between alleles are reflected in predicted variant effects

Our reconstructed whole-chromosome haplotypes provide an ideal, internally controlled setup for evaluating the performance of ChromBPNet models when assessing the impact of genetic variants using allele-specific data from the endogenous genomic context. Using the trained ChromBPNet models and the personal genomes for each individual, we predicted the contribution scores of the DNA sequences underlying ATAC-seq peaks on both alleles (Fig. 3C). We then calculated the difference between contribution scores within 50 bp windows around each variant to estimate its local effect (**see Methods**). Comparing the predictions with experimentally measured allele-specific chromatin accessibility, we observed a moderate correlation (Pearson *r* = 0.3) between the observed chromatin accessibility changes and the predicted contribution score differences for allele-specific open chromatin peaks, but not balanced links (Pearson *r* = 0.1), consistent across individuals (Fig. 3D and Supplementary Fig. 8A). While the qualitative concordance between the predicted and measured variant effects was high, the effect size appears to be more challenging for these models to predict, as for many variants the predicted contribution score difference was close to zero. Using various cutoffs for contribution score difference, we estimated that ~35% of allele-specific open chromatin peaks could be reliably explained by the model (cutoff = 0.02; Supplementary Fig. 8B). This suggests that, while ChromBPNet models already possess sufficient explanatory power to partially account for the observed allele-specific differences, they do not yet fully capture the complexity of the data.

### Allele-specific accessibility can be explained by variants disrupting transcription factor binding

To pinpoint the mechanisms by which genetic variants affect the accessibility of regulatory elements, we scanned the predicted ChromBPNet contribution scores for both haplotypes using the identified LCL-specific TF motif syntax. For each variant, we calculated the correlation between the TF motif and contribution scores and then developed a set of rules to identify motif disruptions by variants (Fig. 3E, Supplementary Data 5; see “Methods”). Using this approach, we annotated between 153 and 392 peaks with disrupted motifs, accounting for between 17.3% and 27.3% of allele-specific peaks per individual. The majority of annotated TF motif disruptions comprised LCL-specific motifs, such as IRF, AP1, and ETS, as opposed to general transcription factors, such as ZNF143 and CTCF, whose motifs were rarely mutated, likely due to the fact that they regulate housekeeping genes^39,105,120,121^ (Supplementary Fig. 9A).

We subsequently investigated whether motif disruptions lead to variation in TF binding between alleles. To study this, we used publicly available ChIP-seq data from the NA12878 individual^122,123^ for TFs which had at least 25 identified motif disruptions (IRF, AP1, ETS, NFKB and RUNX; Supplementary Data 1 and 5). Quantifying the chromatin occupancy of these TFs at allele-specific open chromatin peaks that overlapped the disrupted motifs revealed clear differences in ChIP-seq signal between the alleles, consistent with an imbalance in chromatin accessibility (Fig. 3E, F). We then confirmed that the resulting difference in TF occupancy was due to the motif disruptions predicted by ChromBPNet, rather than a potential non-specific ChIP-seq bias for the accessible allele. To this end, we created a control set of allele-specific open chromatin peaks containing the intact binding motifs of the analysed TFs on both alleles. No strong systematic TF binding occupancy imbalance between haplotypes was found by quantifying the signals for this control set of allele-specific open chromatin peaks (Supplementary Fig. 9B). This enabled us to confidently attribute the observed differences in TF binding signal to predicted motif disruptions, offering a potential explanation for the mechanisms behind the observed allele-specific chromatin accessibility. These results show that deep learning can facilitate the interpretation of identified allele-specific regions by annotating variants that disrupt TF motifs.

Interestingly, many variants within the balanced accessibility peaks were predicted to exhibit substantial differences in contribution scores (Fig. 3D and Supplementary Fig. 8A). We set out to understand whether these discrepancies reflect the disruptions of motifs and TF binding that do not affect chromatin accessibility or can be attributed to the limitations in the predictive models. To investigate this, we conducted analogous analyses for balanced peaks using the same TF chromatin occupancy data from the NA12878 individual. Quantifying ChIP-seq signals over the balanced peaks with disrupted motifs revealed that, despite the motif disruption, most variants had no effect on TF binding between alleles (Supplementary Fig. 10A). Focusing on representative balanced peaks in the *PPIF* promoter and the *SDCCAG8* enhancer, we observed that the analysed variants were located within CTCF and SPI1 binding motifs, respectively, and were predicted to be disruptive (Supplementary Fig. 10B). However, the presence of variants had little effect on the measured CTCF and SPI1 binding^79,122^, contrasting with model predictions. Furthermore, for the *PPIF* gene, the variant located within the CTCF motif in the promoter did not affect its expression (log2-fold-change = 0.15, FDR > 0.99, Wald test; Supplementary Fig. 10B), suggesting that this variant does not manifest a functional difference. These results demonstrate that, although deep learning approaches have advanced variant effect prediction, their outputs must be interpreted with caution, particularly in the absence of supporting experimental validation. Consequently, experimental data still serves as the gold standard for interpreting regulatory genetic variation and remains fundamental for benchmarking and refining predictive models.

### Personal genomics combined with deep learning identifies known and putative causal variants

**Fig. 4:**
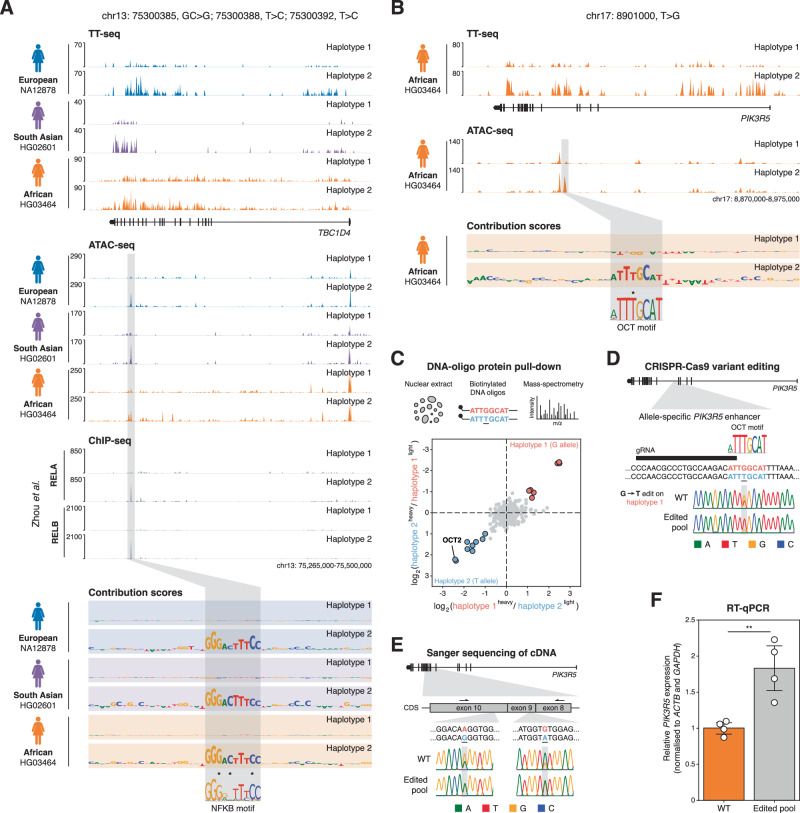
Personal genomics and deep learning detect known and novel variants modulating gene expression. **A** Haplotype-resolved ATAC-seq, TT-seq, ChIP-seq, and predicted ChromBPNet contribution scores for the *TBC1D4* gene in the NA12878, HG02601, and HG03464 individuals. The grey rectangle indicates the position of the allele-specific open chromatin peak containing the NFKB motif. The positions of the variants within the motif are indicated by asterisks. **B** Haplotype-resolved ATAC-seq, TT-seq, and predicted ChromBPNet contribution scores for the *PIK3R5* gene in the HG03464 individual. The grey rectangle indicates the position of the identified allele-specific open chromatin peak containing an OCT motif, which is disrupted by the presence of the rare rs545467951 variant. The position of the variant within the motif is indicated by the asterisk. **C** Schematic of the DNA oligo protein pull-downs with mass spectrometry (top panel). Mass spectrometry was performed with heavy labelling of proteins bound to the G allele and light labelling of proteins bound to the T allele, as well as with a reversed labelling orientation. Quantification of log2-fold-changes is shown in the scatter plot (bottom panel). **D** Schematic of the CRISPR-Cas9-mediated genome editing to convert the G allele to the T allele within haplotype 1 of the allele-specific distal enhancer of the HG03464 LCLs (top). Sanger sequencing tracks of the genomic DNA are shown for the wild-type and the edited pool (bottom). **E** Schematic of the *PIK3R5* cDNA quantification in HG03464 LCLs using Sanger sequencing (top). Sequencing tracks of the *PIK3R5* coding DNA are shown for the wild-type and the edited pool (bottom). Relative quantification of the RNA expressed from each haplotype is shown on the right. **F** RT-qPCR quantification of the mRNA level of *PIK3R5* in HG03464 LCLs. Fold change in the expression of the *PIK3R5* gene in the edited pool relative to the wild-type, normalised to the housekeeping genes *ACTB* and *GAPDH*, is shown. Each data point represents a technical replicate (*N* = 4), the bar plot represents a mean value, and the error bars indicate 95% confidence interval of the mean. Statistical significance was assessed using a two-sided *T* test (*p*-value = 0.0029, **p < 0.01).

Next, we integrated the ChromBPNet predictions with identified allele-specific regulatory links. We found that 14.6–19.9% of regulatory links could be explained by the predicted TF motif disruptions, of which 51.9–73.9% comprised concordant links (Supplementary Fig. 11A). Notably, both short-range (< 50 kb) and long-range (> 50 kb) links are identified in our survey (Supplementary Fig. 11B). For the explained concordant regulatory links, we next compared the predicted transcription factors behind allele-specific gene expression with existing literature on causal variant-gene associations. Several of the associations in our personal genomics regulatory links had previously been reported in large-scale studies examining variants that affect chromatin accessibility, gene expression, and the 3D genome^97,100,124–126^. These include, for example, variants causing allele-specific expression of *TBC1D4* in NA12878, HG02601, and HG03464 individuals, as well as *SLFN5* in NA18983, HG01241, and HG02601 individuals by affecting their intronic enhancers (181.8 kb and 1.5 kb from the promoter, respectively). Using our framework, we have independently identified the previously described disruptions of NFKB and IRF motifs^97,124^, which were validated by the binding of the corresponding REL and IRF4 transcription factors^122,127^ (Fig. 4A and Supplementary Fig. 11C).

Alongside the known and validated mechanisms behind variant-gene associations, we identified several novel variants that are likely to be causal for allele-specific expression. For example, we found an allele-specific regulatory element upstream of *CAV2*, encoding the caveolin-2 protein, in the NA12878 and HG03464 individuals (1.2 kb from the promoter, Supplementary Fig. 11D). This element contained a disrupted RUNX motif, resulting in allele-specific RUNX3 binding. In addition, *PAX8-AS1*, an antisense transcript of the gene encoding the PAX8 transcription factor, exhibited allele-specific expression in the NA12878 and HG01241 individuals (24.4 kb from the promoter, Supplementary Fig. 11E). Its promoter-proximal regulatory element contained a disrupted ETS motif, resulting in allele-specific ELF1 binding. These examples illustrate that our framework, which is entirely based on personal genomics and does not require additional samples for statistical testing, is capable of identifying variant-gene associations and their putative mechanisms.

### A novel rare variant modulates the expression of *PIK3R5* gene by disrupting an OCT2 binding motif

Lastly, we examined our regulatory links for novel variants that had not previously been recognised for their regulatory potential. One example that stood out was the *PIK3R5* gene, which encodes phosphoinositide 3-kinase regulatory subunit 5. This subunit is part of the PI3K kinase, which is essential for signal transduction and is one of the most frequently activated signalling pathways in cancer^128,129^. In the HG03464 individual, *PIK3R5* is expressed in an allele-specific manner and contains an intronic enhancer located in the same allele, 64.7 kb from the promoter. ChromBPNet contribution scores predicted that the rare African-specific T > G variant (rs545467951, MAF = 0.0034 in African population^130,131^) within the enhancer disrupts an OCT motif, likely recognised by the B-cell-specific transcription factor OCT2^132^ (Fig. 4B). Using publicly available ChIP-seq data, we confirmed that this enhancer is occupied by OCT2 in the NA12878 individual^122^ (Supplementary Fig. 12A). Both alleles of this individual contain an intact OCT motif and exhibit similar *PIK3R5* gene expression. In addition, recently published high-resolution Micro-C data for NA12878 revealed that the OCT2-occupied enhancer interacts with the *PIK3R5* promoter^90^ (Supplementary Fig. 12A), providing further support for the functional role of this enhancer in *PIK3R5* regulation.

We next proceeded to validate the role of OCT2 in regulating *PIK3R5* expression via the identified distal enhancer. To this end, we performed DNA affinity purifications coupled to quantitative mass spectrometry using HG03464 nuclear extract and oligonucleotides containing an OCT motif, with either a G (disrupted) or a T (intact) nucleotide serving as baits^133^. This experiment confirmed that OCT2 preferentially binds to the T allele compared to the G allele (Fig. 4C). To establish the causal relationship between this variant and *PIK3R5* gene expression, we used CRISPR/Cas9 to restore the disrupted OCT motif by introducing a G-to-T edit in the disrupted haplotype of the HG03464 individual^126,134^ (83.1% HDR-mediated editing efficiency; Fig. 4D and Supplementary Fig. 12B,C). We observed a significant increase in *PIK3R5* expression in the edited pool compared to the wild-type HG03464 LCLs (1.83 fold-change, *p*-value = 0.0029, two-sided *T* test), explained by an increase in transcripts produced from the edited haplotype (Figs. 4E, F). These results validate that the rare non-coding T > G variant regulates *PIK3R5* gene expression by disrupting the OCT2 binding site within an intronic regulatory element. Together, these findings demonstrate the value of our personal genomics framework, enabling us to identify novel causal non-coding genetic variants, affected target genes, and the regulatory mechanisms they disrupt.

## Discussion

By combining whole-chromosome haplotyping with multiomics assays, we associated non-coding variants from open chromatin regions with gene expression in individual human genomes in an allele-specific manner. Using deep learning models, we investigated the principles and mechanisms behind allele-specific gene regulation by annotating disrupted cell-type-specific transcription factor binding motifs. The power of such an approach is demonstrated by the discovery of a variant that modulates *PIK3R5* expression by disrupting an OCT2 binding motif in a distal enhancer. Altogether, we establish a framework for interpreting the functional impact of genetic variation in the context of personal genomics.

In contrast to population-based GWAS and eQTL studies, which require large cohorts to determine variant-gene associations, our approach derives functional insights from data of a single individual. This framework can complement population-based analyses by prioritising the associated variants and suggesting their potential mechanisms of action. Furthermore, it accelerates the annotation of rare variants that fall below statistical thresholds in cohort studies, a particularly relevant feature for the clinical interpretation of disease-driving variants in patient genomes. Thus, our framework connects personal genomics with the functional interpretation of genetic variation, paving the way for the validation of prioritised findings.

As the presented framework is a proof of concept, we started with previously obtained phased variants and progressed to a functional analysis of allele-specific expression and accessibility. It is important to note that, since all cells within an individual share the same genetic code, only one round of haplotyping or assembly is required to later evaluate the functional impact of genetic variants in any cell type. In this study, we used reference-based whole-chromosome haplotype reconstruction with 10X linked-reads and Hi-C data. Although several other linked-read technologies are available^66,67^, using long-read sequencing technologies from Pacific Biosciences (PacBio) and Oxford Nanopore Technologies (ONT) might be more relevant in a clinical setting^135–138^. These technologies would additionally enable assessment of genetic variation beyond heterozygous SNPs and indels, including complex structural variants, repeat expansions and transposable element insertions, all of which may be clinically relevant^139,140^. Long reads can then be combined with Hi-C to provide whole-chromosome phasing, or used directly for de novo variant identification and haplotype assembly^60^. Together, these considerations enhance the potential of haplotype-resolved genomics to bridge the gap between individual genetic variation and cell-type-specific regulatory landscapes.

Here, we demonstrate the potential of deep learning models to explain allele-specific regulatory elements through identifying disrupted transcription factor binding sites. Notably, these annotations are derived without prior knowledge of the LCL-specific motif syntax, as the models infer it directly from the chromatin accessibility data. This can be especially valuable for investigating variant effects in rare cell types or clinical samples, where gene regulatory networks are poorly characterised. However, while current models are good at predicting the direction of the variant effect, the predictions of the effect size are in many cases, still inconsistent with the observations. This means we are likely missing causal variants outside of the TF motifs or in regions distal to accessibility peak summits. Moreover, as we demonstrate here, the models may predict variant-driven differences which are not supported by the experimental data, emphasising the need for careful evaluation of their output. These issues could be potentially overcome by fine-tuning the existing models (e.g., by using allelic signals or complementary regulatory features), training models on larger genomic windows, and integrating predictions generated by multiple models.

Integrating multiomics data with deep learning is a promising approach to improving our understanding of variants in both common and rare phenotypes. As computational models are becoming more sophisticated^141,142^ and molecular assays that measure multiple data modalities simultaneously are seeing increasing adoption^143–147^, our ability to link genetic variation to functional consequences and uncover regulatory mechanisms will improve significantly. Together, these approaches promise to bridge the gap between variant discovery and mechanistic insight, accelerating the translation of genetic associations into biological understanding and clinical impact.

## Methods

### Cell lines

The following lymphoblastoid cell lines were obtained from the NHGRI Sample Repository for Human Genetic Research at the Coriell Institute for Medical Research: GM12878, GM18983, HG01241, HG02601, and HG03464 (referred to here as NA12878, NA18983, HG01241, HG02601, and HG03464 individuals). LCLs were cultured in suspension in 80% RPMI-1640 media (Gibco, Cat. #11875093) supplemented with 20% foetal bovine serum, 2 mM L-glutamine, and 1X penicillin/streptomycin. Cells were routinely tested for Mycoplasma (Lonza, Cat. #LT07-318).

### Samples selection

To select the samples with the largest genetic divergence, we used variant data from the 1000 Genomes Project^1^ (release date: 2013-02-05). Individual heterozygosity per sample was calculated using VCFtools with the “--het” flag and then normalised by the mean heterozygosity within each superpopulation. Variants in strong linkage disequilibrium were removed using PLINK, ensuring a set of relatively independent variants (r2 ≤ 0.2) for dimensionality reduction.

Dimensionality reduction was performed on the resulting genotypes × samples matrix using principal component analysis, implemented via the “snpgdsPCA” function from the SNPRelate R package^148^. The top three principal components were retained and used as input for t-distributed stochastic neighbour embedding (t-SNE) to obtain two-dimensional sample coordinates. Euclidean distances between samples in the t-SNE space were computed, followed by hierarchical clustering using complete linkage and an arbitrary cut-off point to produce clusters.

Then, sets of five samples were assembled such that each sample came from a distinct superpopulation, belonged to a separate cluster and exhibited heterozygosity above the 90th percentile within its superpopulation. The set (i) containing commonly used NA12878 and (ii) with the highest total normalised heterozygosity score was selected from these combinations and comprised NA12878, NA18983, HG01241, HG02601 and HG03464 individuals.

The map with geographical locations of the selected five samples was created using Matplotlib v3.5.2 Basemap library with cylindrical equidistant projection and coarse resolution^149^. The coordinates of the samples are based on the locations reported by the 1000 Genomes Project^1^.

### Linked-reads

Linked-reads data generation for the NA18983, HG01241, HG02601, and HG03464 LCLs was outsourced to NGX Bio. For each sample, 3 million cells were harvested in PBS, and the frozen cell pellets were sent to NGX Bio for processing. The resulting high molecular weight DNA was extracted using the MagAttract HMW DNA Extraction Kit (Qiagen, Cat. #67563). The quality of the DNA was assessed using the TapeStation system (Agilent Technologies). Libraries were prepared using the 10X Genomics GemCode platform with reagents from 10X Genomics and the KAPA Hyper Prep Kit (Roche, Cat. #07962363001). Each library was sequenced on a single lane of the Illumina HiSeq X Ten sequencing platform, generating 150 bp paired-end reads.

### Publicly available linked-reads data

NA12878 data from the Genome in a Bottle Consortium was used^62^. NA20509 data from the Illumina Polaris PGx 10X Cohort was used (https://github.com/Illumina/Polaris). HG03486 data from the Human Pangenome Reference Consortium was used^150^. NA19238, NA19239, NA19240 (Yoruba Trio), HG00512, HG00513, HG00514 (Han Chinese Trio), HG00731, HG00732, HG00733 (Puerto Rican Trio) data from the Human Genome Structural Variation Consortium was used^61^. The accession numbers and links to the datasets are listed in Supplementary Data 1.

### Hi-C

Hi-C data for the NA18983, HG01241, HG02601, and HG03464 LCLs was generated as previously described with minor modifications^63,151^. For each template, 10 million cells were harvested and crosslinked using 2% formaldehyde. Crosslinked DNA was digested in the nucleus using MboI restriction enzyme (NEB, Cat. #E2621L), and biotinylated nucleotides were incorporated at the restriction overhangs and joined by blunt-end ligation. The ligated DNA was enriched in a streptavidin pull-down. Hi-C libraries were prepared using a standard end-repair and A-tailing method. Libraries were sequenced by the Hartwig Medical Foundation on the Illumina HiSeq X Ten sequencing platform, generating paired-end 150 bp reads.

### Publicly available Hi-C data

NA12878 data generated using the DpnII restriction enzyme was used^63^. NA20509 data generated by the Human Genome Structural Variation Consortium was used^68^. HG03486 data generated by the Human Pangenome Reference Consortium was used^150^. NA19238, NA19239, NA19240 (Yoruba Trio), HG00512, HG00513, HG00514 (Han Chinese Trio), HG00731, HG00732, HG00733 (Puerto Rican Trio) data generated using the HindIII restriction enzyme was used^152^. The accession numbers and links to the datasets are listed in Supplementary Data 1.

### ATAC-seq

Libraries for the ATAC-seq were prepared as previously described in three biological replicates^119^. A total of 50,000 cells were harvested and subjected to lysis using a 2X lysis buffer. Next, cells were pelleted and incubated with 2X TD buffer and 2 μL of transposon mix. Tagmentation was performed using in-house-generated Tn5 transposase^153^ for 1 h at 37 degrees with shaking. After tagmentation, samples were cleaned by adding 9 μl of clean-up buffer. DNA was purified via normal phase purification using 2X AMPure XP beads (Beckman Coulter, Cat. #A63881). Following this, the first PCR amplification was conducted using KAPA HiFi HotStart PCR ReadyMix (Roche, Cat. #KK2602) using P5 and P7 indexed primers. Fragments ranging from 200 to 700 bp were purified via reverse phase purification utilising 0.55X AMPure XP beads (Beckman Coulter, Cat. #A63881) followed by purification with QIAquick PCR Purification Kit (Qiagen, Cat. #28104). The second PCR amplification was then conducted under the same conditions. The quality of the DNA was assessed through Bioanalyzer High Sensitivity DNA analysis (Agilent). Libraries were sequenced by Novogene on the Illumina NovaSeq 6000, generating paired-end 150 bp reads.

### TT-seq

Libraries for TT_chem_-seq were prepared following a published protocol in two biological replicates^154^. For each sample, 8 million lymphoblastoid cells were labelled for 10 minutes with 2 mM of the uridine analogue 4-thiouridine (Sigma-Aldrich, Cat. #T4509). Total RNA was then isolated and fragmented. The 4sU-biotin labelled RNA was enriched using µMacs Streptavidin Kit (Miltenyi, Cat. ##130-074-101). Libraries were prepared using KAPA RNA HyperPrep (Roche, Cat. #KK8540) and KAPA Dual-Indexed Adaptor Kits (Roche, Cat. #KR1736) using dual indexing adaptors. Libraries were sequenced by the NKI Genomics Core Facility on the Illumina NextSeq 550, generating paired-end 150 bp reads.

### Whole-chromosome haplotypes phasing

#### Variants data processing

Genetic variation data called for the LCLs was obtained from the 1000 Genomes Project^130^. We generated consensus variant sets of SNPs and INDELs for each VCF as described previously^155^. For each sample, we removed structural variants and kept only the variants with at least one alternative allele using BCFtools v1.9^156^ and SnpSift v4.3p^157^. First, the variants were normalised and left-aligned, and multiallelic sites were split into multiple rows with BCFtools norm v1.9 with “-m -any” flag. Second, complex variants were decomposed using vcfallelicprimitives from vcflib v1.0.3^158^, then sorted and unified using vt v2015.11.10^159^. Any potential multiallelic sites that occurred during the procedure were merged back using BCFtools norm v1.9 with “-m + any” flag^156^. From this set, only biallelic sites were retained using BCFtools view v1.9 with “-m2 -M2” flags^156^. Lastly, overlapping variants were removed using the vt remove_overlap and SnpSift “filter” functions. The statistics of the resulting variants per sample were calculated using the “vcfstats” function from RTG Tools v3.12.1 (https://github.com/RealTimeGenomics/rtg-tools).

#### 10X linked-reads data processing

The 10X linked-reads data were mapped to the hg38-based reference genome (refdata-GRCh38-2.1.0) provided by 10X Genomics using the Long Ranger pipeline v2.2.2 with the “--precalled” parameter to specify a VCF file containing variant data from the 1000 Genomes Project. Segmental duplications, reference gaps, unplaced contigs, regions with assembly issues and highly polymorphic sites were excluded from the analysis using annotation files from the reference genome provided by 10X Genomics. If only the BAM file was available for publicly accessible datasets, it was first converted to FASTQ using bamtofastq v1.4.1 (https://github.com/10XGenomics/bamtofastq), and then mapped as described above. Duplicate reads were filtered out using the Picard v2.27.4 “MarkDuplicates” and only the reads with a barcode were retained using SAMtools v1.15^160^ with “-d BX” flag.

#### Publicly available stLFR and TELL-seq data processing

The TELL-seq data for NA12878 was processed based on the TELL-Sort pipeline by Universal Sequencing and the pipeline from Chen et al.^66^. The barcodes were added to the read names using “add_bx” from TELLseq v0.1.3. The resulting reads were mapped to the hg38-based reference genome from 10X Genomics with bwa mem v0.7.17-r1188^161^ with the “-C” parameter to transfer the barcode information to the BAM output. The resulting BAM file was sorted using SAMtools v1.6^160^, and duplicate reads were filtered out using the Picard v2.27.4 “MarkDuplicates” with VALIDATION_STRINGENCY set to “SILENT”.

The stLFR data for NA12878 was processed based on the pipeline from Wang et al.^67^. The reads were mapped to the hg38-based reference genome from 10X Genomics with bwa mem v0.7.17-r1188^161^ with “-R” parameter set to “@RG\tID:L0\tSM:L0\tPL:COMPLETE”. The resulting BAM file was sorted using SAMtools v1.6^160^, and duplicate reads were filtered out using the Picard v2.27.4 “MarkDuplicates” with VALIDATION_STRINGENCY set to “SILENT” and a custom READ_NAME_REGEX parameter. Lastly, reads with unassigned barcodes were then filtered out, after which the resulting BAM file was modified using AWK to add a BX field with barcode information from the read name.

#### Hi-C data processing

For whole-chromosome haplotyping, the Hi-C data was mapped to the hg38-based reference genome from 10X Genomics with bwa mem v0.7.17-r1188^161^ with “-5SPM” flags, in accordance with the recommendations for the HapCUT2 pipeline^162^. Duplicate reads were filtered out using the Picard v2.27.4 “MarkDuplicates”. The resulting BAM file was split into per-chromosome files using BAMtools v2.5.2 (https://github.com/pezmaster31/bamtools).

For downstream analyses, the Hi-C data was mapped to the hg38 human reference genome assembly with bwa mem v0.7.17-r1188^161^ using the Open2C distiller-nf pipeline (https://github.com/open2c/distiller-nf). Mapped reads were then parsed using pairtools v0.3.0^163^ with the “walks-policy” parameter set to “mask” and the “max_mismatch_bp” parameter set to 1. Read pairs were binned into Hi-C contact matrices and written as MCOOL files. Iterative correction of the matrices and removal of low coverage bins was performed using the “balance” function from cooler v0.8.11^164^.

#### Haplotype phasing

HapCUT2 v1.3.3 was used to phase haplotypes for each individual, based on the mapped linked-reads and Hi-C data^65,162^. Our phasing approach was reference genome-based, as we did not perform de novo genome assembly. First, the VCF files per sample were split by chromosome using BCFtools v1.9 “view” function^156^. Second, the extractHAIRS command was used to convert the input BAM files containing linked-reads or Hi-C data into fragment files with haplotype-relevant information. To include indels alongside the SNPs, the “--indels 1” parameter was enabled at this stage. The fragments originating from these two sequencing technologies were then merged together and used as input for HapCUT2 with the “--hic 1” parameter to enable Hi-C mode, in order to assemble haplotypes.

Since we were interested in the whole-chromosome haplotypes, we retained the largest haploblock with the most heterozygous variants phased per chromosome (MVP block). This haploblock was defined to maximise the product of the proportion of phased heterozygous variants and the covered effective chromosome length. The effective chromosome length was defined as the distance between the last and first variants on the chromosome, in order to account for regions at the beginning and end of chromosomes without any variants to be phased. The phased variants from such identified haploblock were then extracted and combined into phased VCF files using BCFtools v1.9 “concat” function^156^. The statistics of the phased variants were calculated using the “vcfstats” function from RTG Tools (Supplementary Data 2).

### Data subsampling for phasing quality estimation

To estimate the depths of data coverage for haplotype phasing, we performed subsampling of Hi-C and linked-reads data from chromosome 1 for the NA12878 individual. We used “coverage” function from SAMtools v1.15^160^ to calculate the coverage depth of the obtained 10X, TELL-seq, stLFR, and Hi-C data BAM files and downsampled them to match genome-wide coverage depth of 1, 2, 3, 4, 5, 6, 7, 8, 10, 12, 16, 20, 24, and 32X using SAMtools v1.15^160^ with ‘--subsample’ flag. For each combination of subsamplings, we assembled haplotypes for chromosome 1 and extracted phased variants from the MVP block. First, the span of the MVP block and the fraction of phased heterozygous variants in the MVP block were calculated. Second, the phasing results were compared to the high-confidence phased variants of NA12878 from Platinum Genomes^64^ using the calculate_haplotype_statistics.py script from HapCUT2^65^ to calculate phasing error rates. The two error-rates, mismatch and switch, are defined by the fraction of discordant phasing events of either one or multiple variants between the haplotypes generated and that of a Platinum Genomes. To ensure robustness, we performed 20 simulations for each combination of the linked-reads and Hi-C subsamplings and calculated the mean values for each combination to plot (Fig. 1B, C and Supplementary Fig. 1A,B).

### Phasing performance on publicly available data

Publicly available data from unrelated individuals (NA12878, NA20509 and HG03486) and trios (NA19238, NA19239, NA19240, HG00512, HG00513, HG00514, HG00731, HG00732 and HG00733) was used to assess the performance of whole-chromosome haplotype phasing using 10X linked-reads and Hi-C. For each individual, haplotypes were assembled and phased variants extracted from the MVP block for each chromosome. As described above, we calculated the span of the MVP block and the fraction of phased heterozygous variants within it, as well as the phasing error rates, by comparing the resulting haplotypes with the high-quality, haplotype-resolved genomes of these individuals from the Human Genome Structural Variation Consortium^68^.

### Personal genomes FASTA construction

Personal genome FASTA files for downstream analysis were created using phased heterozygous variants obtained from whole-chromosome haplotyping and BCFtools consensus v1.9^156^. This automatically generated chain files for lifting features from the hg38 reference human genome to each personal genome. To create the chain files for opposite lifting from the personal genomes to the reference genome, full-genome alignment was performed using minimap2 v2.24^165^. First, the FASTA files for both haplotype 1 and haplotype 2 were aligned against the hg38 reference genome with the “-cx” flag set to “asm5”. Secondly, the resulting PAF files were converted to chain files using the paf2chain utility (https://github.com/AndreaGuarracino/paf2chain). Lastly, coordinates of the phased variants were lifted to each of the haplotypes for downstream use.

### Personal genomes-based mapping pipelines

#### ATAC-seq data processing

ATAC-seq reads were mapped to the hg38 reference human genome and both haplotypes 1 and 2 of the personal genome using bwa v0.7.17-r1188^161^. Uniquely mapped reads that were not mapped to mitochondrial DNA and had MAPQ > 10 were selected using SAMtools v1.6^160^. Duplicate reads were filtered out using the Picard v2.27.4 “MarkDuplicates” function.

The resulting reads were then assigned to either haplotype 1 or haplotype 2. For each read, the alignment score (AS) tag from the BAM files was compared between haplotypes. Read pairs were assigned to the haplotype based on the sum of AS tags for the R1 and R2 reads. Read pairs with the higher AS values were assigned to either haplotype 1 or haplotype 2, while reads with equal AS values for both haplotypes were left unassigned. To ensure that the assigned reads were truly haplotype-specific and not the result of the mapping biases, we have additionally required them to overlap the phased variants using BEDtools v2.31.1 “pairtobed” function^166^. Similarly, unassigned reads that were required to have no overlap with phased variants.

Peak calling was performed using MACS2 v2.2.6^167^ for the reads mapped to the hg38 reference genome. Peak calling was performed for pooled data and each of the replicates in a narrowPeak mode with the *p*-value cutoff set to 0.01, the mappable genome size “--gsize” set to “hs”, and the “--keep-dup” parameter set to “all”. The consensus peaks were obtained by overlapping the peaks, called for pooled replicates, with peaks from each replicate. The pooled peaks from canonical chromosomes outside the blacklisted regions^168^ that overlapped with at least two replicates by at least 50% were retained. The consensus peaks were then lifted from the hg38 reference genome to the coordinates of haplotypes 1 and 2 of each personal genome using CrossMap v0.6.4^169^ and the chain files generated for each personal genome.

Peak counts were obtained using the BEDtools v2.31.1 “intersect” function^166^. Counts were calculated using the peak files that had been lifted to the personal genomes. Only read pairs corresponding to fragments of less than 2 kb in size were used in the calculation of the counts. The resulting count table was generated by merging the haplotype 1, haplotype 2 and unassigned counts. The total count per peak was calculated as the sum of the haplotype-resolved counts and the average of the unassigned counts, which were obtained independently for each haplotype. To enable comparison between samples, the peaks identified for each individual were merged into one annotation, and another count table was obtained in a manner similar to that described above. The number of informative peaks and coverage statistics were calculated using bioframe v0.3.3^170^.

BigWig coverage tracks were generated separately for total, haplotype 1, and haplotype 2 signals using the deepTools v3.5.1^171^. To generate haplotype-resolved signals, the coordinates of the reads were lifted over to the hg38 reference genome. First, the reads were converted to fragments using BEDtools v2.31.1 “bamtobed” function^166^. Only the fragments of less than 2 kb in size were retained. Second, the reads were lifted to the hg38 coordinates using CrossMap v0.6.4^169^ and the chain files generated for each personal genome. Only the fragments of less than 2.5 kb in size after the liftover procedure were retained. The bigWig coverage tracks for total and haplotype-resolved signals were then generated using the “bamCoverage” function from the deepTools v3.5.1^171^ with the “--effectiveGenomeSize” parameter set to “2913022398”, the “--normalizeUsing” parameter set to “RPGC”, the “--extendReads” parameter enabled, and the “--binSize” parameter set to 50. Genomic tracks plots were produced using pyGenomeTracks v3.8^172^.

#### TT-seq data processing

GENCODE v42 gene annotation^173^ was lifted from the hg38 reference genome to the coordinates of haplotypes 1 and 2 of personal genomes using the liftover utility (https://github.com/jeremymcrae/liftover) and the chain files produced by BCFtools during the personal genomes construction. If the liftover was not successfully performed for a feature, this feature was removed from the annotation. Each haplotype of the personal genomes was then indexed using STAR v2.7.10a^174^ in genomeGenerate mode, using the corresponding personal genome GTF file, to improve the accuracy of the subsequent mapping.

TT-seq reads were mapped to the hg38 reference human genome and both haplotypes 1 and 2 of the personal genome using STAR v2.7.10a^174^ with the “--outFilterMultimapNmax” set to 20, the “--alignSJoverhangMin” set to 999, the “--alignSJDBoverhangMin” set to 1, the “--outFilterMismatchNmax” set to 999, the “--outFilterMismatchNoverReadLmax” set to 0.04. Uniquely mapped reads with MAPQ > 10 were selected using SAMtools v1.6^160^. Duplicate reads were filtered out using the Picard v2.27.4 “MarkDuplicates” function.

The resulting reads were then assigned to either haplotype 1 or haplotype 2. For each read, the alignment score (AS) tag from the BAM files was compared between haplotypes. Read pairs were assigned to the haplotype based on the sum of AS tags for the R1 and R2 reads. Read pairs with the higher AS values were assigned to either haplotype 1 or haplotype 2, while reads with equal AS values for both haplotypes were left unassigned. To ensure that the assigned reads were truly haplotype-specific and not the result of the mapping biases, we have additionally required them to overlap the phased variants using BEDtools v2.31.1 “pairtobed” function^166^. Similarly, unassigned reads that were required to have no overlap with phased variants.

The reads assigned to haplotypes 1 and 2, as well as the unassigned reads, were split by strand using SAMtools v1.6^160^, as previously described^154^. Forward strand reads were extracted by including (i) reads that are second in a read pair (-f 128), excluding reads that are mapped to the reverse strand (-F 16), and (ii) reads that are first in a pair and are mapped to the reverse strand (-f 80). Reverse-strand reads were extracted by including (i) reads that are first in a read pair (-f 64), excluding reads that are mapped to the reverse strand (-F 16) and (ii) reads that are second in a pair and are mapped to the reverse strand (-f 144).

Gene counts were obtained using the”htseq-count” function from HTSeq v0.13.5^175^. Counts were calculated using the gene annotation files lifted to the personal genomes, separately for genes from forward and reverse strands with the parameters “--stranded no”, “--nonunique all”, “--order pos”, and “--type gene”. The resulting counts table was generated by merging the obtained haplotype 1, haplotype 2, and unassigned counts. The total count per gene was calculated as the sum of the haplotype-resolved counts and the average of the unassigned counts, which were obtained independently for each haplotype. To enable comparison between samples, the total gene counts identified for each individual were simply merged together into another count table. The number of informative genes and coverage statistics were calculated using bioframe v0.3.3^170^.

BigWig coverage tracks were generated separately for total, haplotype 1 and haplotype 2 signals using the deepTools v3.5.1^171^. To generate haplotype-resolved signals, the coordinates of the reads were lifted over to the hg38 reference genome. First, the reads were converted to fragments using BEDtools v2.31.1 “bamtobed” function^166^. Second, the reads were lifted to the hg38 coordinates using CrossMap v0.6.4^169^ and the chain files generated for each personal genome. The bigWig coverage tracks for total and haplotype-resolved signals were then generated using the “bamCoverage” function from the deepTools v3.5.1^171^ with the “--effectiveGenomeSize” parameter set to “2913022398”, the “--normalizeUsing” parameter set to “RPGC”, and the “--binSize” parameter set to 50. Genomic tracks plots were produced using pyGenomeTracks v3.8^172^.

#### Publicly available ChIP-seq data processing

Publicly available ChIP-seq datasets (Supplementary Data 1) were processed similarly to the ATAC-seq data. For single-end data, reads were assigned to haplotypes based on the AS tag of each read, and the fragment length requirement was omitted during the liftover procedure. Where available, input-normalised haplotype-resolved signals were generated using the “bamCompare” function from the deepTools v3.5.1^171^ with the “--operation” parameter set to “ratio”. Otherwise, RPGC-normalised haplotype-resolved signals were generated using the “bamCoverage” function from the deepTools v3.5.1^171^ with the “--effectiveGenomeSize” parameter set to “2913022398”, the “--normalizeUsing” parameter set to “RPGC”. The “--binSize” parameter was set to 50 for paired-end data and to 100 for single-end data. The “--smoothLength” parameter was set to 100 for paired-end data and to 200 for single-end data. Genomic tracks plots were produced using pyGenomeTracks v3.8^172^.

### Allele-specific events quantification, linking, and analysis

#### Quantification of allele-specific events

Differentially expressed peaks and genes between individuals were identified using the DESeq2 v1.36.0^176^. Peaks and genes with low counts were filtered by setting a minimum total peak count of 15 and a minimum total gene count of 10 for all samples. Distances between the samples were calculated based on the VST-transformed peak and gene count matrices using the “plotPCA” function. The Wald test implemented in the “nbinomWaldTest” function with default parameters was used to test contrasts between individuals. Peaks with FDR < 0.05 and absolute log2-fold-change > 1 and genes with FDR < 0.1 and absolute log2-fold-change>1 were considered differential (Fig. 2B and Supplementary Fig. 3D).

Allele-specific peaks and genes were identified using the DESeq2 v1.36.0^176^. Peaks and genes with low counts were filtered by requiring half of the haplotype-resolved peak and gene counts within the sample to be greater than 5. Total counts per peak and gene were used to estimate both the library size factors and the dispersions. Dispersions were estimated using local regression by setting the “fitType” parameter to “local”. The Wald test implemented in the “nbinomWaldTest” function with default parameters was used to test contrasts between haplotypes. Peaks with FDR < 0.05 and absolute log2-fold-change>1 and genes with FDR < 0.1 and absolute log2-fold-change> 1 were considered allele-specific (Fig. 2B and Supplementary Fig. 3D and Supplementary Data 3).

#### Validation of allele-specific events

Tornado plots of the histone modifications ChIP-seq signals in the allele-specific ATAC-seq peaks were calculated using the “computeMatrix” function from deepTools v3.5.1^171^ using publicly available data (Supplementary Data 1). Pile-ups were generated in reference-point mode with the “--referencePoint” parameters set to “centre”, the “-a” parameters set to “3000”, the “-b” parameters set to “3000”, and the “--missingDataAsZero” and “--skipZeros” parameters enabled. The resulting matrices were visualised using the “plotHeatmap” function from deepTools v3.5.1^171^.

Population-specific and shared across populations chromatin accessibility QTLs (caQTL) were obtained from Tehranchi et al.^80^. To overlap with the variants from the allele-specific open chromatin peaks, the positions of the caQTL variants were lifted from the hg19 to hg38 reference genome coordinates using CrossMap v0.6.4^169^ and the chain files obtained from the UCSC Genome Browser Database^177^.

Imprinted genes from GeneImprint database which had confirmed imprint status “Imprinted All” were used and converted to Ensembl ID with biomaRt and Ensembl genes v114^178^. Immunoglobulin genes (GO:0019814) and MHC protein complex genes (GO:0042611) were obtained using biomaRt and Ensembl genes v114^178^. Genes exhibiting random allelic and biallelic expression were obtained from Kravitz et al.^85^.

#### Linking allele-specific open chromatin peaks and genes

Putative regulatory links were constructed by linking peaks and annotated gene transcription start sites located within 1 Mb of each other on the linear genome (Supplementary Data 4). This distance was chosen to match the cutoff distance used in population-based studies for association testing. Prior to linking, the peaks and genes likely reflecting non-genetic allele-specific mechanisms were excluded. Peaks originating from the X chromosome were removed from the analyses. Genes originating from the X chromosome, as well as imprinted genes, and immune-related genes (defined by the GO terms mentioned above) were removed from the analyses. The coordinates of the peak centre and the annotated transcription start site of the gene were used to calculate the distance between features. Allele-specific open chromatin peaks were linked to allele-specific genes to create allele-specific putative regulatory links. Similarly, balanced peaks were linked to the balanced genes to create balanced putative regulatory links, which were used as a control set in our analyses. Note that there could be multiple links per peak and gene, both in the case of allele-specific and balanced links.

Following this step, the links were overlapped with TADs annotated for each individual. The TADs were annotated using the insulation score algorithm implemented in the cooltools v.0.3.2 diamond-insulation function^179^ for the 20 kb resolution Hi-C matrices. The window size for insulation score calculations was set to 300 kb. The boundary strength threshold was calculated using the Li method, implemented in the scikit-image package^180^. Bins with a boundary strength above this threshold were considered TAD boundary bins. These bins were converted into TADs by joining two neighbouring bins together continuously. The TAD boundary coordinate was then randomly selected from the coordinates of the joined bins with a significant insulation score. To determine overlap with TADs, the coordinates of the peaks and the gene transcription start site window (2.5 kb upstream and downstream) were compared with the TAD coordinates. If peaks or transcription start sites overlapped multiple TADs, the smallest possible distance was used to determine whether features belonged to the same TAD. To contextualise the links, the chromatin loops from the high-resolution chromosome conformation capture assays Hi-C^63^, promoter capture Hi-C^88^, H3K27ac HiChIP^89^, and Micro-C^90^ from the NA12878 individual were used. Following this analysis, only the links located within the same TAD were retained for downstream analyses.

The links were further annotated as being concordant or discordant, based on the allelic imbalance of the open chromatin peak and gene, determined using the log2-fold-change sign from DESeq2. Concordant links contain an accessible region on the same allele as the active gene and are defined by a positive product of the peak and gene log2-fold-changes. Discordant links contain an accessible region on the opposite allele to the active gene and are defined by the negative product of the peak and gene log2-fold-changes.

#### Integration with population-based data from GTEx

To compare the personal genomics approach with the population-based approach, we used eQTL data from the Genotype-Tissue Expression (GTEx) Consortium, version 8^7^. Specifically, we downloaded significant variant-gene associations, as well as all tested associations, for all available tissues (*N* = 49), along with the lookup table containing all variants genotyped by whole-genome sequencing in the 838 post-mortem donors comprising this dataset. For each peak in the putative regulatory links, the variants underlying the peak were overlapped with the GTEx data (Supplementary Fig. 6A). In addition, we filtered variant-gene pairs for which the linked gene was present in GTEx. This is because GTEx used an earlier version of the gene annotation (v26 in GTEx, v42 in this study), which contained fewer genes.

First, we compared the overlap between the putative regulatory links and the GTEx data. We annotated the variant-gene pairs found in the GTEx significant eQTL set as “eQTL”. The remaining pairs were annotated as “non-eQTL”. Those found in genotyped variants but not tested were annotated as “low minor allele frequency”. Any remaining variant-gene pairs not found in these files were annotated as “not genotyped”. We quantified these four categories for GTEx data from LCLs (Cells – EBV-transformed lymphocytes), while for the comparison with all tissues, we only quantified the “eQTL” category.

Secondly, we compared the agreement between putative regulatory links and the GTEx data. To do this, we focused on variant-gene pairs in the “eQTL” category, comparing the eQTL slope with allelic imbalance in chromatin accessibility, as well as its concordance or discordance with gene expression. The GTEx slope is calculated using a linear regression model between genotype and gene expression, representing the normalised effect size of the eQTL. As the slope is always calculated for the alternative allele versus the reference allele, we phase-corrected it to align with the phasing data. We then compared the phase-corrected slope and log2-fold-change of chromatin accessibility for each variant in the “eQTL” set. The odds ratio for the agreement between GTEx data and putative regulatory links was calculated using Fisher’s exact test. The contingency table was constructed from the following values combined across five individuals: (i) the number of concordant links in agreement, (ii) the number of concordant links in disagreement, (iii) the number of discordant links in agreement, and (iv) the number of discordant links in disagreement.

### Deep learning models of chromatin accessibility

#### ChromBPNet models training and generating predictions

Individual-specific ChromBPNet deep learning models of base-pair-resolution chromatin accessibility were trained using a five-fold chromosome-holdout cross-validation scheme as previously described^23,109,110^. ChromBPNet v0.1.7 pipeline, which was the latest version available at the time of the analysis, was used as described in the repository (https://github.com/kundajelab/chrombpnet). For more extensive details on the implementation and performance of ChromBPNet, we would refer to the original studies by the developers^23,109,110,113^.

The relaxed peak set, which was initially called for pooled data in narrowPeak mode with a *p*-value cutoff of 0.01 (see the *ATAC-seq data processing* section for more details), was used for training. In addition, peaks displaying allelic imbalance (FDR < 0.1, a more lenient threshold than that used for allele-specific analyses) were excluded from the training procedure to enable evaluation of these regions later. The negative set of GC-matching non-peaks for training was obtained using the “prep nonpeaks” command from chrombpnet v0.1.7^23^.

The training, test, and validation chromosomes used for each of the five folds are as follows. Test chromosomes: fold 0 (chr1, chr3, chr6), fold 1 (chr2, chr8, chr9, chr16), fold 2 (chr4, chr11, chr12, chr15), fold 3 (chr5, chr10, chr14, chr18, chr20, chr22), fold 4 (chr7, chr13, chr17, chr19, chr21, chrX). Validation chromosomes: fold 0 (chr8, chr20), fold 1 (chr12, chr17), fold 2 (chr7, chr22), fold 3 (chr6, chr21), fold 4 (chr10, chr18). The bias models for each individual were trained using the “bias pipeline” command from chrombpnet v0.1.7^23^ in the “ATAC” mode using the hg38 reference genome, BAM files for merged replicates, peak files described above, and the”--bias-threshold-factor” set to 0.5, as recommended by the authors. The bias-factorised ChromBPNet models for each individual were then trained using the “pipeline” command from chrombpnet v0.1.7^23^ using the same files and the trained in the previous bias models. Performance was evaluated using two built-in metrics: Pearson correlation between total observed and predicted counts, and Jensen-Shannon distance between observed and predicted profile shapes.

Since models trained for each fold demonstrated similar evaluation metrics (Supplementary Fig. 7A), we used the fold 0 ChromBPNet models of each individual to predict the total counts and profile contribution scores. The consensus peaks generated for (i) the hg38 reference genome coordinates and (ii) lifted to the coordinates of haplotypes 1 and 2 of each personal genome were then used to generate predictions with the trained ChromBPNet models. These were generated using the ‘pred_bw’ and ‘contribs_bw’ commands from chrombpnet v0.1.7^23^ with (i) the FASTA files and peak coordinates of the reference genome and (ii) the FASTA files and peak coordinates of haplotypes 1 and 2 of the personal genomes. The bigWig files generated for each haplotype of the personal genome were then lifted to the hg38 reference genome coordinates for visualisation and downstream analysis using CrossMap v0.6.4^169^ and the chain files generated for each personal genome.

#### Annotation of transcription factor motifs in LCLs

TF-MoDISco lite v2.2.0^181^ was used to discover motifs de novo from the ChromBPNet profile contribution scores predicted for the hg38 reference genome. TF-MoDISco identifies and extracts high-contribution score sequences (seqlets) from all scores predicted by a ChromBPNet model. TF-MoDISco was run using the “modisco motifs” command and a maximum of 1000000 seqlets. The motif position frequency matrices (PFMs) discovered by TF-MoDISco were extracted using the “modisco meme” command. The PFMs were matched to the known motifs using the JASPAR 2024 CORE vertebrates collection of non-redundant transcription factor motifs^112^. The top five motif matches for each PFM were retained using the “modisco report” command, which used Tomtom v4.11.2^182^. The top-scoring motifs were then matched to JASPAR 2024 CORE vertebrate motif clusters (https://jaspar.elixir.no/matrix-clusters/) using their JASPAR motif IDs. A weighted majority voting system was then used to assign each PFM to a JASPAR cluster. This was done to simplify the subsequent annotation of the de novo motifs, since the Tomtom matching process is sensitive to subtle differences in PFM nucleotide frequencies and does not always result in the same JASPAR motif instances being the top-scoring ones for motifs detected in different individuals. Nevertheless, most of the top-scoring motifs would likely belong to the same JASPAR motif cluster.

To reduce complexity and retain the motifs consistently discovered across multiple individuals, we performed motif clustering and annotation, similarly to the original approach described previously^111^ (https://github.com/vierstralab/motif-clustering). Since TF-MoDISco PFMs were all of 30 bp length, we trimmed them to keep only the relevant positions. Using the relative information content of each position, for each PFM, we removed positions constituting 5% of the relative information content from the left and right sides of the motif. Next, we calculated pairwise similarities between the trimmed motifs annotated for all individuals using Tomtom v4.11.2^182^ with the “-dist” parameter set to “kullback”, the “-motif-pseudo” parameter set to “0.1”, and the “-min-overlap” parameter set to “1”. The resulting Tomtom similarity E-value pairwise similarity matrix was used as input for the clustering pipeline. Before clustering, we manually removed TF motif instances that clearly represented repetitive regions, as these could confound the clustering results. Clustering was performed using complete-linkage hierarchical clustering with the correlation metric on the TomTom similarity E-values. The clusters were identified by cutting the dendrogram at a height of 0.6. This approach produced *N* = 61 clusters, one of which we further refined by splitting it in two, cutting the dendrogram at a height of 0.4.

The consensus motif PFMs for each cluster were then obtained by averaging the individual motifs within the cluster, with the weight of each motif calculated based on the number of seqlets from which it was derived. These consensus motif PFMs were then trimmed using relative information content, in a manner similar to that employed for the initial TF-MoDISco PFMs. These consensus motifs for all clusters were then manually filtered based on their content and annotated using the assigned above JASPAR clusters. This resulted in a final set of *N* = 34 non-redundant consensus motif PFMs representing the syntax of *N* = 24 transcription factor groups that drive gene expression in LCLs (Fig. 3B).

#### Comparison of ATAC-seq data with deep learning predictions

To evaluate the performance of the deep learning models, the experimentally measured chromatin accessibility was compared with the ChromBPNet contribution scores predicted for each haplotype. To this end, contribution scores in ± 25 bp windows around each variant were used to calculate the difference in contribution scores between haplotypes. For SNPs, this meant using a 51 bp window centred on the variant position; for indels, the window was larger by an offset equal to the indel size. The obtained contribution score differences were then compared to the measured chromatin accessibility log2-fold-changes in the peaks in which the variant was located (see *Quantification of allele-specific events* section for more details). This implies that all variants underlying the peaks would have the same experimentally measured chromatin accessibility difference assigned to them. The Pearson correlation coefficient was then computed between the measured and predicted values.

#### Annotation of disrupted TF motifs

To annotate disrupted transcription factor motifs, we used the contribution scores predicted by ChromBPNet for each haplotype and de novo motif syntax of LCL transcription factors (see *Annotation of transcription factor motifs in LCLs* section for more details). For each variant and each consensus motif PFM, we scanned the nucleotides surrounding the variant for motif matches by shifting the PFM in 1 bp steps. For both haplotype 1 and haplotype 2, we computed the Pearson correlation coefficient between the values from the consensus motif PFM corresponding to the sequence nucleotide bases and the predicted contribution scores at each position. Consensus motif PFMs were evaluated in both regular and reverse complement directions. This analysis used the variants underlying allele-specific and balanced open chromatin peaks.

In the case of SNPs, the window size surrounding the variant nucleotides was always the same for both haplotype 1 and haplotype 2. Therefore, a one-step motif-matching process was used to detect motif hits on each haplotype. In the case of indels, however, the window size varies between haplotype 1 and haplotype 2. Therefore, we used a two-step motif-matching process to detect motif hits on each haplotype that could be created by inserting or deleting multiple nucleotides. To achieve this, we first determined which haplotype had the longer nucleotide sequence, then filled the gap in the other haplotype with N bases to match their sizes. Two rounds of motif matching were then performed. The first round was performed on the haplotypes before size matching, in order to determine the motif matches on the “short” haplotype that were not present on the “long” haplotype. The second round was performed on the size-matched, N-filled haplotypes to determine motif matches on the “long” haplotype that were not present on the “short” haplotype. Following the matching process, we only retained motif hit positions with a Pearson correlation greater than 0.7 on at least one haplotype. In addition, we only kept motif hits that had the same sign for the difference in contribution scores and Pearson correlations between haplotypes. Lastly, for each variant, the top-scoring motif hit was determined based on the highest Pearson correlation coefficient.

The following filters were applied to annotate variants that disrupt motifs between haplotypes. First, the difference in contribution scores within the ± 25 bp windows surrounding each variant, as calculated above, had to be the same sign as the measured log2-fold-change in chromatin accessibility in the peak. Second, the difference in contribution scores within the positions of motif hits overlapping the variant had to be the same as the measured log2-fold-change in chromatin accessibility in the peak. This ensured that the difference in the ± 25 bp windows around each variant was driven by the difference in contribution scores within the motif overlapping the variant. Third, the difference in predicted contribution scores for ± 25 bp windows surrounding each variant had to be greater than 0.02 between haplotypes to ensure that the variant belonged to open chromatin peaks that could be reliably explained by the ChromBPNet model. This cutoff was chosen to keep the fraction of explained variants underlying balanced peaks below 10%, while keeping the fraction of explained variants underlying allele-specific peaks at ~ 35%. Variants that passed these filters were considered to disrupt TF motifs, and the motif hits associated with them were considered to be disrupted regulatory motifs. The data on the annotated variants disrupting TF motifs was then integrated with identified putative regulatory links, as well as with GTEx eQTL data from LCLs.

#### Validation of disrupted TF binding

To create a control set of NA12878 peaks containing intact regulatory motifs, the same scanning procedure was used with minor modifications. First, scanning was performed using the hg38 reference genome coordinates, the liftover contribution scores for haplotypes 1 and 2, and the hg38 coordinates of the allele-specific peaks. This ensured that the underlying nucleotide sequence size for both haplotypes 1 and 2 was the same. Second, in order for a motif to be considered a hit, the Person correlation had to be higher than 0.7 for both haplotypes, not just one. Thirdly, the sum of the contribution scores within motif hits overlapping the variant had to be greater than 0.01 for both haplotypes to ensure these were motifs with high contribution scores. The motifs found this way were considered to be intact between the haplotypes, and the allele-specific peaks which contained these motifs were then used as a control set. Motifs found in this way were considered intact between the haplotypes, and the allele-specific peaks containing these motifs were used as a control set.

Tornado plots of the transcription factors ChIP-seq signals in the allele-specific and balanced ATAC-seq peaks with motif disruptions, as well as in control ATAC-seq peaks with intact motifs, were calculated using the “computeMatrix” function from deepTools v3.5.1^171^. Pile-ups were generated in reference-point mode with the “--referencePoint” parameters set to “centre”, the “-a” parameters set to “2000”, the “-b” parameters set to “2000”, and the “--missingDataAsZero” and “--skipZeros” parameters enabled. The resulting matrices were visualised using the “plotHeatmap” function from deepTools v3.5.1^171^.

### Validation of the rs545467951 non-coding variant

#### Cell lysate preparation

Crude nuclear extracts (NE) of LCLs were prepared as described before^183^. Briefly, cells were harvested, centrifuged at 400 g for 5 minutes at 4 °C. The obtained cell pellet was washed twice with ice-cold PBS. The cell pellet was resuspended in 5 volumes of buffer A (10 mM HEPES KOH pH 7.9, 15 mM MgCl2, 10 mM KCl) and incubated for 10 min on ice. Cell pellets were obtained by centrifugation (400 g, 5 min, 4 °C) and then resuspended in 2 volumes of freshly made buffer A+ (buffer A supplemented with 0.15% NP-40 and EDTA-free complete protease inhibitor). Then, cells were lysed by dounce homogenisation (40 strokes, 30 s rest on ice every 10 strokes), and crude nuclei were collected by centrifugation (3200 g, 15 min, 4 °C). Crude nuclei were washed with ice-cold PBS (10 times volume of pellet), and clean crude nuclei were collected by centrifugation (3200 × *g*, 5 min, 4 °C). Crude nuclei were resuspended in buffer C (420 mM NaCl, 20 mM HEPES, pH 7.9, 20% (v/v) glycerol, 2 mM MgCl2, 0.2 mM EDTA, supplemented with 0.1% NP-40, CPI, and 0.5 mM DTT) and incubated for 90 min while rotating at 4 °C. Afterward, the nuclear lysate was centrifuged (20,000 × *g*, 30 min, 4 °C) and the soluble nuclear fraction was collected. Obtained NE were aliquoted, snap-frozen in liquid nitrogen, and stored at − 80 °C.

#### DNA pull-downs

DNA oligonucleotides were ordered from Integrated DNA Technologies (IDT) with 5′-biotinylation of the forward strand. Oligonucleotides were annealed as described previously^184^. DNA pull-downs were performed in duplicate as described previously^133^. Briefly, for each reaction, 20 μL of streptavidin sepharose bead slurry (GE Healthcare. Cat. #GE17-5113-01) was equilibrated by washing once with 1 mL of PBS containing 1% NP-40 and twice with 1 mL of DNA binding buffer (DBB; 1 M NaCl, 10 mM Tris, pH 8.0, 1 mM EDTA, 0.05% NP-40). Next, 500 pmol of previously annealed DNA oligonucleotides were incubated with the beads in 600 μL DBB final volume (30 min of rotating at 4 °C), beads were then washed twice with DBB and once with protein incubation buffer (PIB; 150 mM NaCl, 50 mM Tris pH 8.0, CPI, 0.25% NP-40, 1 mM DTT). Per pull-down, 500 μg of NE was incubated with the beads in a total volume of 600 μL PIB (90 min rotating at 4 °C). After the incubation beads were washed three times with 1 mL of PIB and twice with 1 mL of PBS. Excess PBS was removed using a syringe, and beads were immediately resuspended in 50 μL of elution buffer (2 M urea, 100 mM Tris, pH 8.5, 10 mM DTT) and incubated for 20 min while shaking at room temperature. Samples were then incubated with iodoacetamide (50 mM final concentration) for 10 mins in the dark while shaking. Next, proteins were digested using 0.25 μg of trypsin and incubated while shaking for 2 h at room temperature. Afterward, samples were spun down, and the supernatant was collected. Beads were washed once more with 50 μL of elution buffer, and the supernatant was collected and added to the previously collected supernatant, 0.1 μg of trypsin was added to the mix and incubated overnight. The next day, samples were purified using StageTips. Dimethyl labelling on StageTips was done as described previously^185^.

#### Mass spectrometry

Samples were analysed by LC-MS/MS on an Orbitrap Exploris 480 mass spectrometer connected to an Evosep One LC system (Evosep). Prior to LC separation with the Evosep One, peptides were resuspended in 0.1% formic acid and 20% of the sample was loaded on Evotip Pure tips (Evosep, Cat. #EV2018). Elution was carried out directly on-column, employing the “Extended Method” (88-minute gradient) with the performance column (Evosep, Cat. #EV1137) combined with the emitter (Evosep, Cat. #EV1086). Ionisation was achieved using the Easy-Spray NG Ion Source (Thermo Scientific), with a spray voltage maintained between 1.7 and 2 kV. The Orbitrap Exploris 480 was configured in data-dependent acquisition (DDA) cycle time mode with a 1-second duty cycle, acquiring full MS scans in the Orbitrap analyser at a resolution of 60,000 (at m/z 200) and scanning across the 375–1500 m/z range. The default charge state was set at 2 + , with the AGC target selected as “standard” for both MS1 and ddMS2 scans, and the injection time mode set to “auto” for both. Monoisotopic peak determination was optimised for peptides, while dynamic exclusion was limited to 20 seconds. For ddMS2 acquisition, precursor ions within the 2 + to 6 + charge range that exceeded a minimum intensity threshold of 5e4 were selected, and fragmentation was induced at a normalised higher-energy collisional dissociation (HCD) energy of 30%. Precursors were isolated in the quadrupole analyser with a 1.2 m/z window, and MS2 spectra were acquired at 15,000 resolution in the Orbitrap analyser.

#### Mass spectrometry data analysis

All raw mass spectrometry spectra were processed using Maxquant software v2.4.9.0^186^ (Max Planck Institute of Biochemistry). Protein identification was accomplished by searching against the UniProt human proteome reference database^187^ (2024 release), and common contaminants were systematically filtered out from the results. Dimethylation-labelled samples were analysed with a workflow adapted from the standard 3plex dimethylation method. Peptide quantification relied on identifying both light-dimethyl-modified peptides (+28.031 Da) and heavy-dimethyl-modified peptides (+ 32.056 Da), with low-abundance resampling enabled to improve sensitivity. Only proteins that were quantified in all 4 channels were used for downstream analysis. Outlier statistics were used to identify significant proteins. Proteins were considered significant with one interquartile range for both forward and reverse experiments.

#### CRISPR-Cas9-mediated variant editing

Editing of the rs545467951 non-coding variant within the distal enhancer of the *PIK3R5* gene was performed according to the protocol described by Johnston et al.^126^. gRNA were designed using the CRISPOR website^188^ and cloned into the pSpCas9(BB)-2A-GFP (PX458, Addgene #48138) plasmid. The 200-nt repair template containing the desired G > T mutation was designed and ordered from IDT as an Alt-R ssODN template. Following the G > T edit, the PAM sequence within the OCT motif on haplotype 1 is destroyed, thereby preventing further re-cutting by Cas9.

The HG03464 cells were passaged 48 and 24 h before transfection at a concentration of 4 × 10^5^ cells/ml in RPMI medium without penicillin/streptomycin. A total of 4 × 10^6^ cells were transfected with 33.3 μg of Cas9-GFP U6-gRNA plasmid and 0.4 nmol of ssODN template on the Amaxa Nucleofector II (Lonza) using the U009 programme. Transfections were conducted using the Mouse Embryonic Stem Cell Nucleofector Kit (Lonza, Cat. #VPH-1001). After transfection, the cells were immediately resuspended in RPMI medium without penicillin/streptomycin and transferred to T25 flasks at a final volume of 4 ml (3 ml of RPMI medium without penicillin/streptomycin and 1 ml of conditioned and filtered RPMI medium without penicillin/streptomycin). 48 h after transfection, the GFP-positive cells were sorted in bulk using a FACSAria Fusion Flow Cytometer (BD Biosciences) with FACSDiva software v8.0.1, after which the pools were expanded.

To genotype the edited cells, the gDNA from the wild-type and the edited pool was extracted with ISOLATE II Genomic DNA Kit (Meridian Bioscience, Cat. #BIO-52067) and then amplified using Q5 High-Fidelity DNA Polymerase (NEB, Cat. #M0491L). PCR products were extracted with ISOLATE II PCR and Gel Kit (Meridian Bioscience, Cat. #BIO-52060), and G > T editing was validated by Sanger sequencing. To quantify template-directed CRISPR/Cas9 editing, PCR primers containing designed mutations were used to produce the homozygous G and T alleles from gDNA of the wild-type. To assess the HDR-mediated editing rate and indel spectrum, TIDER v5.0.5^134^ was used to decompose the wild-type and edited pool using Sanger traces from the homozygous alleles. The fraction of the T allele was considered to be the fraction of HDR, from which the fraction of the G allele was calculated, so that their sum is equal to 1, not considering the indel fraction. The HDR editing rate was then calculated from the G and T allele fractions of the wild-type and edited pool. Sanger sequencing tracks were visualised using the sangerseqR v1.32.0 package^189^.

#### Sanger sequencing and RT-qPCR

The HG03464 cells were harvested and lysed in the RLT buffer. RNA was isolated from the wild-type and edited pool using a RNeasy Kit (Qiagen, Cat. #74104), then treated with DNase I (Qiagen, Cat. #79254). The purified RNA was then reverse transcribed into cDNA using the iScript cDNA Synthesis Kit (Bio-Rad, Cat. #1708891).

To quantify *PIK3R5* cDNA, the reverse-transcribed cDNA was first treated with RNase H (Thermo Scientific, Cat. #EN201) and then amplified using Q5 High-Fidelity DNA Polymerase (NEB, Cat. #M0491L). The PCR products were then extracted using an ISOLATE II PCR and Gel Kit (Meridian Bioscience, Cat. #BIO-52060), after which the transcripts produced from each haplotype in the wild-type and edited pool were measured by Sanger sequencing. Sanger sequencing tracks were visualised using the sangerseqR v1.32.0 package^189^.

*PIK3R5* gene expression was quantified using RT-qPCR with a SensiFAST SYBR No-ROX Kit (Meridian Bioscience, Cat. #BIO-98005), with previously published primers for *PIK3R5* and the housekeeping genes *ACTB* and *GAPDH*^126,190^. RT-qPCR was performed in quadruplicates. The Ct values for the *PIK3R5* gene were normalised to the mean of the *ACTB* and *GAPDH* values to obtain ∆Ct values. Expression ratios (2^−∆∆Ct^) were computed relative to the geometric mean of the wild-type conditions. Differential testing between conditions was performed on ∆Ct values using a two-sided *T* test.

### Reporting summary

Further information on research design is available in the Nature Portfolio Reporting Summary linked to this article.

## Supplementary information

Supplementary Information

Description of Additional Supplementary Files

Supplementary Data 1

Supplementary Data 2

Supplementary Data 3

Supplementary Data 4

Supplementary Data 5

Reporting Summary

Transparent Peer Review file

## Data Availability

Generated linked-reads, Hi-C, ATAC-seq, and TT-seq data have been deposited at the GEO database (GSE308298) and are publicly available as of the date of publication. Proteomics data have been deposited at the PRIDE database (PXD069116) and are publicly available as of the date of publication. Previously published datasets used in this study are listed in Supplementary Data 1. Oligonucleotides, primers, and gRNA sequence are listed in Supplementary Table 1.

## Code availability

Supporting data and original code necessary to reproduce the results presented in this manuscript have been deposited at Zenodo (10.5281/zenodo.17285188).
