# Supplementary material for: Mapping functional non-coding variation in individual human genomes through haplotyping, multiomics, and deep learning
Source: Nat Commun. 2026 Apr 29;17:5856. doi: 10.1038/s41467-026-72392-x (PMC13333882; doi:10.1038/s41467-026-72392-x)
Supplement: Supplementary file 1 — Supplementary Information [file 41467_2026_72392_MOESM1_ESM.pdf]

Supplementary Information

Mapping functional non-coding variation in individual human genomes through haplotyping, multiomics, and deep learning

Mikhail D. Magnitov, Robin H. van der Weide[†], Aster F. Witvliet[†], Miguel Hernández Quiles, Moreno Martinović, Hans Teunissen, Luca Braccioli, Michiel Vermeulen, Elzo de Wit

oligo_pull_down_OCT_motif_G_allele_fw	/5Biosg/CCCTGCCAAGAC**ATTGGCAT**TTTAAATTCTTTC
oligo_pull_down_OCT_motif_G_allele_rv	GAAAGAATTTAAA**ATGCCAAT**GTCTTGGCAGGG
oligo_pull_down_OCT_motif_T_allele_fw	/5Biosg/CCCTGCCAAGAC**ATTTGCAT**TTTAAATTCTTTC
oligo_pull_down_OCT_motif_T_allele_rv	GAAAGAATTTAAA**ATGCAAAT**GTCTTGGCAGGG
editing_ssODN	GGTAATCCCCTCCCCCAACCCCACAACTCTCTTATCCTTTTCAAAATAAAAAAGAATCATTTCGGTCTTTGGTGGAAACCCAACGCCCTGCCAAGAC**ATTTGCAT**TTTAAATTCTTTCTCTGAGCGTACTCGAAAGGAAGAAAGGCATTGCCTCCTAGCAAAGGAACTTCAGTCACCAACCAGAAGCTCAGAAGGCATTC
editing_gRNA_fw	CCAACGCCCTGCCAAGACAT
primer_genotyping_gDNA_fw	ATTCTACTTCCGGCTCCAGC
primer_genotyping_gDNA_rv	ACATGCACCAGGTCCTGTTT
primer_cDNA_PIK3R5_fw	TGATGAACTTCTGCCCAGGC
primer_cDNA_PIK3R5_rv	TGGCAGAGCTTGAGGACATC
primter_TIDER_G-allele_fw	CCAAGACATTGGCATTTTAAATTC
primer_TIDER_G-allele_rv	GAATTTAAAATGCCAATGTCTTGG
primer_TIDER_T-allele_fw	CCAAGACATTTGCATTTTAAATTC
primer_TIDER_T-allele_rv	GAATTTAAAATGCAAATGTCTTGG
primer_qPCR_PIK3R5_fw	TGACATGCTACTCTACTACTG
primer_qPCR_PIK3R5_rv	GGAGTGGATGAAGATCTCTG
primer_qPCR_ACTB_fw	CCCTGGCACCCAGCAC
primer_qPCR_ACTB_rv	GCCGATCCACACGGAGTAC
primer_qPCR_GAPDH_fw	TCGGAGTCAACGGATTTG
primer_qPCR_GAPDH_rv	CAACAATATCCACTTTACCAGAG

Supplementary Table 1. Oligonucleotides, primers, and gRNA sequence used in this study.

Supplementary Figure 1

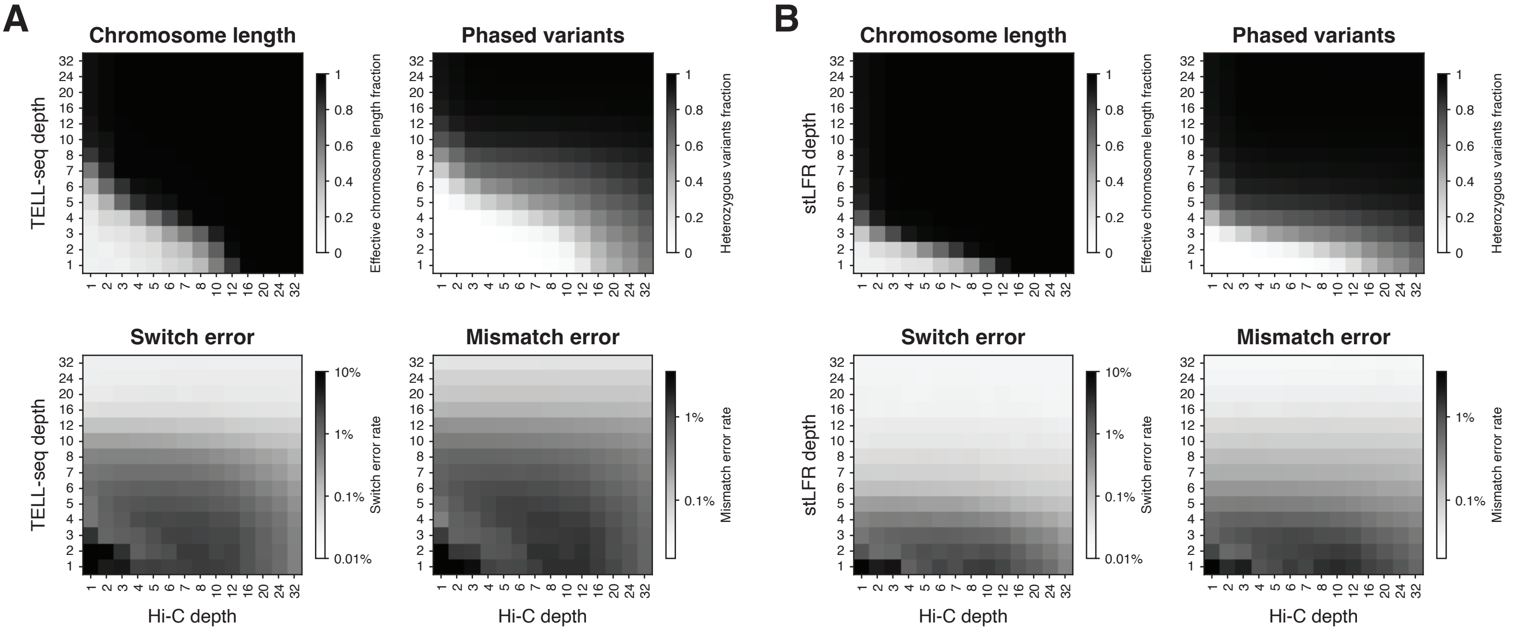

Supplementary Figure 1. Haplotype phasing using subsampling of TELL-seq and stLFR linked-reads in combination with Hi-C data. (A) Evaluation of the fraction of chromosome length and the fraction of phased heterozygous variants (top row), as well as the switch and mismatch error rates (bottom row), obtained during the haplotype phasing procedure using TELL-seq linked-reads and Hi-C data subsampled to different genome coverages for the NA12878 individual[1,2]. **(B)** Evaluation of the fraction of chromosome length and the fraction of phased heterozygous variants (top row), as well as the switch and mismatch error rates (bottom row), obtained during the haplotype phasing procedure using stLFR linked-reads and Hi-C data subsampled to different genome coverages for the NA12878 individual[2,3].

Supplementary Figure 2

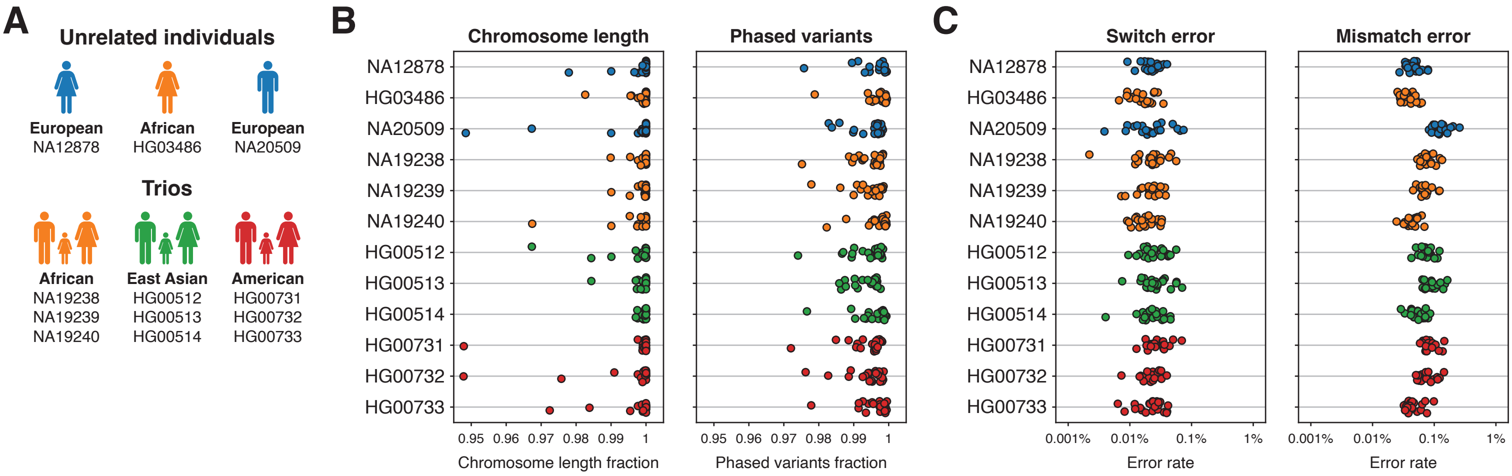

Supplementary Figure 2. Haplotype phasing of individual human genomes using publicly available 10X linked-reads and Hi-C data. (A) The overview of the three unrelated individuals and three trios for which whole-chromosome haplotypes were reconstructed using publicly available 10X linked-reads and Hi-C data. The obtained haplotypes were then compared to published haplotype-resolved genomes of these individuals to test the utility of our approach. The samples are coloured according to their super-population of origin. **(B)** The fraction of chromosome length and the fraction of phased heterozygous variants obtained during the haplotype phasing procedure using 10X linked-reads and Hi-C data for the individuals from (A). Each data point represents values for a single chromosome. **(C)** Switch and mismatch error rates obtained during the haplotype phasing procedure using 10X linked-reads and Hi-C data for the individuals from (A). The obtained haplotypes were compared to the high-quality haplotype-resolved genomes of these individuals from The Human Genome Structural Variation Consortium[4]. Each data point represents values for a single chromosome.

Supplementary Figure 3

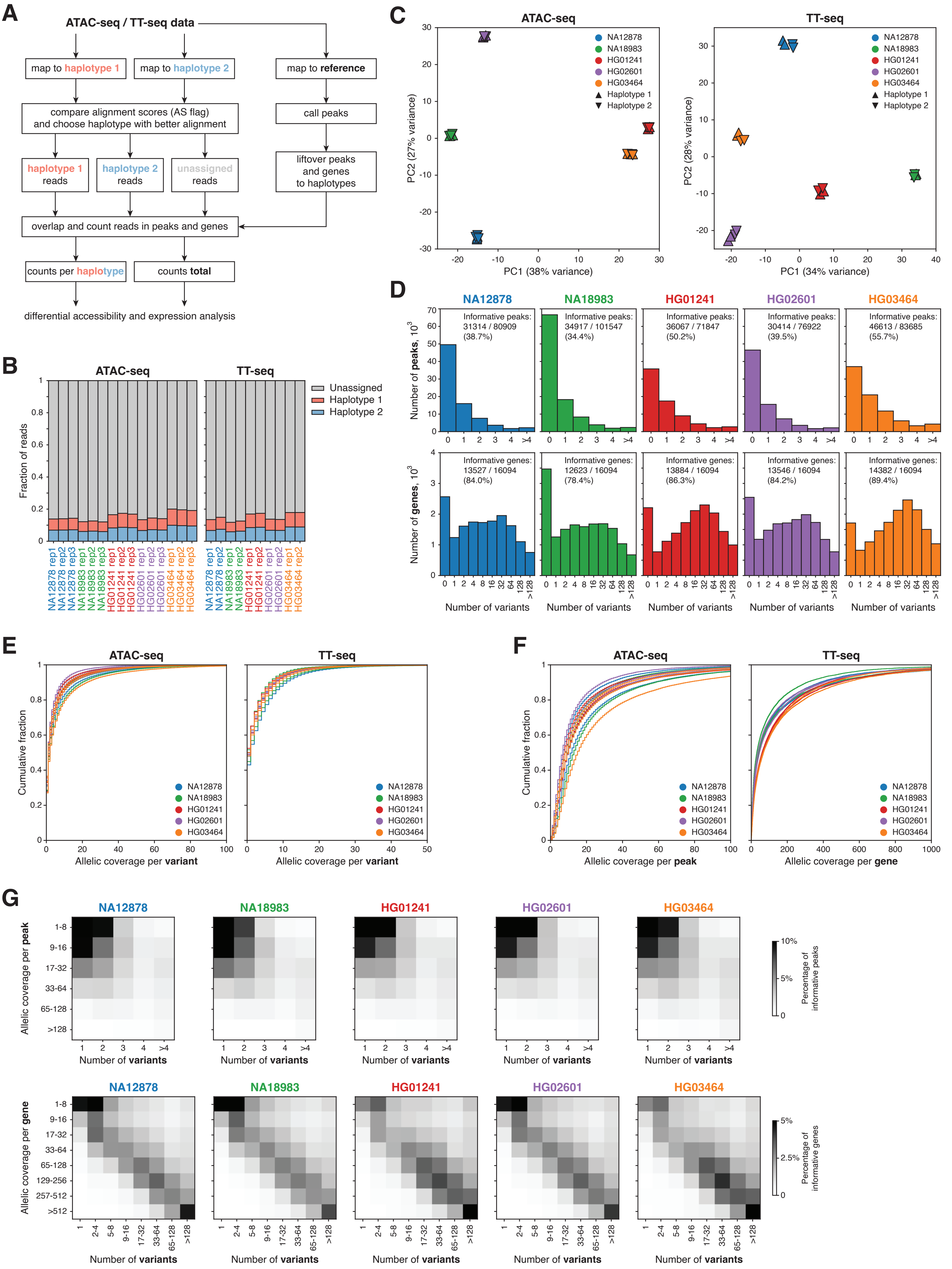

Supplementary Figure 3. Personal genome-based quantification of haplotype-specific signals in ATAC-seq and TT-seq data. (A) Schematic representation of the personal genome-based allele-specific pipeline for the quantification of differential ATAC-seq and TT-seq data. **(B)** Quantification of ATAC-seq reads (top panel) and TT-seq reads (bottom panel) assigned to haplotype 1 (red), haplotype 2 (blue) or unassigned (grey). **(C)** Principal component analysis of the ATAC-seq and TT-seq data. **(D)** Histograms showing the number of phased heterozygous variants that overlap with ATAC-seq peaks (top row) and expressed TT-seq genes (bottom row), for each individual. Informative peaks and genes are defined as those with at least one heterozygous variant. **(E)** Cumulative distribution of allelic read coverage for each phased heterozygous variant within informative ATAC-seq peaks (left panel) and TT-seq genes (right panel). Data for each replicate for each individual is shown as a separate distribution. **(F)** Cumulative distribution of allelic read coverage per informative peak (left panel) and gene (right panel). Coverage was obtained by aggregating the coverage across all variants within peaks and genes for ATAC-seq and TT-seq, respectively. Data for each replicate for each individual are shown as separate distributions. **(G)** Heatmap showing the fraction of informative ATAC-seq peaks (top row) and expressed TT-seq genes (bottom row) as a function of the number of heterozygous variants and the allelic coverage for each individual.

Supplementary Figure 4

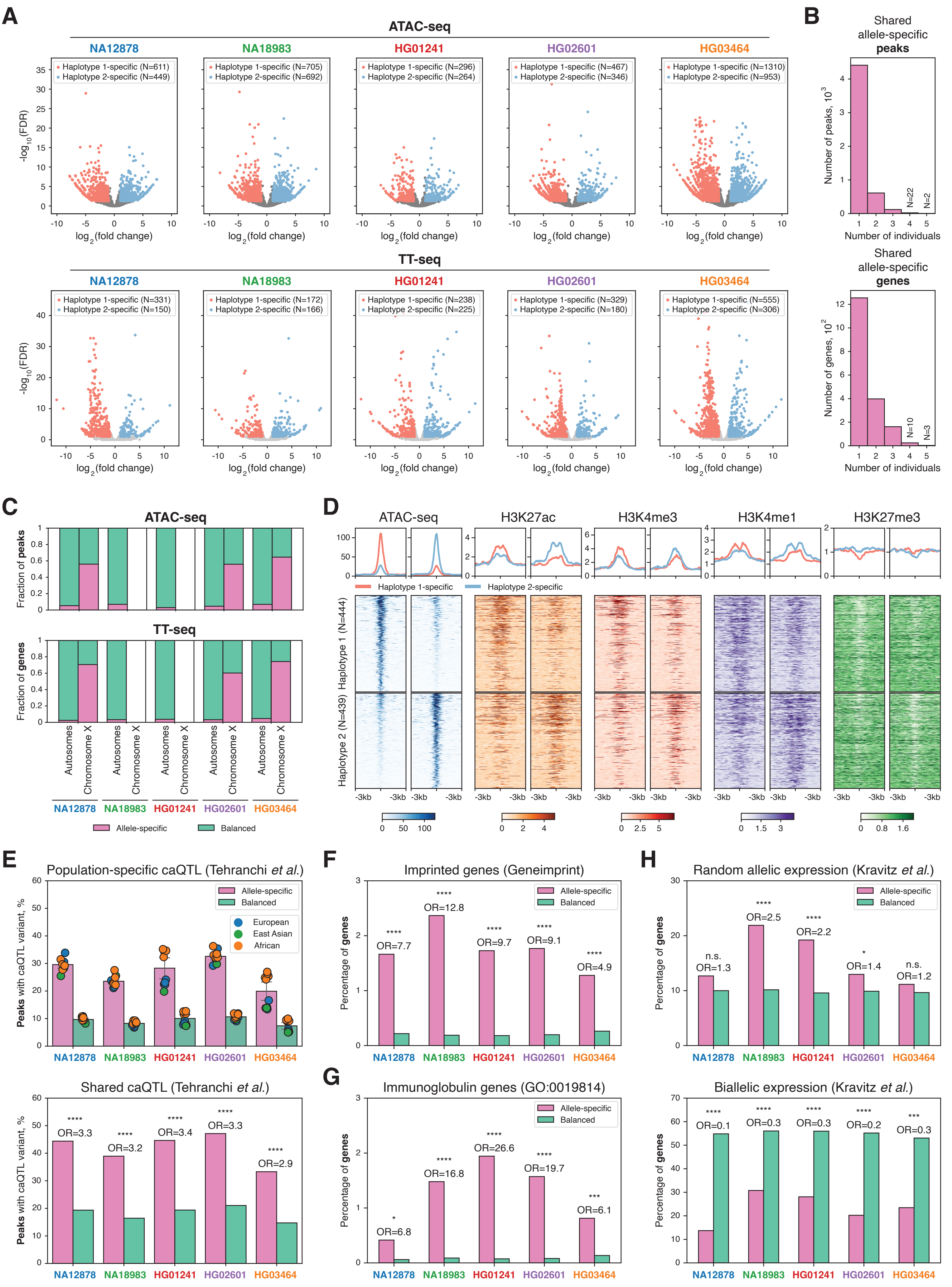

Supplementary Figure 4. Quantification and validation of the allele-specific open chromatin peaks and genes. (A) Volcano plots showing the effect sizes and statistical significance of allele-specific peaks (top row) and genes (bottom row), as measured by ATAC-seq and TT-seq, for each individual. The number of allele-specific events for haplotype 1 (red) and haplotype 2 (blue) are indicated at the top of each panel. **(B)** Bar plots showing the number of allele-specific peaks (top panel) and genes (bottom panel) that overlap between individuals. **(C)** Quantification of allele-specific and balanced open chromatin peaks (top panel) and genes (bottom panel) from the autosomes and X chromosomes of each sample. The X chromosome values for NA18983 and HG01241 are not available, as these samples are from male individuals. **(D)** Tornado plots showing ATAC-seq and the ChIP-seq signals for the histone modifications H3K27ac, H3K4me3, H3K4me1 and H3K27me3 at the allele-specific peaks of the NA12878 individual. The signals from haplotype 1 and haplotype 2 are shown in red and blue, respectively. Publicly available ChIP-seq data from the NA12878 individual were used[5]. **(E)** The percentages of allele-specific (in pink) and balanced (in turquoise) peaks containing population-specific (top panel) or shared across populations (bottom panel) chromatin accessibility QTLs[6] for each individual. Each data point represents a distinct chromatin accessibility QTL set from European, East Asian, and African populations. Odds ratios are from Fisher's exact test (****p<0.0001, ***p<0.001, **p<0.01, *p<0.05, n.s. stands for not significant). **(F)** The percentages of allele-specific (in pink) and balanced (in turquoise) genes that overlap with the imprinted genes from the GeneImprint database[7] for each individual. Odds ratios are from Fisher's exact test (****p<0.0001, ***p<0.001, **p<0.01, *p<0.05, n.s. stands for not significant). **(G)** The percentages of allele-specific (pink) and balanced (turquoise) genes that overlap with the immunoglobulin gene set (GO:0019814) from the Gene Ontology knowledgebase[8] for each individual. Odds ratios are from Fisher's exact test (****p<0.0001, ***p<0.001, **p<0.01, *p<0.05, n.s. stands for not significant). **(H)** The percentages of allele-specific (in pink) and balanced (in turquoise) genes that overlap with genes exhibiting random allelic (top panel) and biallelic (bottom panel) expression[9] for each individual. Odds ratios are from Fisher's exact test (****p<0.0001, ***p<0.001, **p<0.01, *p<0.05, n.s. stands for not significant).

Supplementary Figure 5

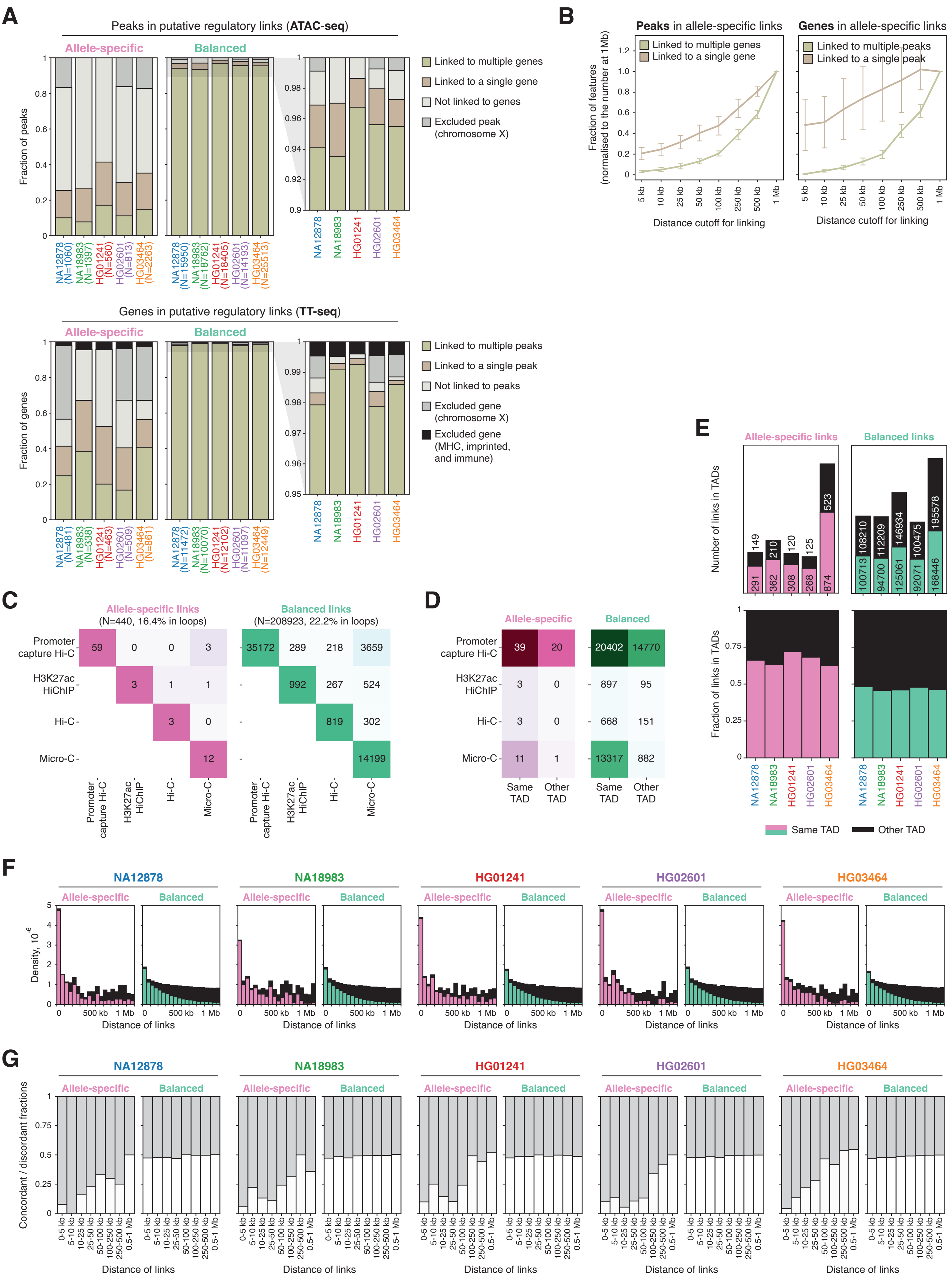

Supplementary Figure 5. Construction of putative regulatory links between allele-specific open chromatin peaks and genes. (A) Quantification of peaks (top panel) and genes (bottom panel) comprising allele-specific and balanced putative regulatory links. The peaks can be (i) linked to multiple genes, (ii) linked to a single gene, (iii) not linked to any gene, or (iv) excluded due to being on the X chromosome. The genes can be (i) linked to multiple peaks, (ii) linked to a single peak, (iii) not linked to any peak, (iv) excluded due to being on the X chromosome, or (iv) excluded due to being imprinted or immune-related genes. **(B)** Quantification of peaks and genes linked to single and multiple genes and peaks, respectively, as a function of distance. The fractions at various distances are normalised to the total number at a distance of one megabase. **(C)** The number of allele-specific and balanced putative regulatory links that overlap with the interactions derived from Hi-C[2], promoter capture Hi-C[10], H3K27ac HiChIP[11], and Micro-C[12] in the NA12878 individual. On-diagonal values show the total number of overlaps between regulatory links and interactions/loops defined by each chromatin conformation capture dataset. Off-diagonal values show the number of links that overlap between the datasets. **(D)** Quantification of the relationship between the TADs and the number of putative regulatory links that overlap with the interactions derived from Hi-C[2], promoter capture Hi-C[10], H3K27ac HiChIP[11], and Micro-C[12] in the NA12878 individual. **(E)** Bar plots showing the numbers of putative allele-specific and balanced regulatory links (top panel) and the fraction of links in the same TAD (bottom panel) for each individual. Regulatory links not located within the same TAD are highlighted in black. **(F)** Histogram showing the distances between open chromatin peaks and genes comprising allele-specific and balanced regulatory links for each individual. All putative regulatory links identified within one megabase are shown. Those not located within the same TAD are highlighted in black and filtered for downstream analyses. **(G)** Quantification of concordant (grey) and discordant (white) links within allele-specific and balanced regulatory links at various distance bins for each individual. Balanced links were classified as concordant or discordant according to the sign of the non-significant log2-fold-change.

Supplementary Figure 6

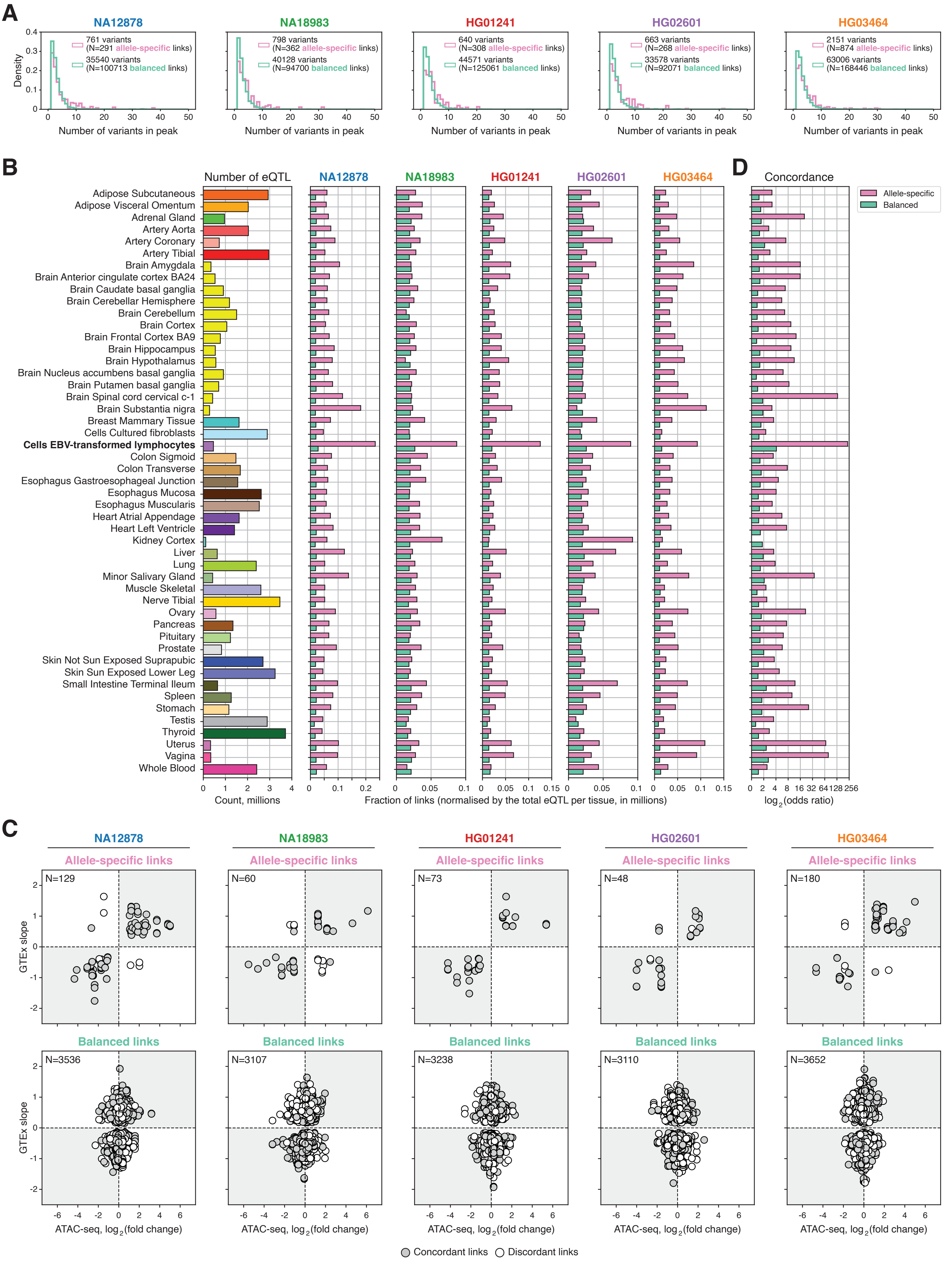

Supplementary Figure 6. Comparison of putative regulatory links with population-derived GTEx eQTL data. (A) Histograms showing the number of variants overlapping the allele-specific (in pink) and balanced (in turquoise) peaks comprising putative regulatory links for each individual. The total number of variants overlapping allele-specific and balanced peaks and the number of links these peaks are involved in is indicated in the top right corner. **(B)** Quantification of allele-specific (in pink) and balanced (in turquoise) putative regulatory links overlapping with GTEx eQTL data for all tissues[13]. The fractions of variants that overlap with GTEx eQTL associations were normalised by the total number of eQTLs detected in GTEx for each tissue. **(C)** Relationship between the effects from personal genomics and GTEx slopes for allele-specific (top panel) and balanced (bottom panel) regulatory links for each individual. Each data point represents a variant-gene regulatory link that is also an eQTL in the GTEx dataset for LCLs[13]. Links concordant and discordant with gene expression are shown in grey and white, respectively. The number of data points on each panel is indicated in the top left corner. **(D)** Concordance between the effects from personal genomics and GTEx slopes for allele-specific (pink) and balanced (turquoise) regulatory links. The odds ratios for each tissue are calculated using Fisher's exact test for combined data from all five individuals.

Supplementary Figure 7

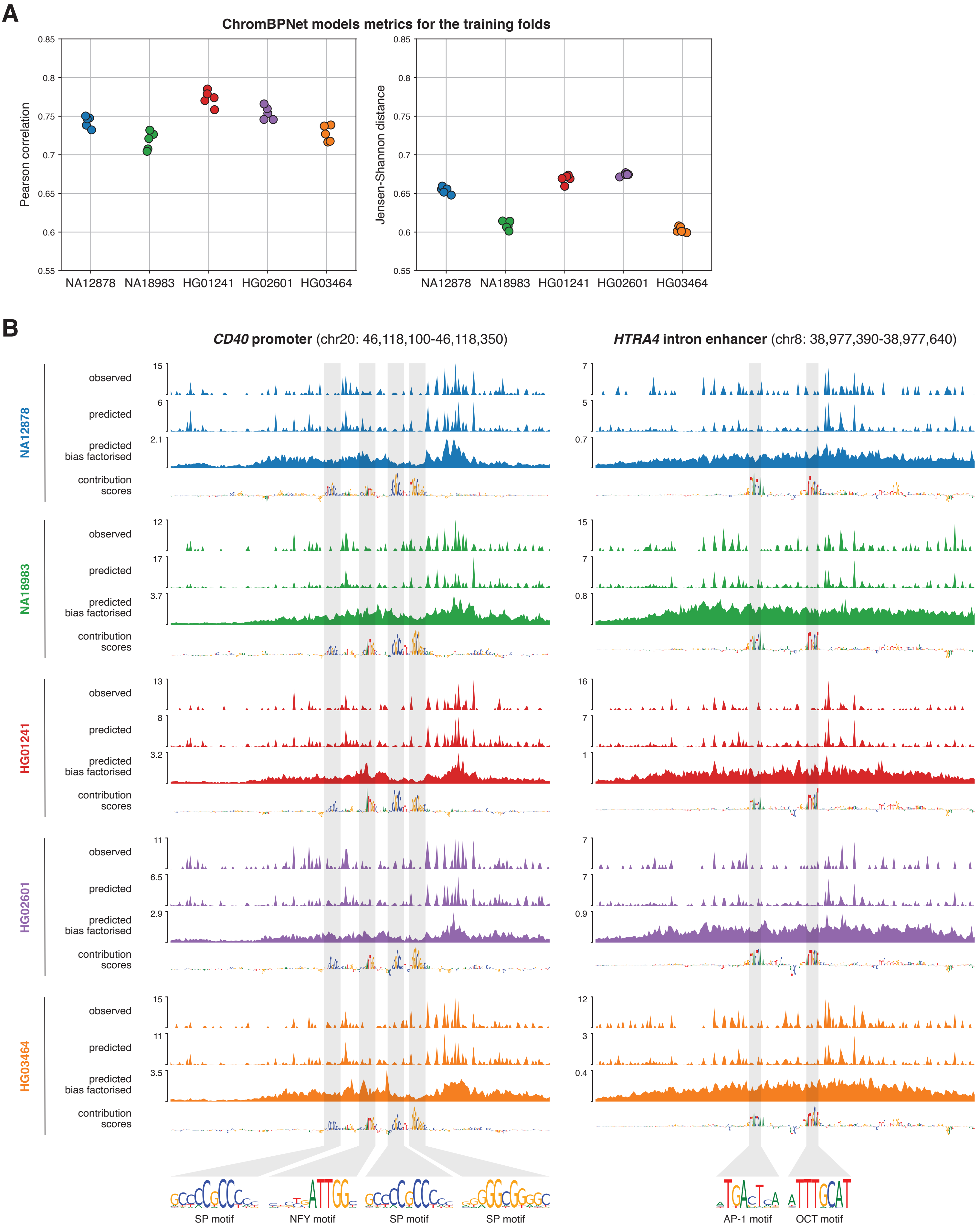

Supplementary Figure 7. Performance of the ChromBPNet deep learning models of chromatin accessibility. (A) Pearson correlation between observed and predicted total counts across ATAC-seq peaks (left panel) and Jensen-Shannon distance between the base-resolution observed and predicted profiles across ATAC-seq peaks (right panel). Each data point represents the values for a ChromBPNet model, trained on one of the five folds in the chromosome-holdout cross-validation[14]. **(B)** Genomic tracks showing, for each individual, the observed ATAC-seq profiles, the predicted ChromBPNet profiles, the predicted bias-factorised ChromBPNet profiles, and the contribution scores to the profile shape predictions for the hg38 reference human genome. *De novo* TF motifs, identified across the predicted contribution scores for each individual, are shown below. Example regions at the *CD40* promoter (left panel) and the *HTRA4* intron enhancer (right panel) are shown, located on the held-out test chromosomes 20 and 8, respectively.

Supplementary Figure 8

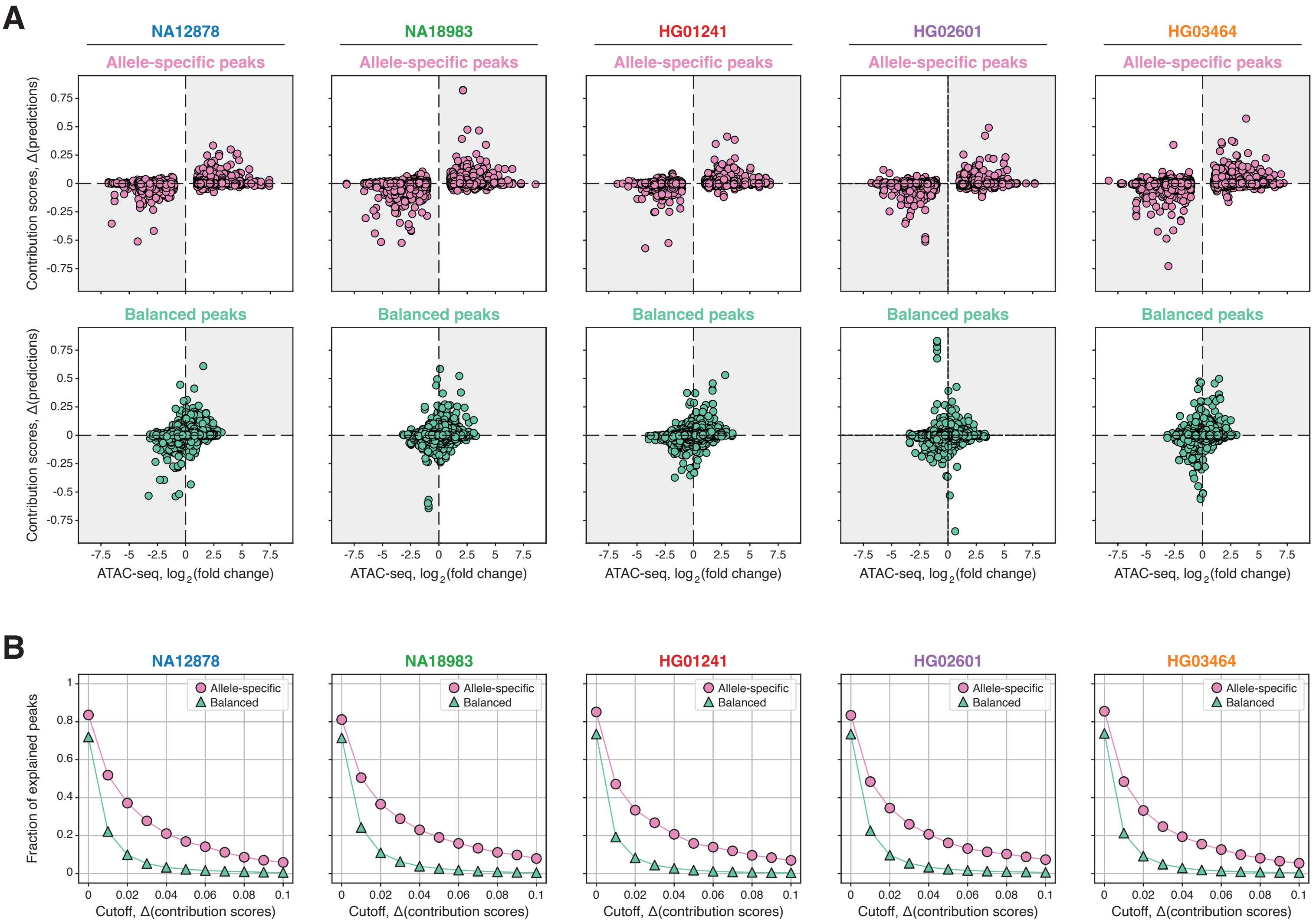

Supplementary Figure 8. Correlation between chromatin accessibility and predicted contribution score differences between alleles. (A) Scatterplots showing the relationship between measured chromatin accessibility and predicted contribution score differences between alleles for variants located in allele-specific (top row) and balanced (bottom row) open chromatin peaks for each individual. **(B)** Quantification of the ATAC-seq peaks explained by the ChromBPNet model at various contribution score differences cutoffs. A cutoff of 0.02 was used for downstream analyses involving ChromBPNet contribution scores.

Supplementary Figure 9

A

Number of allele-specific peaks with **disrupted motifs**

	AP-1	CRE	CTCF	EBF	EBOX	EGR1	ETS	HNF1	IRF	MEF2	NFI	NFKB	NFY	NRF1	OCT	PAX	RFX	RUNX	SP	TP53	YY1	ZBTB33	ZNF143
NA12878	39	7	12	6	7	8	35	1	37	0	3	25	1	1	3	1	0	39	10	0	0	1	0
NA18983	53	3	16	3	12	10	67	0	79	1	5	25	0	1	11	1	0	40	16	0	0	0	1
HG01241	8	5	17	0	7	3	26	0	42	1	0	5	1	0	14	0	1	15	8	0	0	0	0
HG02601	13	4	11	1	8	3	27	0	49	0	0	16	1	1	4	0	0	10	14	0	0	0	0
HG03464	42	6	21	3	24	7	85	0	96	4	0	27	2	2	15	7	0	36	14	0	1	0	0

B

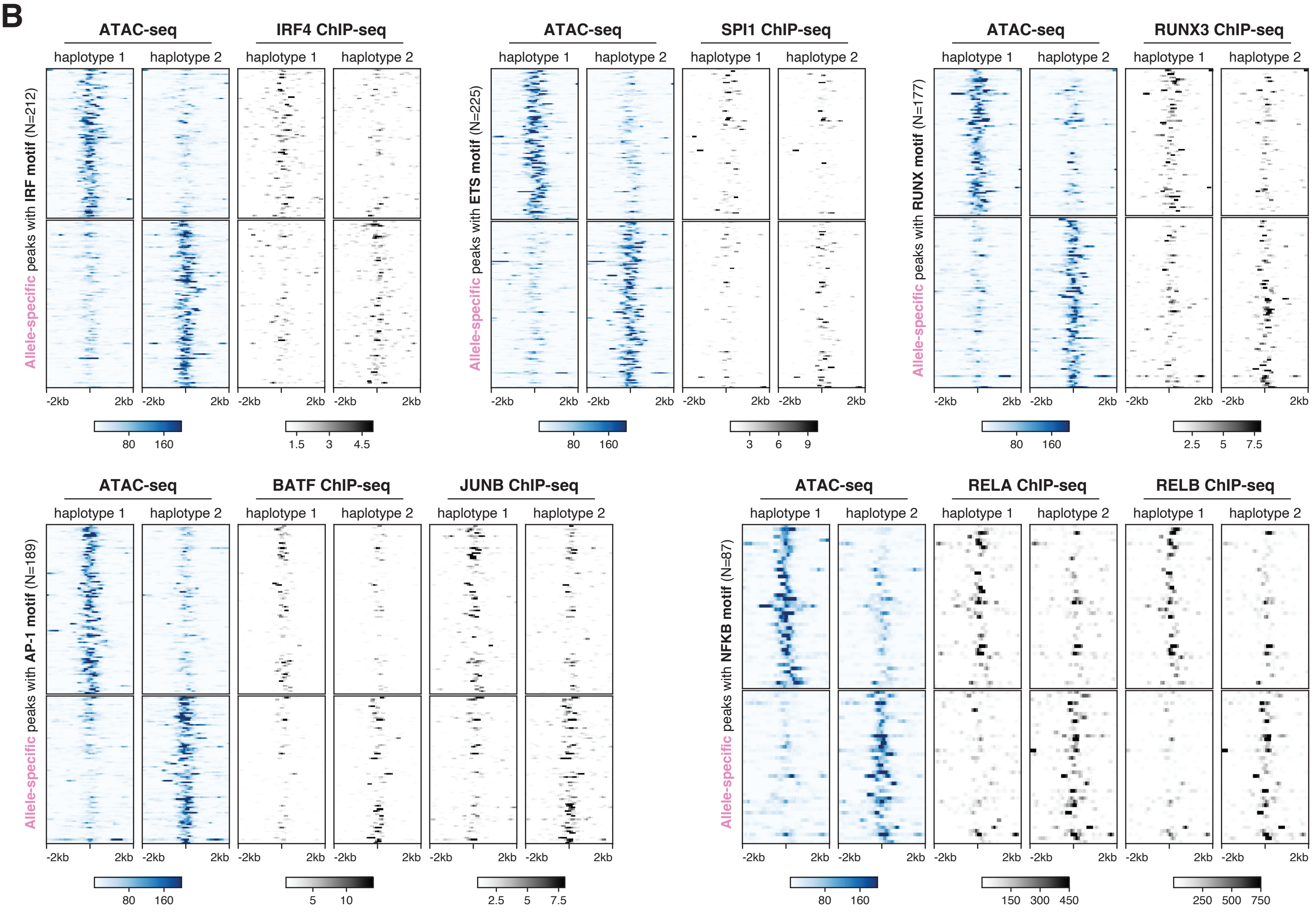

Supplementary Figure 9. Annotation of variants that disrupt transcription factor binding within allele-specific open chromatin peaks. (A) Heatmap showing the number of allele-specific peaks with annotated transcription factor motifs disruptions for each individual. **(B)** Tornado plots showing the ATAC-seq and ChIP-seq signals at the control allele-specific peaks with intact IRF, AP1, ETS, NFKB or RUNX motifs. Publicly available ChIP-seq data from the NA12878 individual were used[15,16]. The regions are sorted by predicted contribution score differences.

Supplementary Figure 10

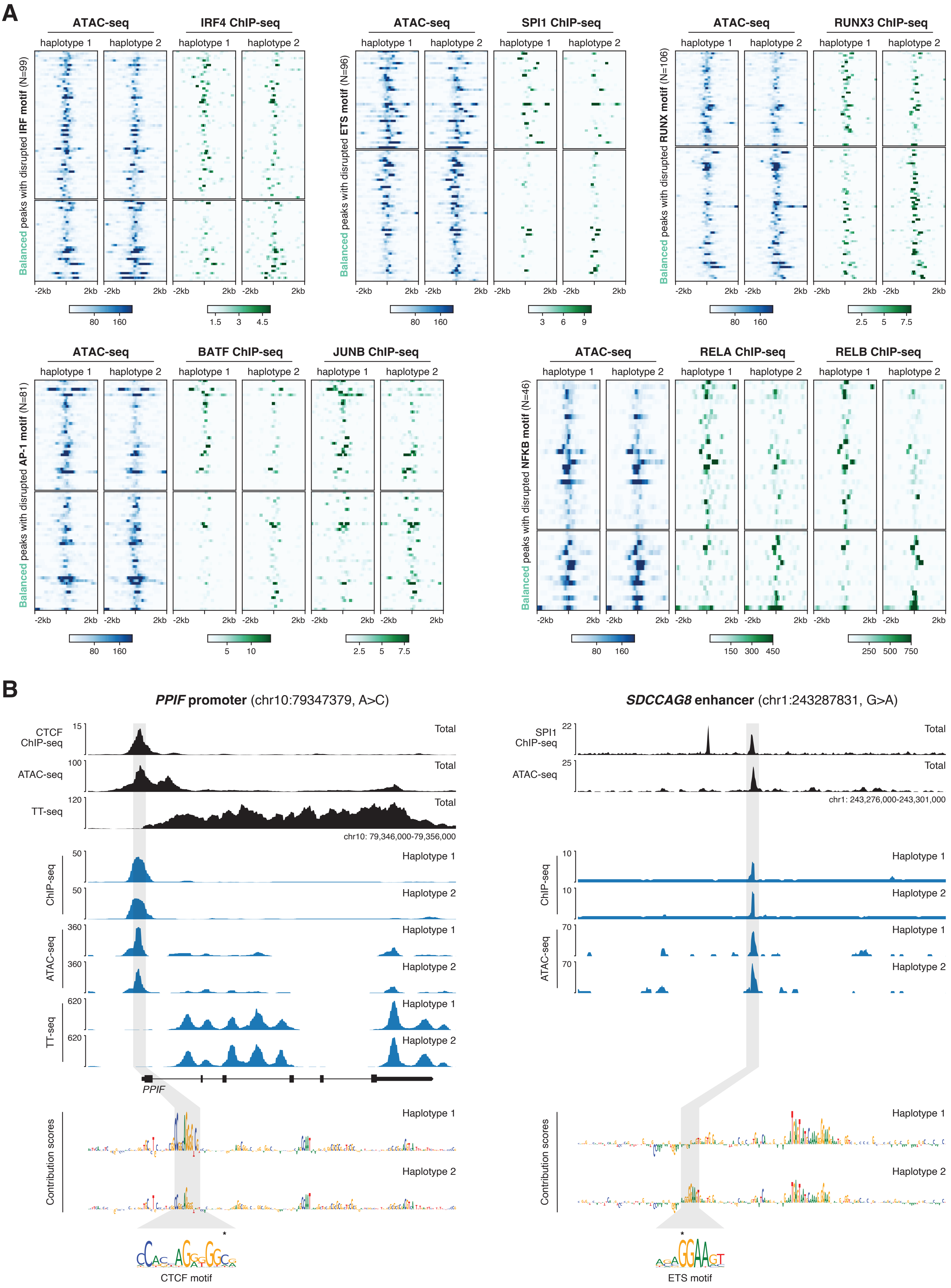

Supplementary Figure 10. Variants that disrupt transcription factor binding motifs within balanced peaks represent limitations of the deep learning model. (A) Tornado plots showing the ATAC-seq and ChIP-seq signals at the balanced peaks with disrupted IRF, AP1, ETS, NFKB or RUNX motifs. Publicly available ChIP-seq data from the NA12878 individual were used[15,16]. The regions are sorted by predicted contribution score differences. **(B)** Genomic tracks showing total and haplotype-resolved ChIP-seq, ATAC-seq, and TT-seq data, as well as predicted ChromBPNet contribution scores for personal genomes, for the *PPIF* gene (right panel) and the *SDCCAG8* intron (left panel) in the NA12878 individual. The grey rectangle indicates the position of the balanced open chromatin peak containing the CTCF and ETS motifs, respectively, which are predicted to be disrupted by the presence of the variant. The positions of the variants within the motifs are indicated by the asterisks. Publicly available CTCF and SPI1 ChIP-seq data from the NA12878 individual were used to validate differential binding[5,15].

Supplementary Figure 11

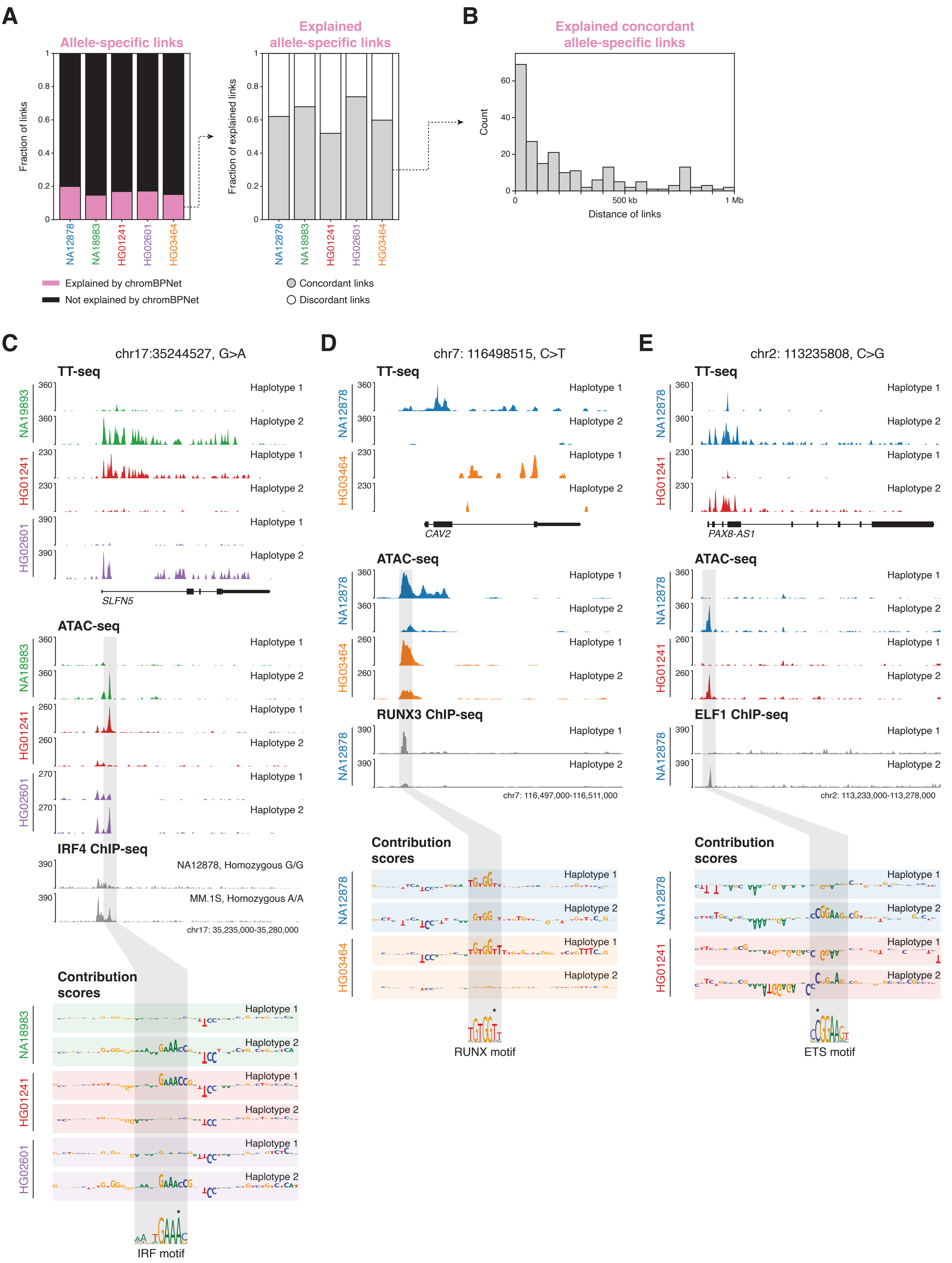

Supplementary Figure 11. Integration of allele-specific data with deep learning to pinpoint candidate causal variants and their mechanisms. (A) The fraction of allele-specific regulatory links explained by TF motif disruptions, as predicted by ChromBPNet (left panel). The fraction of concordant and discordant links within the explained allele-specific regulatory links (right panel). **(B)** Histogram showing the distances between open chromatin peaks and genes comprising the concordant allele-specific regulatory links explained by ChromBPNet. The plot shows the combined data from all five individuals. **(C)** Genomic tracks showing haplotype-resolved ATAC-seq, TT-seq, and ChIP-seq data, as well as predicted ChromBPNet contribution scores for personal genomes, for the *SLFN5* gene in the NA18983, HG01241, and HG02601 individuals. The grey rectangle indicates the position of the allele-specific open chromatin peak containing the IRF motif, which is disrupted by the presence of the variant. The position of the variant within the motif is indicated by the asterisk. Publicly available IRF4 ChIP-seq data from the NA12878 individual containing homozygous G allele and MM.1S individual containing homozygous A were used to validate differential binding[15,17]. **(D)** Genomic tracks showing haplotype-resolved ATAC-seq, TT-seq, and ChIP-seq data, as well as predicted ChromBPNet contribution scores for personal genomes, for the *CAV2* gene in the NA12878 and HG03464 individuals. The grey rectangle indicates the position of the allele-specific open chromatin peak containing the RUNX motif, which is disrupted by the presence of the variant. The position of the variant within the motif is indicated by the asterisk. Publicly available RUNX3 ChIP-seq data from the NA12878 individual was used to validate differential binding[15]. **(E)** Genomic tracks showing haplotype-resolved ATAC-seq, TT-seq, and ChIP-seq data, as well as predicted ChromBPNet contribution scores for personal genomes, for the *PAX8-AS1* gene in the NA12878 and HG01241 individuals. The grey rectangle indicates the position of the allele-specific open chromatin peak containing the ETS motif, which is disrupted by the presence of the variant. The position of the variant within the motif is indicated by the asterisk. Publicly available ELF1 ChIP-seq data from the NA12878 individual was used to validate differential binding[15].

Supplementary Figure 12

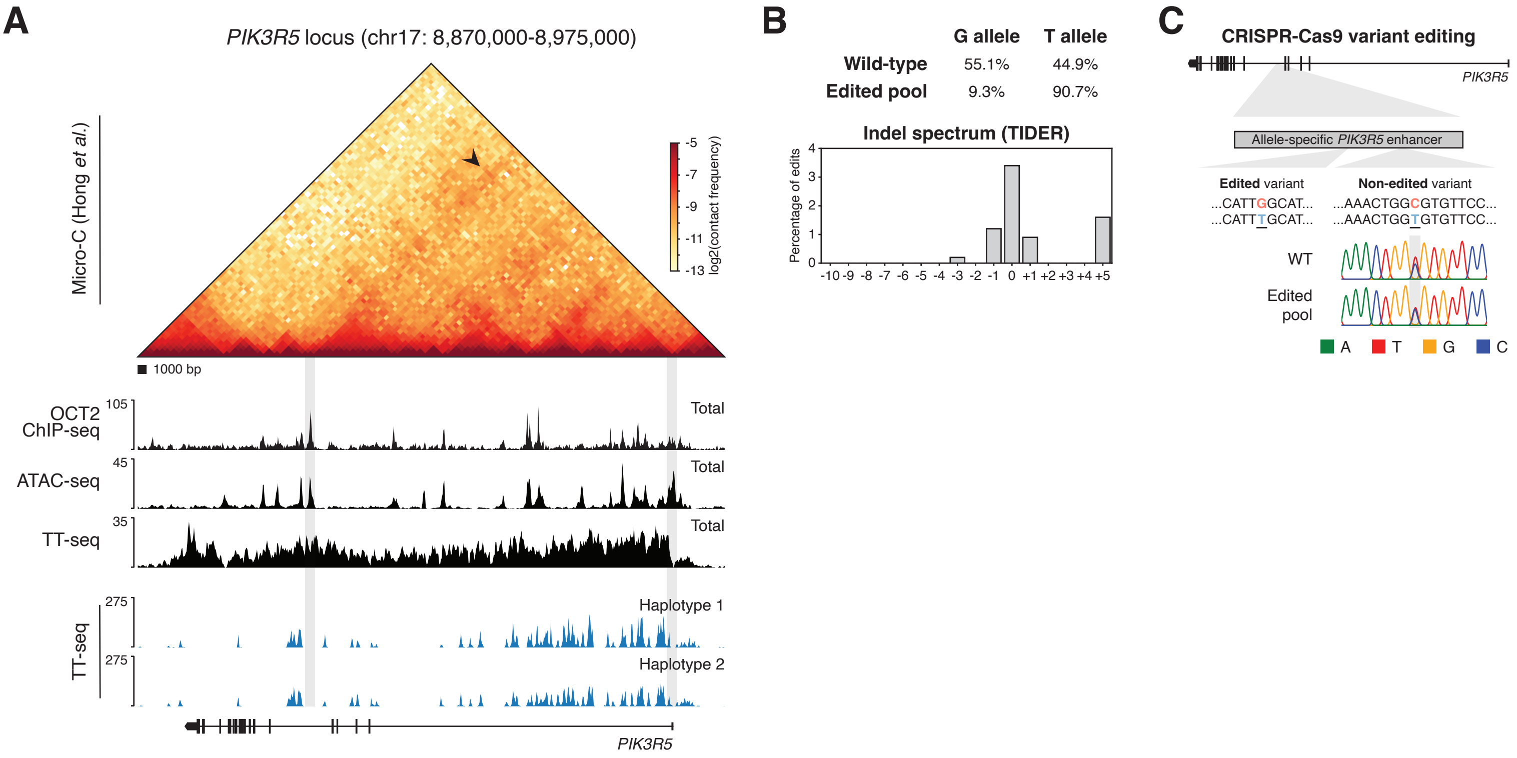

Supplementary Figure 12. *PIK3R5* gene regulation by the identified distal enhancer in the NA12878 individual. (A) A high-resolution Micro-C matrix[12] showing interaction frequencies (top panel) and genomic tracks showing OCT2 ChIP-seq, ATAC-seq and haplotype-resolved TT-seq data (bottom panels) for the *PIK3R5* gene in the NA12878 individual. Publicly available OCT2 ChIP-seq data from the NA12878 individual was used[15]. The grey rectangle on the left shows the location of the distal enhancer containing the OCT motif. This motif is disrupted by the rare variant present in the HG03464 individual but is intact in NA12878. The grey rectangle on the right indicates the position of the *PIK3R5* promoter. The interactions between the OCT2-occupied enhancer and the *PIK3R5* promoter are indicated by an arrow on the Micro-C matrix. **(B)** The fractions of G and T alleles quantified by TIDER[18] for the wild-type and the edited pool of HG03464 LCLs (top panel). The fraction of the T allele was considered to be the fraction of HDR, from which the fraction of the G allele was calculated so that their sum is equal to 1, not considering the indels fraction. Indel spectrum determined by TIDER[18] for the edited pool of HG03464 LCLs (bottom panel). **(C)** Sanger sequencing tracks of the genomic DNA for the wild-type and the edited pool showing the variants, flanking the edited variant within haplotype 1 of the allele-specific distal enhancer, in the HG03464 LCLs.

1. Chen, Z. *et al.* Ultralow-input single-tube linked-read library method enables short-read second-generation sequencing systems to routinely generate highly accurate and economical long-range sequencing information. *Genome Res* **30**, 898-909 (2020).
2. Rao, S.S. *et al.* A 3D map of the human genome at kilobase resolution reveals principles of chromatin looping. *Cell* **159**, 1665-80 (2014).
3. Wang, O. *et al.* Efficient and unique cobarcoding of second-generation sequencing reads from long DNA molecules enabling cost-effective and accurate sequencing, haplotyping, and de novo assembly. *Genome Res* **29**, 798-808 (2019).
4. Ebert, P. *et al.* Haplotype-resolved diverse human genomes and integrated analysis of structural variation. *Science* **372**(2021).
5. Kasowski, M. *et al.* Extensive variation in chromatin states across humans. *Science* **342**, 750-2 (2013).
6. Tehranchi, A. *et al.* Fine-mapping cis-regulatory variants in diverse human populations. *Elife* **8**(2019).
7. Ginjala, V. Gene imprinting gateway. *Genome Biology* **2**(2001).
8. Ashburner, M. *et al.* Gene ontology: tool for the unification of biology. The Gene Ontology Consortium. *Nat Genet* **25**, 25-9 (2000).
9. Kravitz, S.N. *et al.* Random allelic expression in the adult human body. *Cell Rep* **42**, 111945 (2023).
10. Mifsud, B. *et al.* Mapping long-range promoter contacts in human cells with high-resolution capture Hi-C. *Nat Genet* **47**, 598-606 (2015).
11. Mumbach, M.R. *et al.* Enhancer connectome in primary human cells identifies target genes of disease-associated DNA elements. *Nat Genet* **49**, 1602-1612 (2017).
12. Hong, C.K.Y., Feng, F., Ramanathan, V., Liu, J. & Hansen, A.S. Genome structure mapping with high-resolution 3D genomics and deep learning. *bioRxiv* (2025).
13. Consortium, G.T. The GTEx Consortium atlas of genetic regulatory effects across human tissues. *Science* **369**, 1318-1330 (2020).
14. Pampari, A. *et al.* ChromBPNet: bias factorized, base-resolution deep learning models of chromatin accessibility reveal cis-regulatory sequence syntax, transcription factor footprints and regulatory variants. *bioRxiv* (2025).
15. Consortium, E.P. An integrated encyclopedia of DNA elements in the human genome. *Nature* **489**, 57-74 (2012).
16. Zhao, B. *et al.* The NF-kappaB genomic landscape in lymphoblastoid B cells. *Cell Rep* **8**, 1595-606 (2014).
17. Loven, J. *et al.* Selective inhibition of tumor oncogenes by disruption of super-enhancers. *Cell* **153**, 320-34 (2013).
18. Brinkman, E.K. *et al.* Easy quantification of template-directed CRISPR/Cas9 editing. *Nucleic Acids Res* **46**, e58 (2018).
