# Supplementary material for: Mapping functional non-coding variation in individual human genomes through haplotyping, multiomics, and deep learning
Source: Nat Commun. 2026 Apr 29;17:5856. doi: 10.1038/s41467-026-72392-x (PMC13333882; doi:10.1038/s41467-026-72392-x)
Supplement: Supplementary file 2 — Description of Additional Supplementary Files [file 41467_2026_72392_MOESM2_ESM.pdf]

Description of Additional supplementary files

Supplementary Data 1: Previously published datasets used in this study.

Supplementary Data 2: Whole-chromosome haplotype phasing statistics for individual human genomes.

- Sheet Summary contains information on total number of heterozygous variants (SNPs, insertions, and deletions) phased for each sample. - Sheet Per chromosome contains information on fraction of heterozygous variants and chromosome length phased and switch and mismatch error rates per chromosome for each sample.

Supplementary Data 3: Allele-specific and balanced open chromatin peaks and genes identified for ATAC-seq and TT-seq data of each individual using the Wald test implemented in DESeq2.

- Sheets peaks_NA12878, peaks_NA18983, peaks_HG01241, peaks_HG02601, peaks_HG03464 contain DESeq2 results for allele-specific open chromatin peaks testing for each sample. - Sheets genes_NA12878, genes_NA18983, genes_HG01241, genes_HG02601, genes_HG03464 contain DESeq2 results for allele-specific gene expression testing for each sample.

Supplementary Data 4: Putative regulatory links constructed between allele-specific open chromatin peaks and genes.

- Sheets NA12878_links, NA18983_links, HG01241_links, HG02601_links, HG03464_links contain putative allele-specific regulatory links obtained by connecting allelespecific open chromatin peaks and genes over a genomic distance of one megabase for each sample.

Supplementary Data 5: Motif disruptions by variants within allele-specific open chromatin peaks identified using the predicted ChromBPNet contribution scores.

- Sheets NA12878_annotation, NA18983_annotation, HG01241_annotation, HG02601_annotation, HG03464_annotation contain annotated by ChromBPNet motif disruptions by variants within putative allele-specific regulatory links for each sample.
