# Supplementary material for: Mapping functional non-coding variation in individual human genomes through haplotyping, multiomics, and deep learning
Source: Nat Commun. 2026 Apr 29;17:5856. doi: 10.1038/s41467-026-72392-x (PMC13333882; doi:10.1038/s41467-026-72392-x)
Supplement: Supplementary file 8 — Reporting Summary [file 41467_2026_72392_MOESM8_ESM.pdf]

nature portfolio

Corresponding author(s): Elzo de Wit

Last updated by author(s): Mar 24, 2026

Reporting Summary

Nature Portfolio wishes to improve the reproducibility of the work that we publish. This form provides structure for consistency and transparency in reporting. For further information on Nature Portfolio policies, see our Editorial Policies and the Editorial Policy Checklist.

Statistics

For all statistical analyses, confirm that the following items are present in the figure legend, table legend, main text, or Methods section.

n/a	Confirmed	
☐	☒	The exact sample size (*n*) for each experimental group/condition, given as a discrete number and unit of measurement
☐	☒	A statement on whether measurements were taken from distinct samples or whether the same sample was measured repeatedly
☐	☒	The statistical test(s) used AND whether they are one- or two-sided<br>*Only common tests should be described solely by name; describe more complex techniques in the Methods section.*
☐	☒	A description of all covariates tested
☐	☒	A description of any assumptions or corrections, such as tests of normality and adjustment for multiple comparisons
☐	☒	A full description of the statistical parameters including central tendency (e.g. means) or other basic estimates (e.g. regression coefficient) AND variation (e.g. standard deviation) or associated estimates of uncertainty (e.g. confidence intervals)
☐	☒	For null hypothesis testing, the test statistic (e.g. *F*, *t*, *r*) with confidence intervals, effect sizes, degrees of freedom and *P* value noted<br>*Give P values as exact values whenever suitable.*
☒	☐	For Bayesian analysis, information on the choice of priors and Markov chain Monte Carlo settings
☒	☐	For hierarchical and complex designs, identification of the appropriate level for tests and full reporting of outcomes
☐	☒	Estimates of effect sizes (e.g. Cohen's *d*, Pearson's *r*), indicating how they were calculated

Our web collection on statistics for biologists contains articles on many of the points above.

Software and code

Policy information about availability of computer code

Data collection

No software was used for data collection.

Data analysis

BCFtools v1.9
SnpSift v4.3p
vcflib v1.0.3
vt v2015.11.10
RTG Tools v3.12.1
Long Ranger pipeline v2.2.2
bamtofastq v1.4.1
Picard v2.27.4
SAMtools v1.15, v1.6
TELLseq v0.1.3
bwa mem v0.7.17-r1188
HapCUT2 v1.3.3
BAMtools v2.5.2
minimap2 v2.24
MACS2 v2.2.6
BEDtools v2.31.1
deepTools v3.5.1
CrossMap v0.6.4
pyGenomeTracks v3.8

STAR v2.7.10a
HTSeq v0.13.5
DESeq2 v1.36.0
ChromBPNet v0.1.7
TF-MoDISco lite v2.2.0
Tomtom v4.11.2
Maxquant software v2.4.9.0
cooltools v.0.3.2
pairtools v0.3.0
cooler v0.8.11
bioframe v0.3.3
TIDER v5.0.5

For manuscripts utilizing custom algorithms or software that are central to the research but not yet described in published literature, software must be made available to editors and reviewers. We strongly encourage code deposition in a community repository (e.g. GitHub). See the Nature Portfolio guidelines for submitting code & software for further information.

Data

Policy information about availability of data

All manuscripts must include a data availability statement. This statement should provide the following information, where applicable:

- Accession codes, unique identifiers, or web links for publicly available datasets
- A description of any restrictions on data availability
- For clinical datasets or third party data, please ensure that the statement adheres to our policy

Generated linked-reads, Hi-C, ATAC-seq, and TT-seq data have been deposited at GEO database (GSE308298) and are publicly available as of the date of publication. Proteomics data have been deposited at PRIDE database (PXD069116) and are publicly available as of the date of publication. Previously published datasets used in this study are listed in Supplementary Data 1. Oligonucleotides, primers, and gRNA sequence are listed in Supplementary Table 1.

Research involving human participants, their data, or biological material

Policy information about studies with human participants or human data. See also policy information about sex, gender (identity/presentation), and sexual orientation and race, ethnicity and racism.

Reporting on sex and gender	Sex charachteristics of the lymphoblastoid cell lines were taken from the NHGRI Sample Repository for Human Genetic Research at the Coriell Institute for Medical Research and 1000 Genomes Project. Sex-based analysis of the allele-specific transcription and accessibility of the X chromosome was used during validation.
Reporting on race, ethnicity, or other socially relevant groupings	Ethnicity charachteristics of the lymphoblastoid cell lines were taken from the NHGRI Sample Repository for Human Genetic Research at the Coriell Institute for Medical Research and 1000 Genomes Project. Each superpopulation has been represented by one sample in the study.
Population characteristics	Population charachteristics of the lymphoblastoid cell lines were taken from the NHGRI Sample Repository for Human Genetic Research at the Coriell Institute for Medical Research and 1000 Genomes Project.
Recruitment	N/A
Ethics oversight	N/A

Note that full information on the approval of the study protocol must also be provided in the manuscript.

Field-specific reporting

Please select the one below that is the best fit for your research. If you are not sure, read the appropriate sections before making your selection.

☒ Life sciences ☐ Behavioural & social sciences ☐ Ecological, evolutionary & environmental sciences

For a reference copy of the document with all sections, see nature.com/documents/nr-reporting-summary-flat.pdf

Life sciences study design

All studies must disclose on these points even when the disclosure is negative.

Sample size	Five samples have been selected for this study. No sample size quantification was performed. To maximise genetic diversity, we selected select samples representing 5 distinct superpopulations: NA12878 (European), NA18983 (East Asian), HG01241 (American), HG02601 (South Asian), and HG03464 (African). All quantifications presented in the study are performed within sample in an allele-specific fashion, therefore low sample size has no effect on the presented data.
Data exclusions	No data were excluded from the analysis.
Replication	Linked-reads and Hi-C experiments were not replicated. TT-seq and ATAC-seq experiments were performed using two and three biological

Replication	replicates, respectively. ChromBPNet deep learning models were trained on 5 independent folds to assess reproducibility. All replication attempts have been successful.
Randomization	Randomisation was not relevant, since the quantifications were performed independently within each sample.
Blinding	Blinding was not relevant since no randomisation has been performed.

Reporting for specific materials, systems and methods

We require information from authors about some types of materials, experimental systems and methods used in many studies. Here, indicate whether each material, system or method listed is relevant to your study. If you are not sure if a list item applies to your research, read the appropriate section before selecting a response.

Materials & experimental systems

n/a	Involved in the study
☒	☐ Antibodies
☐	☒ Eukaryotic cell lines
☒	☐ Palaeontology and archaeology
☒	☐ Animals and other organisms
☒	☐ Clinical data
☒	☐ Dual use research of concern
☒	☐ Plants

Methods

n/a	Involved in the study
☒	☐ ChIP-seq
☒	☐ Flow cytometry
☒	☐ MRI-based neuroimaging

Eukaryotic cell lines

Policy information about cell lines and Sex and Gender in Research

Cell line source(s)	The following lymphoblastoid cell lines were obtained from the NHGRI Sample Repository for Human Genetic Research at the Coriell Institute for Medical Research: GM12878, GM18983, HG01241, HG02601, HG03464.
Authentication	None of the cell lines used were authenticated.
Mycoplasma contamination	Cells were routinely tested for Mycoplasma (Lonza, Cat. #LT07-318).
Commonly misidentified lines (See ICLAC register)	None of the cell lines used are in the ICLAC register.

Plants

Seed stocks	N/A
Novel plant genotypes	N/A
Authentication	N/A
