# Supplementary material for: Mapping functional non-coding variation in individual human genomes through haplotyping, multiomics, and deep learning
Source: Nat Commun. 2026 Apr 29;17:5856. doi: 10.1038/s41467-026-72392-x (PMC13333882; doi:10.1038/s41467-026-72392-x)
Supplement: Supplementary file 9 — Transparent Peer Review file [file 41467_2026_72392_MOESM9_ESM.pdf]

nature portfolio

Peer Review File

Mapping functional non-coding variation in individual human genomes through haplotyping, multiomics, and deep learning

Corresponding Author: Dr Elzo de Wit

This file contains all reviewer reports in order by version, followed by all author rebuttals in order by version.

Version 0:

Reviewer comments:

Reviewer #1

(Remarks to the Author)
This manuscript integrates haplotype-resolved regulatory profiling with deep learning–based variant interpretation. The overall approach is conceptually interesting and makes effective use of multi-omic datasets, and the examples presented are informative. Overall, the study represents a useful contribution to the field. At the same time, several aspects of the analysis could benefit from additional evaluation and clarification, particularly with respect to sensitivity, enhancer–promoter assignment, experimental validation, and the interpretation of deep learning predictions. Addressing these points would further strengthen the rigor and interpretability of the conclusions.

1. Sensitivity and coverage of allele-specific analyses
• What fraction of ATAC-seq peaks and transcribed genes contain sufficient heterozygous SNPs to be informative for allele-specific analysis?
• Can the authors provide genome-wide summaries of SNP coverage for both ATAC-seq and TT-seq, and discuss how this coverage impacts sensitivity and potential false-negative rates?

2. Enhancer–promoter (P–E) assignment strategy
• How do enhancer–promoter links inferred from allelic ATAC-seq and TT-seq covariation compare, at a genome-wide level, with links derived from chromatin proximity assays such as promoter capture Hi-C, HiChIP, or Micro-C?
• Given that GM12878 is a well-characterized ENCODE reference cell line, could existing chromatin interaction datasets be used to benchmark or contextualize the proposed P–E assignment method?

3. Experimental validation using Cas9-based precise editing
• What is the overall Cas9 cutting efficiency and, more specifically, the precise (HDR-mediated) editing rate in the pooled validation experiments?
• How were indel frequencies assessed, and could deep sequencing be used to report both intended edits and unintended editing outcomes?

4. Interpretation and evaluation of deep learning model predictions
• How well does ChromBPNet predict homozygous, individual-specific ATAC-seq peak gain or loss, as opposed to allelic imbalance at shared peaks?
• Large ATAC-seq fold changes are observed even for allelically balanced peaks; how is experimental variability accounted for in the model comparison?
• Given the presence of many strong allele-specific ATAC-seq signals, have the authors considered using these data to retrain or fine-tune ChromBPNet (e.g., by splitting the data into training and test sets) to capture regulatory features that may not be learned by existing models?

(Remarks on code availability)
https://doi.org/10.5281/zenodo.17285189
the link does not work for me

Reviewer #2

(Remarks to the Author)
Magnitov et al. reconstruct whole-chromosome haplotypes in several lymphoblastoid cell lines and use them to perform allele-specific analysis of chromatin accessibility and transient transcriptome. They use these results to confirm correlations between accessibility and transcription, assess enrichment of allele-specific variants for known eQTLs and use an established deep learning model to predict variant effects on TF binding. They validate one case of predicted OCT2 motif disruption by restoring the motif using DNA editing.

In principle, this study is well executed and presents an interesting application of allele-specific analysis. My questions relate to the framing, rationale and general applicability of the proposed approach.

1. To a reader outside the field, the study may create an impression - quite possibly, inadvertently - that it pioneered allele-specific variant detection and interpretation. I'm sure the authors would readily agree that this isn't the case. Statistical tools for allele-specific variant detection (such as WASP, RASQUAL, and BaseQTL) are available and widely used, and the same is true for the ChromBPNet framework used for variant effect prediction.

2. A key limitation of the pure, n=1, allele-specific analysis compared with standard genetic association testing is the problem of linkage. In the allele-specific analysis of a single individual (however well-phased), statistical fine-mapping is impossible, as the 'linkage block' is the whole cis-haplotype segment. In this context, the statement early in the Results (line 97) that "Haplotyping therefore enables attributing changes in gene expression directly to regulatory elements and the genetic variants within them" is conceptually misleading, because a direct attribution to *any* variant is, in fact, impossible in this setting. The key strength of existing approaches lies in combining intra- and inter-individual variation, which enables circumventing this problem at least partially, and which this study does not attempt. I suspect this is a major reason why the overlap with known eQTLs is rather low.

3. It was also unclear to me how much whole-genome phasing helps the authors' approach in practice, when they are mainly looking at regulatory links within 1Mb. Is it a 'nice to have' (with which I agree) or a transformative ('must have')? Getting to the bottom of this (through a quantitative assessment) is particularly important because a clear use case for allele-specific analysis is rare disease samples, where standard association testing is not feasible. However, from this practical perspective, the linked-read tech is discontinued, and Hi-C is expensive, so the authors' emphasis on the transformative benefits of whole-genome phasing limits the practical application of their approach.

4. I'm also not convinced by the statement that using ChromBPNet on fully-phased genomes completely abrogates trans-effects. Perhaps the authors meant it in a more specific context, in which case they should adjust their wording.

5. The conceptual novelty of the correlative analysis between ATAC and TT-seq signals is potentially overstated. The joint allelic effects of ATAC-QTLs and eQTLs are, by now, quite well established. When the same allele correlates positively with ATAC and a gene's expression, as is often the case, the conclusion is the same as the authors make: that active regulatory elements are often located on the same allele in proximity to active genes.

6. As a relatively minor point, I was also wondering why the authors didn't take advantage of Hi-C data to create putative regulatory links and only used distance windows in this analysis, since Hi-C is used for phasing.

(Remarks on code availability)

Reviewer #3

(Remarks to the Author)
The manuscript of Magnitov et al describes a study on an important topic. As early as in 2011 the seminal review of Nicholas J Schork group [PMID 21301473] drew attention of researchers to allele-specific expression and epigenetic events and underlined importance of genome phasing. From that time a huge progress in genome phasing has been achieved with many publications describing phasing up to the entire chromosome scale. Surprisingly, at least to my knowledge, nobody used the phased genomes to connect allele specific epigenetic events with allele specific expression at relatively large distances. The study described in the manuscript aims to fill this gap using several cell lines originating from individuals of different national backgrounds. The study has a clear and straightforward experimental design with chromatin allele specific accessibility (ASA) and allele-specific expression (ASE) assays used on five cell lines, the genomes of which were phased with the help of HiC and Tell-seq linked reads. Yet, unfortunately, the paper misses the clear conclusion, and it took me a lot of time to understand what actually has been done.

Major notes.

0. The most important is that the abstract lacks a clear punchline. What the readers can learn from phased genomes, which they did not know before?

1. The procedure of connecting allele specific chromatin accessibility with allele specific gene expression (Figure 2D) is a bit too simplified. As far as I understand, the authors rely upon co-occurrence of ASA and ASE events (or balanced events) in some window. Why the authors did not even try to confirm these events with HiC data they used to phase the genome? This is especially relevant for distant regulation, even a small number of such examples would be very convincing. I understand that Hi-C data usually poor in quality, but as far as I understand in many cases they can show distant

intrachromosomal contacts at least approximately. As it is difficult to distinguish the balanced and distance regulation at a larger distance (Figure 2E), a demonstration of an intrachromosomal contact may help (at least in theory).

1a. The authors claim that there approach can distinguish cis and trans interactions, but the proximity based linking they use clearly works only for distances of less than 50Kbp. For longer distances the authors mention 'spurious links to non-target genes', which are essentially the trans effects. Is it possible to evaluate somehow the percentage of false positives/trans effects for long distance links?

1b. Is there any difference in ChromBPNet contribution scores for discordant ASA peaks? Figure 2H shows many discordant ATAC-Seq peaks (white circles in the 2nd and 4th quadrants). Did you try to identify ChromBPNet motifs specifically for those peaks? Could such motifs relate to the repressor proteins?

2. The results on motif contributions obtained with the help of ChromBPNet (Figure 3) is done within a short 50bp window under a single ATAC-seq peak. Apart from four examples (PPIF in Fig. S10B, which is a negative example as the TT-Seq tracks appear to be identical for both haplotypes; SLFN5 in Fig. S11A; CAV2 in Fig. S11B; PAX8-AS1 in Fig. S11C; and PIK3R5) the result on differential motif contributions are not connected to the differential allele-specific expression. Most of the paragraph looks as an independent study on differential TF binding. What is the statistics of differential TF-binding with differential ASA and ASE? The authors give an idea that this dependence is not simple but do not give any numbers. What is the percentage of cases in which the concordant allele specific expression can be related to a motif disruption? It will be very instructive to see more examples of the kind and in what percentage of cases a differential motif contribution is associated/not associated with differential transcription activity.

2a. By the way, why the authors do not use the 'variant-scorer' function suggested by the authors of ChromBPNet but instead use difference in contribution scores in the 50bp window centred at SNP? Some justification is needed.

3. Figure 2E implies that simple, window based interaction procedure gives a single ASE-ASA linkage even for large windows. Is this one-to-one ratio true even for large windows, close to 1M in length? If not, is it possible to give a figure of the expected number of ASAs for a single ASE and vice versa, as a function on a window length?

I'm not a native English speaker, so it is difficult for me to evaluate the language quality but the paper reads smoothly.

(Remarks on code availability)
I didn't go deeply into the code, just checked it was submitted and up to the point.

Reviewer #4

(Remarks to the Author)
A fundamental challenge of genomics is identifying how non-coding regions of the genome regulate gene expression, and especially how genetic variants in these regions can influence gene expression. While GWAS and eQTL studies can be used to identify these associations across large cohorts, methods are lacking to identify these relationships from small numbers of samples. Here the authors demonstrate how they can link specific genomic variants with perturbations in expression of nearby genes using individual human genomes. Their approach relies on integration of whole-chromosome haplotyping with data on chromatin accessibility and transcription. Using a deep learning model, the authors link specific variants to disrupted transcription factor binding motifs. Finally, as a demonstration of the utility of their approach, they investigate a novel variant that disrupts an OCT2 binding side and alters expression of PIK3R5.

Overall, I think the authors have taken an original approach to address an important problem in genomics. The applicability of their method to identifying regulatory relationships for rare variants is especially compelling to me. And I think their manuscript does a good job of describing how they did this work. While I have a few minor comments and questions listed below, I think this work will be well suited to publication in Nature Communications.

MAIN POINTS

1. A key assumption of the authors' approach is that allele-specific gene expression and chromatin accessibility are both sufficiently rare across the genome that when they are found in close proximity to one another, they are likely to be related. This is certainly reasonable, and the authors do a good job of showing that their allele-specific links are much more likely to be near each other (relative to balanced links) and that concordant allele-specific links are near each other. Both of these arguments build a case that these links tend to connect features that are truly related, though as the authors acknowledge, spurious links are nevertheless present in the dataset at some rate. This point probably warrants a bit more discussion. While estimating a rigorous FDR probably isn't feasible, can the others estimate any bounds for the fraction of spurious links that might be present? In addition, while the authors emphasize the enrichment for short-distance links among the allele-specific links, the concordant/discordant fraction seems to suggest that a fair number of these longer-distance links represent real relationships as well.

2. In the legend to Figure 1D, the authors note that sometimes "genes and peaks may be involved in multiple links", and this is shown further in Figure S5B. I'm curious about allele-specific peaks that link to multiple genes, and allele-specific genes that link to multiple peaks. There are actually more of these than I anticipated. Are there any interesting biological examples among these? To what extent do you think these represent real versus coincidental links? How do their numbers change if

you tighten the distance constraints so that you're looking at a lower fraction of potentially spurious links as suggested by figure 2E?

MINOR POINTS

3. Figure S5B: typo in panel heading.

4. Figure 2E: The "concordant/discordant" fraction might initially seem a bit weird for the "balanced" case since they by definition don't show a significant bias. It might be worth mentioning in the figure legend that no significant bias was detected for these, though they were classified according to the sign of the non-significant difference between them.

(Remarks on code availability)
I verified that the code is available as promised, and it appears to be organized in a thoughtful way. It appears that there is sufficient detail to run the code, though I didn't actually try to run the code myself.

Version 1:

Reviewer comments:

Reviewer #1

(Remarks to the Author)
The authors have addressed my pervious questions.

(Remarks on code availability)

Reviewer #2

(Remarks to the Author)
I thank the authors for their work on addressing my comments.

I have one outstanding concern: point 2 in my original review, which is echoed by Reviewer 3's point 1 and Reviewer 4 point 1. I feel the authors should make it more explicit in the text that their analysis inherently cannot fine-map associations, and therefore all analyses are based on the assumed (rather than inferred) links between the genotypes and molecular phenotypes in their vicinity. Can this be stated clearly in both Results and Discussion?

Minor point: In this context, I wonder if the terminology cis- vs trans-effects is also potentially confusing, because it may be misunderstood as referring to proximal vs distal effects rather than cis-chromosomal and trans-chromosomal. It probably makes sense to amend this accordingly.

(Remarks on code availability)

Reviewer #3

(Remarks to the Author)
The new version of the manuscript is much better. In general, the manuscript is publishable in its current form, I have a small number of minor comments.

1. Page 2. Para 2. "In addition, context-specific regulatory effects can be overlooked in bulk tissues". Probably, the genetic context is discussed. I'm not sure, why the bulk tissue is important here.

2. The same page 2. "More recently, machine learning models [...] emerged." Please check the grammar. I'm not sure what has emerged here, the machine learning models or the DNA sequence.

3. Page 6. Fig. 2E. In fact the number of concordant, putative regulatory, allele specific links found in the same TAD is pretty visible up to 250kb. This is a new and interesting result, which agrees well with LD decaying distance. It is worth to mention this in the text, can be the first estimate of the kind.

I'm satisfied with the authors' answers to my questions in my first review.

(Remarks on code availability)
I have looked through the code, it looks reasonable and up to the point. I didn't do any particular code review.

Reviewer #4

(Remarks to the Author)

I'm satisfied with the authors' responses to my comments.

(Remarks on code availability)

Open Access This Peer Review File is licensed under a Creative Commons Attribution 4.0 International License, which permits use, sharing, adaptation, distribution and reproduction in any medium or format, as long as you give appropriate credit to the original author(s) and the source, provide a link to the Creative Commons license, and indicate if changes were made.
In cases where reviewers are anonymous, credit should be given to 'Anonymous Referee' and the source.
The images or other third party material in this Peer Review File are included in the article’s Creative Commons license, unless indicated otherwise in a credit line to the material. If material is not included in the article’s Creative Commons license and your intended use is not permitted by statutory regulation or exceeds the permitted use, you will need to obtain permission directly from the copyright holder.
To view a copy of this license, visit https://creativecommons.org/licenses/by/4.0/

Reviewer #1 (Remarks to the Author):

This manuscript integrates haplotype-resolved regulatory profiling with deep learning–based variant interpretation. The overall approach is conceptually interesting and makes effective use of multi-omic datasets, and the examples presented are informative. Overall, the study represents a useful contribution to the field. At the same time, several aspects of the analysis could benefit from additional evaluation and clarification, particularly with respect to sensitivity, enhancer–promoter assignment, experimental validation, and the interpretation of deep learning predictions. Addressing these points would further strengthen the rigor and interpretability of the conclusions.

Response:

We thank the reviewer for the positive comments on our work.

1. Sensitivity and coverage of allele-specific analyses

• What fraction of ATAC-seq peaks and transcribed genes contain sufficient heterozygous SNPs to be informative for allele-specific analysis?

• Can the authors provide genome-wide summaries of SNP coverage for both ATAC-seq and TT-seq, and discuss how this coverage impacts sensitivity and potential false-negative rates?

Response:

We have quantified the numbers of informative peaks and genes by overlapping them with phased heterozygous variants and calculated the coverages. While many existing papers are based on per-variant testing of allele specificity, here we leverage whole-chromosome haplotypes to aggregate the data across multiple variants within peaks/genes thus increasing our coverage per tested feature. The percentages of informative peaks and genes vary between 34.4-55.7% and 78.4-89.4%, respectively. The median allelic coverage of informative peaks and genes ranged between 7-15 and 31-68 reads per replicate, respectively. We have included these results in the updated Figure S3 and described them in the revised manuscript.

2. Enhancer–promoter (P–E) assignment strategy

• How do enhancer–promoter links inferred from allelic ATAC-seq and TT-seq covariation compare, at a genome-wide level, with links derived from chromatin proximity assays such as promoter capture Hi-C, HiChIP, or Micro-C?

• Given that GM12878 is a well-characterized ENCODE reference cell line, could existing chromatin interaction datasets be used to benchmark or contextualize the proposed P–E assignment method?

Response:

We compared the links identified in our study with the chromatin loops that were identified for the GM12878 cell line using the following methods: Hi-C, promoter capture Hi-C, H3K27ac HiChIP, and Micro-C. Of the allele-specific and balanced regulatory links, 16.4% and 22.2% respectively overlapped with chromatin loops from at least one assay (Figure S5C). While overlap with a chromatin loop can be informative, it is not a prerequisite for a regulatory element to be functionally relevant to a gene (PMID: 30478436, 36471071, 31358994, 31925403). Conversely, topologically associating domains (TADs) offer a more flexible approach to identifying relevant enhancers and promoters (PMID: 27826147, 31654669). Consistent with this, the majority of regulatory links within chromatin loops were

found to be within the same TAD. The use of TAD boundaries as an additional constraint primarily reduced the amount of long-range links, many of which are composed of peaks and genes involved in multiple links. Notably, it has not affected any results presented in the original version of the manuscript. These results are now described in the revised manuscript and included in the revised Figures 2 and S5.

3. Experimental validation using Cas9-based precise editing

• What is the overall Cas9 cutting efficiency and, more specifically, the precise (HDR-mediated) editing rate in the pooled validation experiments?

• How were indel frequencies assessed, and could deep sequencing be used to report both intended edits and unintended editing outcomes?

Response:

To answer this question, we used TIDER (PMID: 29538768) to quantify template-directed CRISPR/Cas9 editing. The estimated HDR-mediated editing efficiency was found to be 83.1%. TIDER also estimates the fraction of indels, of which there are ~7.3%, predominantly between positions -1 and +1. These results are described in the manuscript and in the revised Figure S12B. Deep sequencing (e.g. amplicon sequencing) could also be used for genotyping the edits, which is especially useful for genotyping large insertions or deletions (see: PMID: 37691488). However, based on TIDER's results, we opted for a simpler and more cost-effective approach.

4. Interpretation and evaluation of deep learning model predictions

• How well does ChromBPNet predict homozygous, individual-specific ATAC-seq peak gain or loss, as opposed to allelic imbalance at shared peaks?

Response:

As this study focused on within-individual, allele-specific heterogeneity, ChromBPNet's ability to interpret ATAC-seq peaks was not evaluated. We agree with the reviewer that the presence of homozygous variants introduces additional regulatory complexity and may be associated with gained or lost chromatin accessibility in individuals from different populations. However, the quantification of differentially accessible ATAC-seq peaks between individuals shows that 58.8-66.0% of these peaks do not contain any homozygous variants. Therefore, we believe that the majority of these peaks reflect a combination of factors beyond genetic variation, including epigenetic and clonal differences between EBV-infected cells. These are precisely the trans-acting effects that we are trying to eliminate through allele-specific analyses in this study.

• Large ATAC-seq fold changes are observed even for allelically balanced peaks; how is experimental variability accounted for in the model comparison?

Response:

We believe that large fold changes at allelic balance peaks predominantly reflect experimental variability between ATAC-seq replicates, but they may also arise due to cis-regulatory effects that are not classified as allele-specific due to statistical thresholding. To account for this variability, ChromBPNet models have been trained using merged data from three biological replicates, incorporating a bias-factorisation model for each sample. As demonstrated in the original ChromBPNet study, incorporating a bias model corrects for Tn5 cutting efficiency and, in principle, compensates for experimental variability (PMID: 39829783). In the same study, the authors also demonstrate that contribution scores derived from bias-factorised ChromBPNet models are more stable across different sequencing

depths and experimental setups (e.g. ATAC-seq vs. DNaseI-seq). For these reasons, we used predicted contribution score differences rather than total coverage for comparisons with log2-fold-changes of experimental data. Together with the corresponding bias modelling, this should account for experimental variability in our comparisons.

• Given the presence of many strong allele-specific ATAC-seq signals, have the authors considered using these data to retrain or fine-tune ChromBPNet (e.g., by splitting the data into training and test sets) to capture regulatory features that may not be learned by existing models?
Response:
We agree that allele-specific ATAC-seq data represents a promising resource for retraining or fine-tuning sequence-based deep learning models. However, the primary objective of this study was to interpret allele-specific differences using ChromBPNet predictions as a general-purpose model, rather than optimising its predictive performance. Retraining or fine-tuning the ChromBPNet model using the same signals would complicate the interpretation of the outputs and raise concerns about overfitting in our study setup. Therefore, to prioritise biological interpretation, we opted for the most general ChromBPNet use case and removed any peaks demonstrating allelic imbalance prior to training. We have added the following discussion on potential refinements to deep learning models: "*These issues could be potentially overcome by fine-tuning the existing models (e.g. by using allelic signals or complementary regulatory features), training models on larger genomic windows, and integrating predictions generated by multiple models*".

Reviewer #2 (Remarks to the Author):

Magnitov et al. reconstruct whole-chromosome haplotypes in several lymphoblastoid cell lines and use them to perform allele-specific analysis of chromatin accessibility and transient transcriptome. They use these results to confirm correlations between accessibility and transcription, assess enrichment of allele-specific variants for known eQTLs and use an established deep learning model to predict variant effects on TF binding. They validate one case of predicted OCT2 motif disruption by restoring the motif using DNA editing.
In principle, this study is well executed and presents an interesting application of allele-specific analysis. My questions relate to the framing, rationale and general applicability of the proposed approach.
Response:
We thank the reviewer for their positive attitude towards our study.

1. To a reader outside the field, the study may create an impression - quite possibly, inadvertently - that it pioneered allele-specific variant detection and interpretation. I'm sure the authors would readily agree that this isn't the case. Statistical tools for allele-specific variant detection (such as WASP, RASQUAL, and BaseQTL) are available and widely used, and the same is true for the ChromBPNet framework used for variant effect prediction.
Response:
As noted by the reviewer, we are indeed not the first to employ allele-specific analyses for variant function interpretation. To better acknowledge previous work, we have added the following sentence and cited the relevant work in the beginning of the paragraph describing our allele-specific analyses: "*Genetic variants driving allele-specific imbalance of regulatory elements activity and gene expression have been previously identified at the population level*

in a number of previous studies (PMID: 21811232, 26502339, 27089393, 32912332, 33980847, 26366987, 26656845, 34993494, 40759060)." To our knowledge, however, we are the first to use ChromBPNet for variant effect prediction in an internally controlled personal genomics setting to understand allele-specific effects of genetic variants.

2. A key limitation of the pure, n=1, allele-specific analysis compared with standard genetic association testing is the problem of linkage. In the allele-specific analysis of a single individual (however well-phased), statistical fine-mapping is impossible, as the 'linkage block' is the whole cis-haplotype segment. In this context, the statement early in the Results (line 97) that "Haplotyping therefore enables attributing changes in gene expression directly to regulatory elements and the genetic variants within them" is conceptually misleading, because a direct attribution to *any* variant is, in fact, impossible in this setting. The key strength of existing approaches lies in combining intra- and inter-individual variation, which enables circumventing this problem at least partially, and which this study does not attempt. I suspect this is a major reason why the overlap with known eQTLs is rather low.

Response:

We agree with the reviewer that statistical fine-mapping and eQTL analyses are impossible in studies with n=1 that examine intra-individual variation. The strength of an intra-individual approach lies in allele-specific analyses, which enable cis-linked regulatory potential to be assessed. Additionally, this approach enables the evaluation of low-frequency and rare variants, which fine-mapping and eQTL studies utilising inter-individual variability cannot achieve. This is a key benefit of our framework. To clarify how intra-individual analyses delineate cis and trans factors, we rephrased the confusing statement: "*Understanding how genetic variants influence gene expression requires separating cis- from trans-acting effects. Combining haplotyping, which allocates variants to their respective alleles, with allele-specific analyses reduces trans-acting confounders and provides a clearer view of the impact of cis-linked regulatory variation. Therefore, at the level of single individuals, it becomes feasible to attribute changes in gene expression to regulatory elements and the genetic variants within them (PMID: 21301473)*".

3. It was also unclear to me how much whole-genome phasing helps the authors' approach in practice, when they are mainly looking at regulatory links within 1Mb. Is it a 'nice to have' (with which I agree) or a transformative ('must have')? Getting to the bottom of this (through a quantitative assessment) is particularly important because a clear use case for allele-specific analysis is rare disease samples, where standard association testing is not feasible. However, from this practical perspective, the linked-read tech is discontinued, and Hi-C is expensive, so the authors' emphasis on the transformative benefits of whole-genome phasing limits the practical application of their approach.

Response:

While 10X linked-reads are no longer available, two other commercial linked-read assays, TELL-seq and stLFR, are accessible and can substitute 10X linked-reads (Figure S1). As we also showed in the simulation experiments, having low 3-5X coverage Hi-C data, which is not expensive, is enough to create high-quality haplotypes (Figure S1). Furthermore, alternative approaches, such as PacBio and Nanopore, can be employed with or without Hi-C data to facilitate whole-chromosome phasing or de novo haplotype assembly. While our framework may still be applicable in the absence of whole-chromosome phasing data, establishing a direct relationship between accessibility and expression for a single individual would be much more challenging. We have revised the Discussion to emphasise these

points to the reader: "*As the presented framework is a proof of concept, we started with previously obtained phased variants and progressed to a functional analysis of allele-specific expression and accessibility. It is important to note that, since all cells within an individual share the same genetic code, only one round of haplotyping or assembly is required to later evaluate the functional impact of genetic variants in any cell type. In this study, we used reference-based whole-chromosome haplotype reconstruction with 10X linked-reads and Hi-C data. Although several other linked-read technologies are available (PMID: 32540955, 30940689), using long-read sequencing technologies from Pacific Biosciences (PacBio) and Oxford Nanopore Technologies (ONT) might be more relevant in a clinical setting (PMID: 40138663, 39228712, 39862869, bioRxiv: 10.64898/2026.01.13.26343759). These technologies would additionally enable assessment of genetic variation beyond heterozygous SNPs and indels, including complex structural variants, repeat expansions and transposable element insertions, all of which may be clinically relevant (PMID: 31747936, 39825153). Long reads can then be combined with Hi-C to provide whole-chromosome phasing, or used directly for de novo variant identification and haplotype assembly (PMID: 33845884). Together, these considerations enhance the potential of haplotype-resolved genomics to bridge the gap between individual genetic variation and cell-type-specific regulatory landscapes*".

4. I'm also not convinced by the statement that using ChromBPNet on fully-phased genomes completely abrogates trans-effects. Perhaps the authors meant it in a more specific context, in which case they should adjust their wording.
Response:
We believe that the reviewer is referring to the following sentences: "*In contrast to previous studies, our reconstructed whole-chromosome haplotypes provide an ideal, internally controlled setup for evaluating the impact of genetic variants independent of trans-acting effects. This enabled us to directly evaluate the performance of the ChromBPNet models on data from the endogenous genomic context*". Unlike eQTL studies that compare gene expression changes across a panel of individuals, in our study we compare two alleles of the same individual that should not be influenced by trans-factors. This enables us to evaluate the ability of ChromBPNet models to explain allele-specific chromatin accessibility, which we have quantified experimentally using our whole-chromosome haplotypes incorporating cis-acting genetic variation. We clarify this statement in the revised manuscript: "*Our reconstructed whole-chromosome haplotypes provide an ideal, internally controlled setup for evaluating the performance of ChromBPNet models when assessing the impact of genetic variants using allele-specific data from the endogenous genomic context*".

5. The conceptual novelty of the correlative analysis between ATAC and TT-seq signals is potentially overstated. The joint allelic effects of ATAC-QTLs and eQTLs are, by now, quite well established. When the same allele correlates positively with ATAC and a gene's expression, as is often the case, the conclusion is the same as the authors make: that active regulatory elements are often located on the same allele in proximity to active genes.
Response:
Our conceptual advance is that we can do linking of ATAC-seq and TT-seq signals in single individuals, where joint QTL analyses are not possible. We changed the description of our conclusions to acknowledge the previous work on population-derived QTLs: "*Our results therefore indicate that a substantial proportion of active regulatory elements is located on the*

same allele in proximity to active genes, consistent with earlier population-based studies (PMID: 24136355, 29988122, 30478436, 39870618, 39986281).”

6. As a relatively minor point, I was also wondering why the authors didn't take advantage of Hi-C data to create putative regulatory links and only used distance windows in this analysis, since Hi-C is used for phasing.

Response:

In the revised version of the manuscript, we compared the links identified in our study with the chromatin loops that were identified for the GM12878 cell line using the following methods: Hi-C, promoter capture Hi-C, H3K27ac HiChIP, and Micro-C. The majority of regulatory links within chromatin loops were also found to be within the same topologically associating domain (TAD), which is a less conservative constraint for associating enhancers and promoters. Therefore, we decided to use TAD boundaries as a proxy for the limits of functional interactions, requiring the putative regulatory links to additionally be located within the same TAD. These results are now described in the manuscript and are included in the revised Figures 2 and S5.

Reviewer #3 (Remarks to the Author):

The manuscript of Magnitov et al describes a study on an important topic. As early as in 2011 the seminal review of Nicholas J Schork group [PMID 21301473] drew attention of researchers to allele-specific expression and epigenetic events and underlined importance of genome phasing. From that time a huge progress in genome phasing has been achieved with many publications describing phasing up to the entire chromosome scale. Surprisingly, at least to my knowledge, nobody used the phased genomes to connect allele specific epigenetic events with allele specific expression at relatively large distances. The study described in the manuscript aims to fill this gap using several cell lines originating from individuals of different national backgrounds. The study has a clear and straightforward experimental design with chromatin allele specific accessibility (ASA) and allele-specific expression (ASE) assays used on five cell lines, the genomes of which were phased with the help of HiC and Tell-seq linked reads. Yet, unfortunately, the paper misses the clear conclusion, and it took me a lot of time to understand what actually has been done.

Response:

We thank the reviewer for their positive comments on our work and the appreciation of our study design.

Major notes.

0. The most important is that the abstract lacks a clear punchline. What the readers can learn from phased genomes, which they did not know before?

Response:

We acknowledge the reviewers' concern that the phased genomes were not mentioned in the current version of the manuscript. We have now explained the benefit of doing haplotyping in the abstract a bit better by saying: “*Leveraging whole-chromosome haplotypes and allele-specific analyses, we establish regulatory links within individual human genomes, enabling us to evaluate functional consequences of both common and rare variants*”.

1. The procedure of connecting allele specific chromatin accessibility with allele specific gene expression (Figure 2D) is a bit too simplified. As far as I understand, the authors rely upon co-occurrence of ASA and ASE events (or balanced events) in some window. Why the authors did not even try to confirm these events with HiC data they used to phase the genome? This is especially relevant for distant regulation, even a small number of such examples would be very convincing. I understand that Hi-C data usually poor in quality, but as far as I understand in many cases they can show distant intrachromosomal contacts at least approximately. As it is difficult to distinguish the balanced and distance regulation at a larger distance (Figure 2E), a demonstration of an intrachromosomal contact may help (at least in theory).

Response:

We agree with the reviewer that the process of linking allele-specific chromatin accessibility and gene expression could be improved by adding Hi-C data. In the revised manuscript, we (i) contextualised our linking strategy using multiple GM12878 chromatin capture datasets: Hi-C, promoter capture Hi-C, H3K27ac HiChIP, and Micro-C, and (ii) improved our linking approach by requiring the putative regulatory links to additionally be located within the same TAD. Our data suggest that TAD boundaries as an additional constraint primarily reduce the amounts of long-range links, many of which are composed of peaks and genes involved in multiple links. These results are extensively described in the revised manuscript and in the updated Figures 2 and S5.

1a. The authors claim that there approach can distinguish cis and trans interactions, but the proximity based linking they use clearly works only for distances of less than 50Kbp. For longer distances the authors mention 'spurious links to non-target genes', which are essentially the trans effects. Is it possible to evaluate somehow the percentage of false positives/trans effects for long distance links?

Response:

As outlined in the original manuscript, our framework is not restricted to distances of less than 50 kb. The *TBC1D4* and *PIK3R5* examples shown in Figure 4 represent long-range links: 181.8 kb and 64.7 kb, respectively. We have now explicitly included the distances for the examples in the manuscript to clarify this for future readers. However, it is not possible to quantify the exact number of false positives/trans effects for long-distance links, since the true set of all functional enhancer-promoter links in the cell is unknown. One way to address this is to use additional strategies to prioritise links. As we demonstrate in the revised version of the manuscript, using TAD boundaries is one way of obtaining a cleaner set of regulatory links by considering only those within the same TAD. Another strategy, as shown in the response to *Question 3*, is to use a more conservative window when constructing regulatory links.

1b. Is there any difference in ChromBPNet contribution scores for discordant ASA peaks? Figure 2H shows many discordant ATAC-Seq peaks (white circles in the 2nd and 4th quadrants). Did you try to identify ChromBPNet motifs specifically for those peaks? Could such motifs relate to the repressor proteins?

Response:

To address this issue, we examined the chromBPNet predictions for variants found within peaks associated with discordant links corresponding to the GTEx eQTLs presented in Figure 2. Of these variants, only one was explained by chromBPNet, and the contribution scores indicated that it disrupted the OCT2 motif, which is not a repressor. However, due to

the limited number of observations, we cannot generalise this finding, and it is possible that other cases may be related to repressors. Further research is needed to confirm this.

2. The results on motif contributions obtained with the help of ChromBPNet (Figure 3) is done within a short 50bp window under a single ATAC-seq peak. Apart from four examples (PPIF in Fig. S10B, which is a negative example as the TT-Seq tracks appear to be identical for both haplotypes; SLFN5 in Fig. S11A; CAV2 in Fig. S11B; PAX8-AS1 in Fig. S11C; and PIK3R5) the result on differential motif contributions are not connected to the differential allele-specific expression. Most of the paragraph looks as an independent study on differential TF binding. What is the statistics of differential TF-binding with differential ASA and ASE? The authors give an idea that this dependence is not simple but do not give any numbers. What is the percentage of cases in which the concordant allele specific expression can be related to a motif disruption? It will be very instructive to see more examples of the kind and in what percentage of cases a differential motif contribution is associated/not associated with differential transcription activity.

Response:

The analyses of the motif contributions were performed within a 50bp window around each variant, and not of single peak as the reviewer mentions. Therefore, each variant within allele-specific peak has been assessed. This was explicitly mentioned in the original version of the manuscript (lines 262-264): “*We then calculated the difference between contribution scores within 50 bp windows around each variant to estimate its local effect (see Methods)*”. As requested by the reviewer, we quantified the statistics of putative regulatory links explained by the TF motif disruptions annotated by chromBPNet, and determined how this relates to concordant and discordant link sets. These results are included in the revised manuscript and are presented in Figure S11: “*We found that 14.6-19.9% of regulatory links could be explained by the predicted TF motif disruptions, of which 51.9-73.9% comprised concordant links (Figure S11A). Notably, both short-range (<50 kb) and long-range (>50 kb) links are identified in our survey (Figure S11B)*”.

2a. By the way, why the authors do not use the 'variant-scorer' function suggested by the authors of ChromBPNet but instead use difference in contribution scores in the 50bp window centred at SNP? Some justification is needed.

Response:

Our inspection of the 'variant-scorer' repository suggests that it has mostly been on GitHub since July 2025. As we originally submitted this work for review in October 2025, we did not consider applying it to this study.

3. Figure 2E implies that simple, window based interaction procedure gives a single ASE-ASA linkage even for large windows. Is this one-to-one ratio true even for large windows, close to 1M in length? If not, is it possible to give a figure of the expected number of ASAs for a single ASE and vice versa, as a function on a window length?

Response:

Our Figure S5B of the original manuscript (Figure S5A of the revised manuscript) showed that there is no one-to-one ratio between accessibility and expression links within the selected 1 Mb window. To better understand the relationship between 1-to-1 and 1-to-many linking, we calculated the fractions of peaks and genes involved in unique (1-to-1) and multiple (1-to-many) links as a function of distance. As shown in Figure S5B of the revised

manuscript, over 80% of peaks and genes involved in 1-many links appear rapidly at a window size of over 100 kb.

I'm not a native English speaker, so it is difficult for me to evaluate the language quality but the paper reads smoothly.
Response:
We appreciate that the reviewer believes our manuscript is well-written.

Reviewer #4 (Remarks to the Author):

A fundamental challenge of genomics is identifying how non-coding regions of the genome regulate gene expression, and especially how genetic variants in these regions can influence gene expression. While GWAS and eQTL studies can be used to identify these associations across large cohorts, methods are lacking to identify these relationships from small numbers of samples. Here the authors demonstrate how they can link specific genomic variants with perturbations in expression of nearby genes using individual human genomes. Their approach relies on integration of whole-chromosome haplotyping with data on chromatin accessibility and transcription. Using a deep learning model, the authors link specific variants to disrupted transcription factor binding motifs. Finally, as a demonstration of the utility of their approach, they investigate a novel variant that disrupts an OCT2 binding side and alters expression of PIK3R5.
Overall, I think the authors have taken an original approach to address an important problem in genomics. The applicability of their method to identifying regulatory relationships for rare variants is especially compelling to me. And I think their manuscript does a good job of describing how they did this work. While I have a few minor comments and questions listed below, I think this work will be well suited to publication in Nature Communications.
Response:
We thank the reviewer for their positive words and encouragement to publish this work in Nature Communications.

MAIN POINTS

1. A key assumption of the authors' approach is that allele-specific gene expression and chromatin accessibility are both sufficiently rare across the genome that when they are found in close proximity to one another, they are likely to be related. This is certainly reasonable, and the authors do a good job of showing that their allele-specific links are much more likely to be near each other (relative to balanced links) and that concordant allele-specific links are near each other. Both of these arguments build a case that these links tend to connect features that are truly related, though as the authors acknowledge, spurious links are nevertheless present in the dataset at some rate. This point probably warrants a bit more discussion. While estimating a rigorous FDR probably isn't feasible, can the others estimate any bounds for the fraction of spurious links that might be present? In addition, while the authors emphasize the enrichment for short-distance links among the allele-specific links, the concordant/discordant fraction seems to suggest that a fair number of these longer-distance links represent real relationships as well.
Response:
We agree with the reviewer that some of the long-range links may represent genuine associations. However, our results show that the majority of functional variant-gene

associations are actually closer than the 1 Mb distance cutoff used in QTL testing. This is consistent with the findings of previous population-based studies (PMID: 24136355, 29988122, 30478436, 39870618 and 39986281) and suggests that long-range distances may not always be necessary to consider. As the distance increases, a concordant link does not necessarily indicate functionality, particularly if the gene has already been linked to another peak at a shorter distance, as is the case in our data (Figures S5A and S5B). In the revised version of the manuscript, we used TAD boundaries as a proxy for the limits of functional interactions, requiring putative regulatory links to be located within the same TAD. Using TAD boundaries as a constraint primarily reduced the number of long-range links. We demonstrate that many of these interactions comprise peaks and genes involved in multiple links (see Figures 2E and S5B of the revised manuscript and our response to Question 2). While we cannot estimate the number of such spurious links exactly, we suggest some ways of mitigating this effect by using TAD boundaries and assessing the link as metrics for evaluating its putative functionality.

2. In the legend to Figure 1D, the authors note that sometimes “genes and peaks may be involved in multiple links”, and this is shown further in Figure S5B. I’m curious about allele-specific peaks that link to multiple genes, and allele-specific genes that link to multiple peaks. There are actually more of these than I anticipated. Are there any interesting biological examples among these? To what extent do you think these represent real versus coincidental links? How do their numbers change if you tighten the distance constraints so that you’re looking at a lower fraction of potentially spurious links as suggested by figure 2E?

Response:

To evaluate the extent to which allele-specific peaks that link to multiple genes, and vice versa, reflect genuine versus coincidental associations, we examined how the prevalence of unique (1-1) versus multi (1-many) links changes with genomic distance. As demonstrated in Figure S5B of the revised manuscript, over 80% of peaks and genes associated with multi-links rapidly increase in number once the window size goes beyond ~100 kb. However, below this threshold, they belong to a unique category. Tightening the distance constraint thus substantially reduces the number of genes and peaks involved in multiple links. Therefore, we believe that 1-to-many links are more likely to reflect spurious long-range correlations, which is consistent with our initial observations.

MINOR POINTS

3. Figure S5B: typo in panel heading.

Response:

We have fixed the typo.

4. Figure 2E: The “concordant/discordant” fraction might initially seem a bit weird for the “balanced” case since they by definition don’t show a significant bias. It might be worth mentioning in the figure legend that no significant bias was detected for these, though they were classified according to the sign of the non-significant difference between them.

Response:

We have added this information to the Figure legend description.

Reviewer #1 (Remarks to the Author):

The authors have addressed my previous questions.
Response:
We are glad to hear that we were able to address the reviewer's questions during revision.

Reviewer #2 (Remarks to the Author):

I thank the authors for their work on addressing my comments.

I have one outstanding concern: point 2 in my original review, which is echoed by Reviewer 3's point 1 and Reviewer 4 point 1. I feel the authors should make it more explicit in the text that their analysis inherently cannot fine-map associations, and therefore all analyses are based on the assumed (rather than inferred) links between the genotypes and molecular phenotypes in their vicinity. Can this be stated clearly in both Results and Discussion?
Response:
We have already indicated that we construct putative regulatory links from the accessibility and expression data by distance association (see Pages 5-6 of the revised manuscript): "We hypothesised that, by linking allele-specific accessibility to gene expression in single individuals, we can predict functional non-coding genetic variants with increased precision. To this end, we constructed putative regulatory links by connecting allele-specific open chromatin peaks and genes over a genomic distance of one megabase". To reiterate this in the Discussion, we now open it with: "*By combining whole-chromosome haplotyping with multiomics assays, we associated non-coding variants from open chromatin regions with gene expression in individual human genomes in an allele-specific manner*".

Minor point: In this context, I wonder if the terminology cis- vs trans-effects is also potentially confusing, because it may be misunderstood as referring to proximal vs distal effects rather than cis-chromosomal and trans-chromosomal. It probably makes sense to amend this accordingly.
Response:
We believe that it is of high importance to discuss the difference between *cis*- (intramolecular, i.e. genetic variation) and *trans*- (intermolecular, i.e. regulatory factor abundance) effects because this describes the rationale for using haplotyping, which is the key element of our study. During the first round of revisions, we provided a definition of *cis*- and *trans*-effects in the Results section to clarify the terminology. We have attempted to make it even more explicit by updating the text: "*Understanding how genetic variants influence gene expression requires separating cis- from trans-acting gene regulatory effects. Combining haplotyping, which allocates variants to their respective alleles, with allele-specific analyses reduces the effects of trans-acting confounders, such as regulatory proteins or RNA, and provides a clearer view of the impact of cis-linked genetic variation*". We have nevertheless removed further discussion of *cis*- and *trans*-effects from the Discussion to avoid any potential confusion as raised by the reviewer.

Reviewer #3 (Remarks to the Author):

The new version of the manuscript is much better. In general, the manuscript is publishable in its current form, I have a small number of minor comments.

1. Page 2. Para 2. "In addition, context-specific regulatory effects can be overlooked in bulk tissues". Probably, the genetic context is discussed. I'm not sure, why the bulk tissue is important here.

Response:

We have removed this sentence to avoid confusion and improve the manuscript flow.

2. The same page 2. "More recently, machine learning models [...] emerged." Please check the grammar. I'm not sure what has emerged here, the machine learning models or the DNA sequence.

Response:

We have removed the “emerged” word to fix the grammar of the sentence.

3. Page 6. Fig. 2E. In fact the number of concordant, putative regulatory, allele specific links found in the same TAD is pretty visible up to 250kb. This is a new and interesting result, which agrees well with LD decaying distance. It is worth to mention this in the text, can be the first estimate of the kind.

Response:

We prefer to refrain from making such conclusions without conducting a proper analysis of this phenomenon, since this has not been the primary focus of this study. Several previous studies have demonstrated that the majority of chromatin interactions identified by Hi-C are not in LD (PMID: 30617125) and that there is no general alignment between LD blocks and TADs (PMID: 30865674). Therefore, while the enrichment of concordant allele-specific links within 250 kb is definitely interesting, it requires further investigation, to make this observation more solid. We feel that this is beyond the scope of our manuscript.

I'm satisfied with the authors' answers to my questions in my first review.

Response:

We are glad that we were able to address the reviewer’s questions during the review process.

Reviewer #4 (Remarks to the Author):

I'm satisfied with the authors' responses to my comments.

Response:

We are glad to hear that we were able to address the reviewer’s questions during revision.
